# Crystal structures of ten enanti­opure Schiff bases bearing a naphthyl group

**DOI:** 10.1107/S2056989016004692

**Published:** 2016-03-31

**Authors:** Guadalupe Hernández-Téllez, Gloria E. Moreno, Sylvain Bernès, Angel Mendoza, Oscar Portillo, Pankaj Sharma, René Gutiérrez

**Affiliations:** a Laboratorio de Síntesis de Complejos, Facultad de Ciencias Químicas, Universidad Autónoma de Puebla, A.P. 1067, 72001 Puebla, Pue., Mexico; b Instituto de Física, Benemérita Universidad Autónoma de Puebla, Av. San Claudio y 18 Sur, 72570 Puebla, Pue., Mexico; c Centro de Química, Instituto de Ciencias, Benemérita Universidad Autónoma de Puebla, 72570 Puebla, Pue., Mexico; d Instituto de Química, Universidad Nacional Autónoma de México, Circuito Exterior s/n, Ciudad Universitaria, Delegación Coyoacán, 04510 México D.F., Mexico

**Keywords:** crystal structure, Schiff base, imine, naphth­yl, chiral compounds

## Abstract

Using a general solvent-free procedure for the synthesis of chiral Schiff bases, the following compounds were synthesized and their crystal structures determined: (*S*)-(+)-2-{[(1-phenyl­eth­yl)imino]­meth­yl}naphthalene, C_19_H_17_N, (**1**), (*S*)-(+)-2-({[(4-methyl­phen­yl)eth­yl]imino}­meth­yl)naphthalene, C_20_H_19_N, (**2**), (*R*)-(−)-2-({[(4-meth­oxy­lphen­yl)eth­yl]imino}­meth­yl)naphthalene, C_20_H_19_NO, (**3**), (*R*)-(−)-2-({[(4-fluoro­phen­yl)eth­yl]imino}­meth­yl)naphthalene, C_19_H_16_FN, (**4**), (*S*)-(+)-2-({[(4-chloro­phen­yl)eth­yl]imino}­meth­yl)naphthalene, C_19_H_16_ClN, (**5**), (*S*)-(+)-2-({[(4-bromo­phen­yl)eth­yl]imino}­meth­yl)naphthalene, C_19_H_16_BrN, (**6**), (*S*)-(+)-2-({[1-(naphthalen-1-yl)eth­yl]imino}­meth­yl)naph­thalene, C_23_H_19_N, (**7**), (*S*)-(+)-2-{[(1-cyclo­hexyl­eth­yl)imino]­meth­yl}naph­tha­lene, C_19_H_23_N, (**8**), (*S*)-(−)-2-{[(1,2,3,4-tetra­hydro­naphthalen-1-yl)imino]meth­yl}naphthalene, C_21_H_19_N, (**9**), and (+)-2-({[(1*S*,2*S*,3*S*,5*R*)-2,6,6-tri­methylbi­cyclo­[3.1.1]hept-3-yl]imino}­meth­yl}naphthalene, C_21_H_25_N, (**10**). The moiety provided by the amine generates conformational flexibility for these imines. In the crystals, no strong inter­molecular contacts are observed, in spite of the presence of aromatic groups.

## Abstract 2

Enanti­opure imines synthesized starting from 2-naphthaldehyde and chiral liquid amines were characterized by X-ray diffraction.

## Chemical context   

Compounds containing an imine group are known to play an important role in living organisms, and some reports have established the presence of imine or azomethine subunits in various natural, natural-derived, and non-natural compounds to be critical to their biological activities (Bringmann *et al.*, 2004 ▸; de Souza *et al.*, 2007 ▸; Guo *et al.*, 2007 ▸). Outside of their biological applications, many Schiff bases also reversibly bind with oxygen, coordinate with and show fluorescent variability with metals, exhibiting photochromism and/or thermochromism, and have been used as catalysts, pigments and dyes, corrosion inhibitors, polymer stabilizers, or precursors in the formation of nanoparticles (Gupta & Sutar, 2008 ▸; Gupta *et al.*, 2009 ▸; Mishra *et al.*, 2012 ▸). As a result of their widespread utility and applications, the search for better and more convenient synthetic routes to Schiff bases improving reaction temperature, time and yields is a never ending trend.

We are currently engaged in a program dedicated to the synthesis of small Schiff bases using a single-step solvent-free approach. Such procedures may overcome, for example, the hydrolytic susceptibility of the formed imine, since water is eliminated as a gas if the Schiff condensation is exothermic. A recent work in this direction was published, which reports the preparation of 15 Schiff bases formed between 3-eth­oxy­salicyl­aldehyde and primary aromatic amines (Tigineh *et al.*, 2015 ▸). In that case, solid reactants were ground in a mortar, first separately and then together. This mechanochemical conversion is efficient (yields > 99%) and can be modified, if necessary, using a liquid-assisted grinding method (Cinčić *et al.*, 2012 ▸) or a solvent-assisted mechanochemical route (Bowmaker, 2013 ▸). Both concepts can even be merged if at least one reactant is a liquid. We used this kind of synthesis for the here reported compounds, using 2-naphthaldehyde (solid, m.p. 331–333 K) and a series of ten chiral liquid primary amines with densities in the range 0.866 to 1.390 g ml^−1^. All of compounds (**1**)–(**10**) were crystallized and their crystal structures confirmed that enanti­opure imines were obtained.
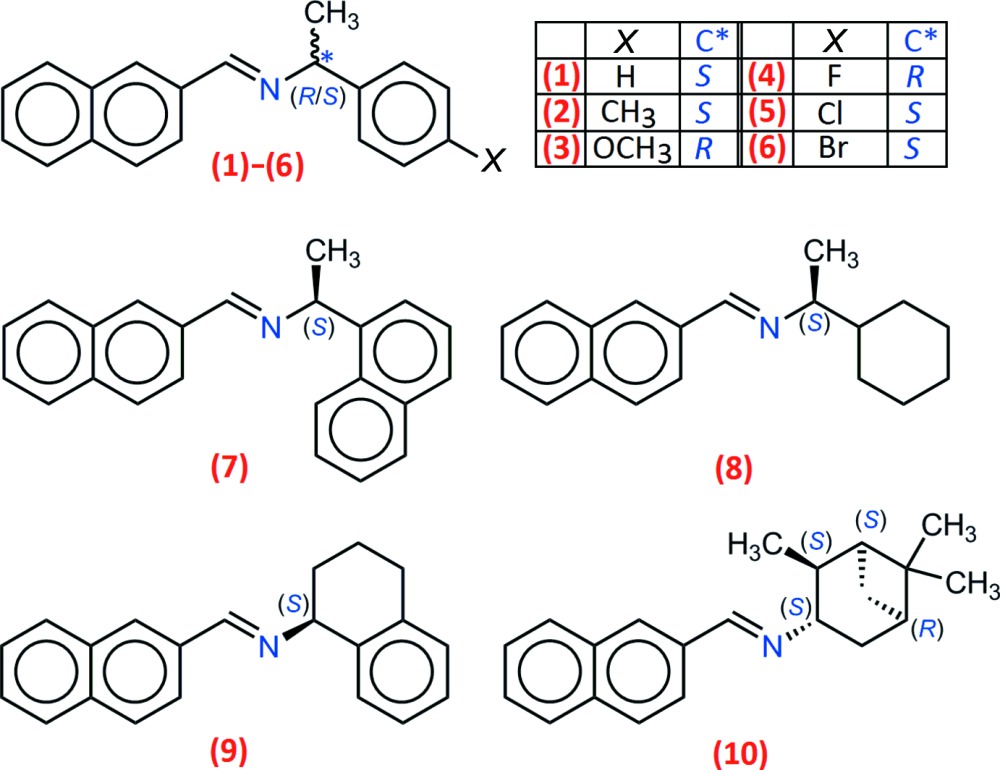


These non-centrosymmetric mol­ecules bearing π-conjugated systems are potentially of inter­est for those who are involved in the synthesis of materials presenting non-linear optical properties. In preliminary tests, a doubling-frequency effect and luminescence have been observed with an Nd:YAG infrared laser (1064 nm) and an UV laser (405 nm), respectively, for eight Schiff bases. These results will be reported elsewhere in due course, along with electrical conduction studies.

## Structural commentary   

**Figure 1:**
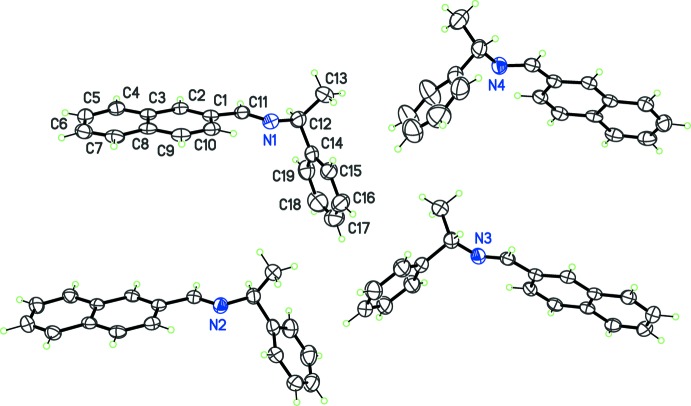
The asymmetric unit of (**1**), with displacement ellipsoids for non-H atoms at the 30% probability level. The labels for C atoms in mol­ecules N2, N3 and N4, are as in mol­ecule N1, but increased by 20, 40, and 60, respectively.

**Figure 2:**
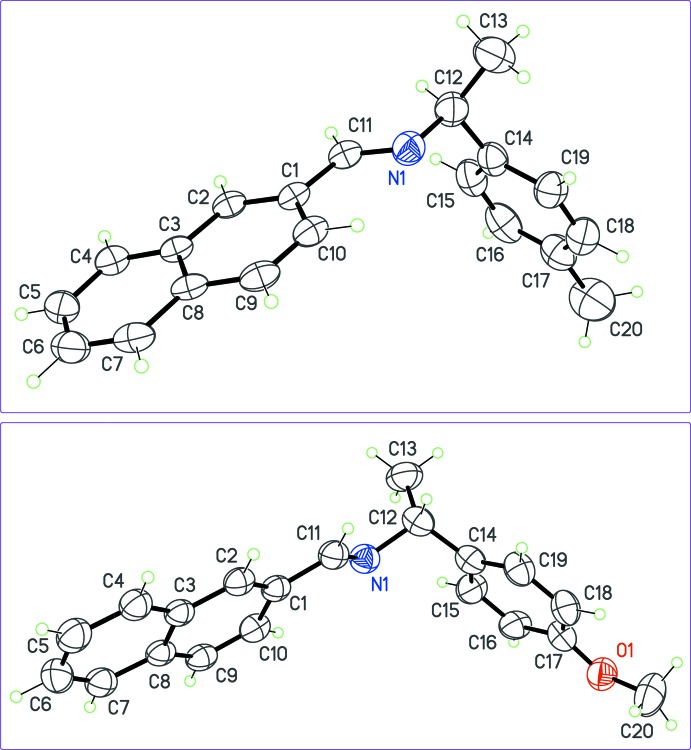
The mol­ecular structures of (**2**) (top) and (**3**) (bottom), with displacement ellipsoids for non-H atoms at the 30% probability level.

As expected, all compounds crystallized in Sohncke space groups, namely *P*2_1_ or *P*2_1_2_1_2_1_. The absolute configuration was determined from anomalous dispersion effects in two crystals, for chlorine and bromine compounds (**5**) and (**6**), confirming that the enanti­opure amines used as starting materials transfer the chiral center to the formed imines, without inversion. The Flack parameters for these crystals converged to 0.02 (6) and −0.009 (6), respectively. For the other compounds, the absolute configuration was assumed from the synthesis. Two compounds crystallize with four mol­ecules per asymmetric unit, (**1**) (Fig. 1 ▸) and (**8**) (Fig. 6), while other compounds are obtained with a single mol­ecule in the asymmetric unit. All imines (**1**)–(**8**) bear a single chiral C atom (C12) presenting the *S* configuration, except for compounds (**3**) and (**4**), which are *R* isomers. The chiral center linking aromatic moieties in (**1**)–(**7**) induces a bent shape for these mol­ecules, and the dihedral angle formed by these moieties may be close to 90°. For instance, in the case of compound (**2**) (Fig. 2 ▸), the naphthyl and benzene rings are inclined to one other at a dihedral angle of 80.49 (7)°

**Figure 4:**
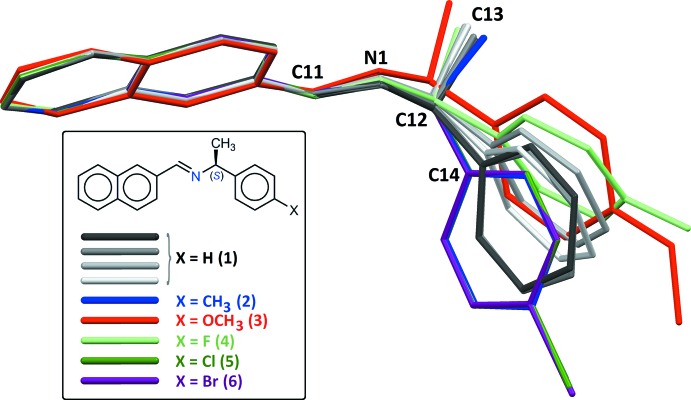
A fit between mol­ecules (**1**)–(**6**), carried out using the naphthyl and imine group atoms (N1/C1–C11). The fit was calculated with the *Structure Overlay* command in *Mercury* (Macrae *et al.*, 2008 ▸), taking as a target the first independent mol­ecule in the first structure (**1**) (dark-gray mol­ecule). For compounds (**3**) and (**4**), the refined model was inverted, in order to fit only *S-*C12 isomers. Note the almost perfect fit obtained for (**2**), (**5**) and (**6**).

**Figure 5:**
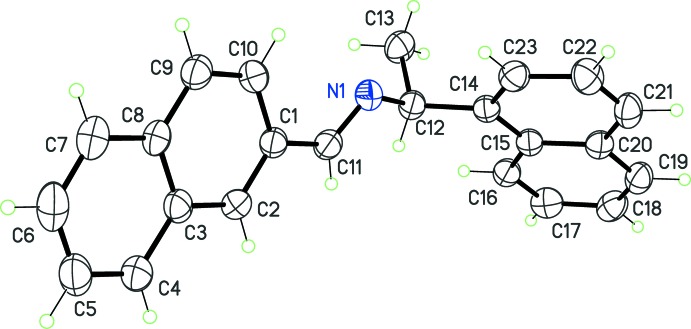
The mol­ecular structure of (**7**), with displacement ellipsoids for non-H atoms at the 30% probability level.

**Figure 6:**
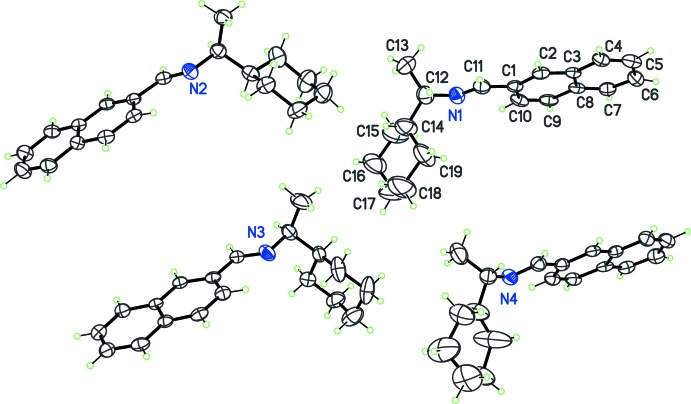
The asymmetric unit of (**8**), with displacement ellipsoids for non-H atoms at the 20% probability level. The labels for C atoms in mol­ecules N2, N3 and N4, are as in mol­ecule N1, but increased by 20, 40, and 60, respectively.

From the chemical point of view, compounds (**1**)–(**6**) are closely related (Figs. 1 ▸–3 ▸ ▸), by modification of the *para* substituent *X* of the phenyl group of phenyl­ethyl­amine. Since *X* is a monoatomic or a small, non-sterically demanding functional group, one could expect that it has no influence on the mol­ecular conformation. However, a fit between the conformers observed in (**1**)–(**6**) (Fig. 4 ▸), shows that the benzene ring has a degree of free rotation about the C12—C14 bond. Such a conformational flexibility can be measured using the torsion angles around C12—C14 and dihedral angles between aromatic rings (Table 1 ▸). Angles N1—C12—C14—C(ring) are in the range 97.9 (5) to 150.1 (5)° for the +*anti*clinal angle τ_1_, and in the range −33.7 (7) to −79.2 (6)° for −*syn*clinal angle τ_2_. For the inter­planar dihedral angle δ, the observed range is from 43.7 (3) to 81.04 (11)°. Inter­estingly, (**2**), (**5**) and (**6**) have very similar metrics, probably as a consequence of the similar steric volumes for CH_3_, Cl, or Br groups. Angles τ_1_, τ_2_ and δ for (**7**) (Fig. 5 ▸) and (**8**) (Fig. 6 ▸) cannot be compared directly with values obtained for (**1**)–(**6**) because C12 is not substituted by a phenyl ring for these compounds (Table 1 ▸, entries 10–14).

**Figure 7:**
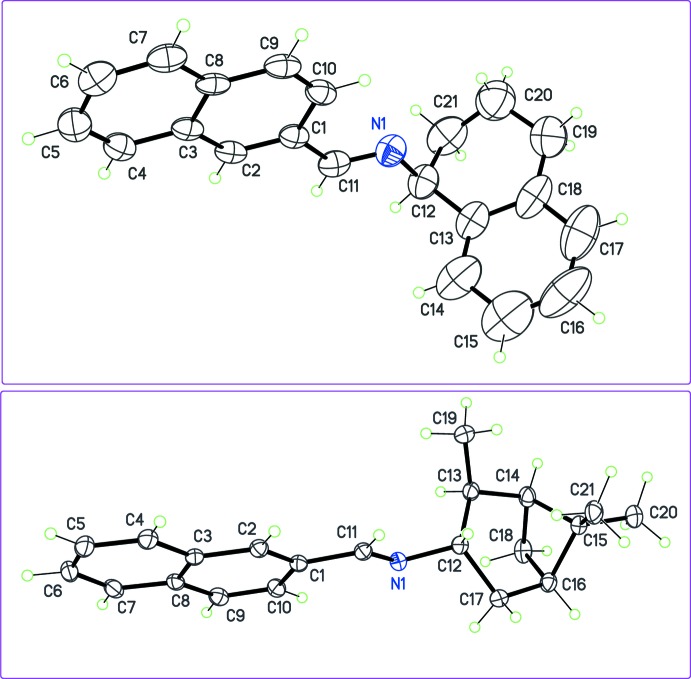
The mol­ecular structures of (**9**) (top) and (**10**) (bottom), with displacement ellipsoids for non-H atoms at the 30% probability level.

Finally, compounds (**9**) and (**10**) (Fig. 7 ▸) are structurally different from (**1**)–(**8**), because the chiral center bonded to the imine group belongs to a cyclic system, introducing a strong restriction to the conformational flexibility.

It is worth noting that in all mol­ecular structures, the imine bond remains conjugated with the naphthyl group. Rotational motions are thus possible only around bonds N1—C12 and C12—C14 for (**1**)–(**8**). For (**9**) and (**10**), only one single bond is involved in conformational flexibility, N1—C12.

## Supra­molecular features   

A common feature may be observed over the series of crystal structures: despite the presence of aromatic systems, the mol­ecules are arranged in such a way that no π–π inter­actions are favored. As an example, in the case of (**1**), which crystallizes with four mol­ecules in the asymmetric unit, the mean planes of naphthyl groups stacked along [100] are separated by more than 6.5 Å. A consequence of the lack of stabilizing inter­molecular forces in these crystals is their quite low packing index, in the range 63.5 [for (**8**)] to 67.0% [for (**10**)], and the occurrence of asymmetric units containing multiple mol­ecules in the case of (**1**) and (**8**). For these compounds, the free rotation for the phenyl (**1**) or cyclo­hexyl (**8**) groups accounts for *Z*′ = 4 asymmetric units. Moreover, (**1**) and (**8**) crystallize in the same space group, *P*2_1_, with similar unit-cell parameters, and similar arrangements for the mol­ecules in the crystal. In other words, the substitution of a planar phenyl group by a non-planar cyclo­hexyl group has little influence on the crystal structure.

The same kind of crystal structure similarity is observed for (**2**), (**3**), (**5**) and (**6**), where the benzene ring is *para*-substituted by non-sterically demanding functional groups, CH_3_, OCH_3_, Cl and Br, respectively. These four compounds crystallize in space group *P*2_1_2_1_2_1_ with unit-cell volumes of *ca* 1600 Å^3^ (see Table 2 ▸). However, the F-based compound, (**4**), is not isomorphous to analoguous compounds bearing Cl (**5**) and Br (**6**). Again, this behavior may be related to the rotational freedom of the benzene ring, which modifies the mol­ecular conformation stabilized in the solid state.

Indeed, the poor ability of the naphthyl group for the formation of π–π contacts seems to be a general rule. The crystal structures reported here cannot be compared to literature data, since no chiral secondary aldimine bearing a 2-naphthyl group on the C-side have been X-ray characterized up to now, and only a few cases are available with substituted naphthyl groups, generally related to *BINOL* derivatives (Li *et al.*, 2004 ▸). However, small achiral Schiff bases including naphthyl (Blanco *et al.*, 2012 ▸), or naphthol (Fernández-G *et al.*, 1995 ▸, 2001 ▸; Martínez *et al.*, 2011 ▸) have been reported. For these crystal structures, a general propensity to form C—H⋯π inter­molecular contacts rather than π–π contacts is observed.

## Database survey   

The structure of 2-naphthaldehyde was almost certainly determined but never reported. Neither are the crystal structures for the used amines available, since all are liquid at room temperature. Crystal structures for imines derived from 2-naphthaldehyde are also very scarce, and related to the chemistry of Schiff bases. Chiral 2-naphthaldehyde oxime derivatives have been synthesized as precursors of oxime ethers useful for the asymmetric synthesis of *N*-protected amines and *β*-amino acids (Hunt *et al.*, 1999 ▸). The structure of a radical compound bearing the 2-naphthyl­methyl­ene­amino group has also been determined (Iwasaki *et al.*, 1999 ▸), with the aim of rationalizing the ferromagnetic inter­actions in this compound, which presents a magnetic phase transition at 0.12 K (Ishida *et al.*, 1995 ▸). Finally, the structure of an hydrazide with the 2-naphthyl­methyl­ene group is known (Qiu *et al.*, 2006 ▸).

Regarding imines built on the amines used in this work, X-ray structures have been deposited in the CSD (Groom & Allen, 2014 ▸) for compounds derived from 1-phenyl­ethyl­amine [as for (**1**)] and 1-cyclo­hexyl­ethyl­amine [as in (**8**)]. For others, only sporadic X-ray determinations are carried out, for example for salicylaldimines (Enamullah *et al.*, 2007 ▸). We thus think that there is room for improvement in the knowledge of the chemical crystallography of this class of compounds, taking into account that many of them are easy to prepare.

## Synthesis and crystallization   

The reaction of optically pure primary amines with 2-naphthaldehyde to yield chiral Schiff bases (**1**)–(**10**) was performed under solvent-free conditions. Reactants were mixed for 3–5 min. using a mortar and pestle, yielding oily products that become solids standing on air for an additional 2–3 minutes. The reaction was monitored by TLC and ^1^H NMR, observing the disappearance of the aldehyde. Because of the direct inter­action and because no excess of reagents were used, the products were obtained with no waste and no further purification processes were needed. In most cases the products were obtained in a sufficiently pure form, or a simple crystallization was enough, when necessary, and in our case, the obtained crude products were recrystallized from CH_2_Cl_2_, affording the corresponding pure Schiff bases (**1**)–(**10**) as crystals of good quality for X-ray studies.

The IR spectra (**1**)–(**10**) showed characteristic absorption bands in the 1635–1626 cm^−1^ range, due to the C=N stretching vibration, in agreement with reported values. In the ^1^H NMR spectra, the azomethine proton appears in the 8.33–8.51 p.p.m. range, while the imine bond is characterized in the ^13^C NMR spectra with the imine C signal in the 157.9–160.7 p.p.m. range. Full spectroscopic data are available in the supporting information.

## Refinement   

Crystal data, data collection and structure refinement details are summarized in Table 2 ▸. Monoclinic crystals for compounds (**1**) and (**8**) were twinned by a twofold rotation, with the same twin law [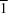 0 0, 0 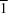 0, 1 0 1]. Twin weights are 0.42/0.58 for (**1**) and 0.11/0.89 for (**8**). Some structures [in particular (**4**), (**8**) and (**9**)] converged towards rather disappointing refinements, as a result of packing issues and disordered parts that were not well resolved. For (**8**), two cyclo­hexyl rings, C14–C19 and C74–C79, were restrained to have the same bond lengths as those of ring C34–C39. Other structures were refined without restraints.

For (**1**)–(**9**), H atoms were placed in idealized positions, with C—H bond lengths fixed at 0.93 (aromatic), 0.96 (meth­yl), 0.97 (methyl­ene) or 0.98 Å (methine). For (**10**), collected at 150 K, these distances were fixed at 0.95, 0.98, 0.99 and 1.00 Å, respectively. In all compounds, isotropic displacement parameters for H atoms were calculated as *U*_iso_(H) = *xU*_eq_(carrier atom) with *x* = 1.5 (meth­yl) or *x* = 1.2 (methyl­ene, methine, aromatic).

## Supplementary Material

Crystal structure: contains datablock(s) 1, 2, 3, 4, 5, 6, 7, 8, 9, 10, global. DOI: 10.1107/S2056989016004692/hb7571sup1.cif

Structure factors: contains datablock(s) 1. DOI: 10.1107/S2056989016004692/hb75711sup2.hkl

Structure factors: contains datablock(s) 2. DOI: 10.1107/S2056989016004692/hb75712sup3.hkl

Structure factors: contains datablock(s) 3. DOI: 10.1107/S2056989016004692/hb75713sup4.hkl

Structure factors: contains datablock(s) 4. DOI: 10.1107/S2056989016004692/hb75714sup5.hkl

Structure factors: contains datablock(s) 5. DOI: 10.1107/S2056989016004692/hb75715sup6.hkl

Structure factors: contains datablock(s) 6. DOI: 10.1107/S2056989016004692/hb75716sup7.hkl

Structure factors: contains datablock(s) 7. DOI: 10.1107/S2056989016004692/hb75717sup8.hkl

Structure factors: contains datablock(s) 8. DOI: 10.1107/S2056989016004692/hb75718sup9.hkl

Structure factors: contains datablock(s) 9. DOI: 10.1107/S2056989016004692/hb75719sup10.hkl

Structure factors: contains datablock(s) 10. DOI: 10.1107/S2056989016004692/hb757110sup11.hkl

Supporting information file. DOI: 10.1107/S2056989016004692/hb75711sup12.cml

Supporting information file. DOI: 10.1107/S2056989016004692/hb75712sup13.cml

Supporting information file. DOI: 10.1107/S2056989016004692/hb75713sup14.cml

Supporting information file. DOI: 10.1107/S2056989016004692/hb75714sup15.cml

Supporting information file. DOI: 10.1107/S2056989016004692/hb75715sup16.cml

Supporting information file. DOI: 10.1107/S2056989016004692/hb75716sup17.cml

Supporting information file. DOI: 10.1107/S2056989016004692/hb75717sup18.cml

Supporting information file. DOI: 10.1107/S2056989016004692/hb75718sup19.cml

Supporting information file. DOI: 10.1107/S2056989016004692/hb75719sup20.cml

Supporting information file. DOI: 10.1107/S2056989016004692/hb757110sup21.cml

Supporting information file. DOI: 10.1107/S2056989016004692/hb7571sup22.pdf

CCDC references: 1425668, 1425667, 1425666, 1425665, 1425664, 1425663, 1425662, 1425661, 1425660, 1425659

Additional supporting information:  crystallographic information; 3D view; checkCIF report

## Figures and Tables

**Figure 3:**
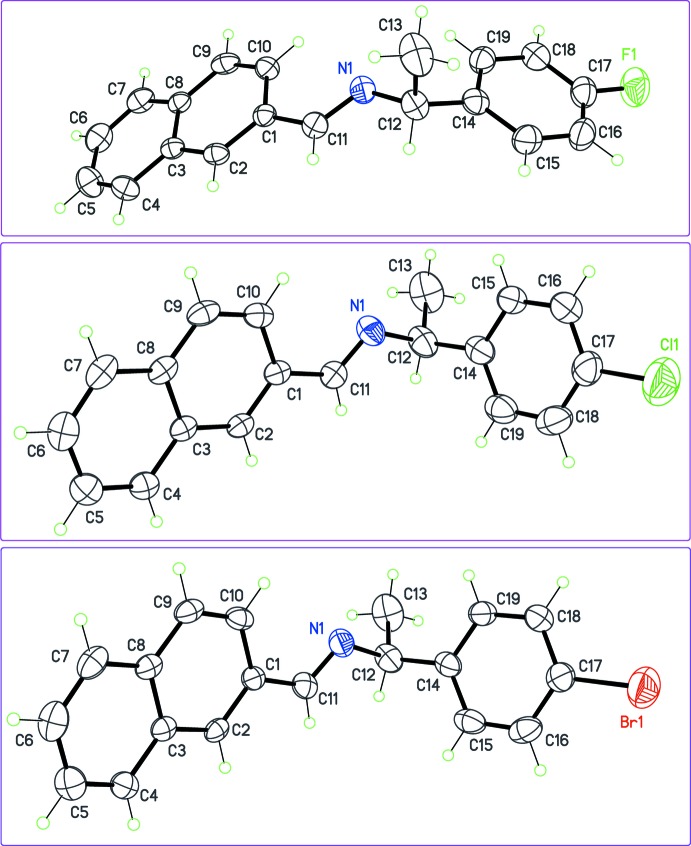
The mol­ecular structures of halogenated imines (**4**) (top), (**5**) (middle), and (**6**) (bottom), with displacement ellipsoids for non-H atoms at the 30% probability level. Note the different *S*/*R* configuration at C12 for (**4**) compared to (**5**) and (**6**).

**Table 1:** Relative orientation (°) between the naphthyl group and the ring bonded to the chiral C atom C12 in compounds (**1**)–(**8**) Angles τ_1_ and τ_2_ are torsion angles N1—C12—C14—C(ring) or equivalent angles for compounds (**1**) and (**8**), which have four mol­ecules in the asymmetric unit. The dihedral angle δ is calculated between the mean planes of the naphthyl group and the ring bonded to C12.

Mol­ecule	τ_1_ (+*ac*)	τ_2_ (−*sc*)	δ
(**1**)/N1	110.0 (5)	−67.2 (5)	78.22 (11)
(**1**)/N2	140.7 (4)	−41.5 (5)	58.23 (11)
(**1**)/N3	142.0 (4)	−40.0 (6)	43.7 (3)
(**1**)/N4	110.0 (6)	−64.6 (8)	74.97 (18)
(**2**)	98.3 (4)	−77.7 (4)	80.49 (7)
(**3**)^*a*^	111.5 (5)	−67.0 (6)	60.16 (13)
(**4**)^*a*^	150.1 (5)	−33.7 (7)	45.91 (15)
(**5**)	97.9 (5)	−77.9 (6)	80.99 (10)
(**6**)	98.1 (6)	−79.2 (6)	81.04 (11)
			
(**7**)^*b*^	155.4 (3)	−27.4 (4)	67.44 (6)
(**8**)/N1	60 (2)	−66 (2)	88.6 (6)
(**8**)/N2	169.6 (9)	−62.6 (12)	48.0 (3)
(**8**)/N3	70.0 (12)	−55.6 (13)	88.2 (3)
(**8**)/N4	165.1 (14)	22 (3)	66.7 (6)

Notes: (*a*) Refined model has been inverted in order to compare *S*-C12 mol­ecules. (*b*) The dihedral angle δ is computed between the two naphthyl ring systems.

**Table:** 

	(**1**)	(**2**)	(**3**)
Crystal data			
Chemical formula	C_19_H_17_N	C_20_H_19_N	C_20_H_19_NO
*M*_r_	259.33	273.36	289.36
Crystal system, space group	Monoclinic, *P*2_1_	Orthorhombic, *P*2_1_2_1_2_1_	Orthorhombic, *P*2_1_2_1_2_1_
Temperature (K)	298	298	298
*a*, *b*, *c* (Å)	14.9328 (2), 6.01143 (10), 33.9985 (7)	6.0946 (5), 7.5732 (5), 34.046 (3)	6.1094 (5), 7.7266 (7), 34.225 (4)
α, β, γ (°)	90, 102.6011 (17), 90	90, 90, 90	90, 90, 90
*V* (Å^3^)	2978.45 (9)	1571.4 (2)	1615.6 (3)
*Z*	8	4	4
Radiation type	Cu *K*α	Cu *K*α	Cu *K*α
μ (mm^−1^)	0.51	0.51	0.57
Crystal size (mm)	0.49 × 0.17 × 0.10	0.49 × 0.13 × 0.05	0.39 × 0.25 × 0.23

Data collection			
Diffractometer	Agilent Xcalibur Atlas Gemini	Agilent Xcalibur Atlas Gemini	Agilent Xcalibur Atlas Gemini
Absorption correction	Analytical (*CrysAlis PRO*; Agilent, 2013 ▸)	Analytical (*CrysAlis PRO*; Agilent, 2013 ▸)	Multi-scan (*CrysAlis PRO*; Agilent, 2013 ▸)
*T*_min_, *T*_max_	0.862, 0.960	0.793, 0.969	0.777, 1.000
No. of measured, independent and observed [*I* > 2σ(*I*)] reflections	33420, 10553, 9322	13738, 2770, 2048	14921, 3223, 1652
*R*_int_	0.045	0.041	0.068
(sin θ/λ)_max_ (Å^−1^)	0.622	0.595	0.624

Refinement			
*R*[*F* ^2^ > 2σ(*F* ^2^)], *wR*(*F* ^2^), *S*	0.039, 0.092, 1.04	0.044, 0.112, 1.08	0.063, 0.155, 1.08
No. of reflections	10553	2770	3223
No. of parameters	722	192	201
No. of restraints	1	0	0
H-atom treatment	H-atom parameters constrained	H-atom parameters constrained	H-atom parameters constrained
Δρ_max_, Δρ_min_ (e Å^−3^)	0.13, −0.12	0.09, −0.11	0.15, −0.20

**Table:** 

	(**4**)	(**5**)	(**6**)
Crystal data			
Chemical formula	C_19_H_16_FN	C_19_H_16_ClN	C_19_H_16_BrN
*M*_r_	277.33	293.78	338.24
Crystal system, space group	Monoclinic, *P*2_1_	Orthorhombic, *P*2_1_2_1_2_1_	Orthorhombic, *P*2_1_2_1_2_1_
Temperature (K)	298	298	298
*a*, *b*, *c* (Å)	7.5950 (11), 5.8997 (9), 16.996 (3)	6.0567 (5), 7.6139 (5), 33.853 (3)	6.0526 (2), 7.6671 (4), 33.9712 (19)
α, β, γ (°)	90, 99.420 (15), 90	90, 90, 90	90, 90, 90
*V* (Å^3^)	751.3 (2)	1561.1 (2)	1576.46 (13)
*Z*	2	4	4
Radiation type	Mo *K*α	Mo *K*α	Mo *K*α
μ (mm^−1^)	0.08	0.24	2.60
Crystal size (mm)	0.74 × 0.21 × 0.09	0.38 × 0.32 × 0.14	0.36 × 0.22 × 0.19

Data collection			
Diffractometer	Agilent Xcalibur Atlas Gemini	Agilent Xcalibur Atlas Gemini	Agilent Xcalibur Atlas Gemini
Absorption correction	Analytical (*CrysAlis PRO*; Agilent, 2013 ▸)	Analytical (*CrysAlis PRO*; Agilent, 2013 ▸)	Analytical (*CrysAlis PRO*; Agilent, 2013 ▸)
*T*_min_, *T*_max_	0.970, 0.994	0.437, 0.703	0.876, 0.920
No. of measured, independent and observed [*I* > 2σ(*I*)] reflections	7823, 2627, 1640	21167, 2752, 2014	19221, 3120, 2017
*R*_int_	0.051	0.065	0.047
(sin θ/λ)_max_ (Å^−1^)	0.595	0.595	0.618

Refinement			
*R*[*F* ^2^ > 2σ(*F* ^2^)], *wR*(*F* ^2^), *S*	0.066, 0.153, 1.25	0.057, 0.139, 1.07	0.044, 0.098, 1.02
No. of reflections	2627	2752	3120
No. of parameters	190	191	191
No. of restraints	1	0	0
H-atom treatment	H-atom parameters constrained	H-atom parameters constrained	H-atom parameters constrained
Δρ_max_, Δρ_min_ (e Å^−3^)	0.23, −0.19	0.18, −0.29	0.32, −0.44
Absolute structure	–	Flack *x* determined using 645 quotients [(*I* ^+^)−(*I* ^−^)]/[(*I* ^+^)+(*I* ^−^)] (Parsons *et al.*, 2013 ▸)	Flack *x* determined using 643 quotients [(*I* ^+^)−(*I* ^−^)]/[(*I* ^+^)+(*I* ^−^)] (Parsons *et al.*, 2013 ▸)
Absolute structure parameter	–	0.02 (6)	−0.009 (6)

**Table:** 

	(**7**)	(**8**)
Crystal data		
Chemical formula	C_23_H_19_N	C_19_H_23_N
*M*_r_	309.39	265.38
Crystal system, space group	Monoclinic, *P*2_1_	Monoclinic, *P*2_1_
Temperature (K)	298	298
*a*, *b*, *c* (Å)	7.8555 (5), 7.8724 (4), 14.0494 (9)	15.406 (3), 5.9722 (7), 36.002 (7)
α, β, γ (°)	90, 99.859 (6), 90	90, 102.058 (18), 90
*V* (Å^3^)	856.01 (9)	3239.4 (10)
*Z*	2	8
Radiation type	Mo *K*α	Mo *K*α
μ (mm^−1^)	0.07	0.06
Crystal size (mm)	0.45 × 0.35 × 0.11	0.54 × 0.09 × 0.07

Data collection		
Diffractometer	Agilent Xcalibur Atlas Gemini	Agilent Xcalibur Atlas Gemini
Absorption correction	Multi-scan (*CrysAlis PRO*; Agilent, 2013 ▸)	Analytical (*CrysAlis PRO*; Agilent, 2013 ▸)
*T*_min_, *T*_max_	0.921, 1.000	0.995, 0.999
No. of measured, independent and observed [*I* > 2σ(*I*)] reflections	9508, 3367, 2063	19804, 10600, 3810
*R*_int_	0.043	0.119
(sin θ/λ)_max_ (Å^−1^)	0.625	0.595

Refinement		
*R*[*F* ^2^ > 2σ(*F* ^2^)], *wR*(*F* ^2^), *S*	0.048, 0.119, 1.01	0.078, 0.231, 0.99
No. of reflections	3367	10600
No. of parameters	218	727
No. of restraints	1	37
H-atom treatment	H-atom parameters constrained	H-atom parameters constrained
Δρ_max_, Δρ_min_ (e Å^−3^)	0.12, −0.14	0.23, −0.18

**Table:** 

	(**9**)	(**10**)
Crystal data		
Chemical formula	C_21_H_19_N	C_21_H_25_N
*M*_r_	285.37	291.42
Crystal system, space group	Monoclinic, *P*2_1_	Orthorhombic, *P*2_1_2_1_2_1_
Temperature (K)	298	150
*a*, *b*, *c* (Å)	7.7571 (13), 5.9246 (10), 17.820 (4)	6.32427 (18), 14.4559 (3), 18.5421 (5)
α, β, γ (°)	90, 92.682 (16), 90	90, 90, 90
*V* (Å^3^)	818.1 (2)	1695.17 (8)
*Z*	2	4
Radiation type	Mo *K*α	Cu *K*α
μ (mm^−1^)	0.07	0.49
Crystal size (mm)	0.88 × 0.43 × 0.08	0.40 × 0.15 × 0.12

Data collection		
Diffractometer	Agilent Xcalibur Atlas Gemini	Agilent Xcalibur Atlas Gemini
Absorption correction	Analytical (*CrysAlis PRO*; Agilent, 2013 ▸)	Multi-scan (*CrysAlis PRO*; Agilent, 2013 ▸)
*T*_min_, *T*_max_	0.857, 0.983	0.976, 1.000
No. of measured, independent and observed [*I* > 2σ(*I*)] reflections	15266, 2884, 1822	19648, 3417, 2913
*R*_int_	0.081	0.056
(sin θ/λ)_max_ (Å^−1^)	0.595	0.624

Refinement		
*R*[*F* ^2^ > 2σ(*F* ^2^)], *wR*(*F* ^2^), *S*	0.069, 0.175, 1.44	0.041, 0.103, 1.03
No. of reflections	2884	3417
No. of parameters	199	202
No. of restraints	1	0
H-atom treatment	H-atom parameters constrained	H-atom parameters constrained
Δρ_max_, Δρ_min_ (e Å^−3^)	0.21, −0.19	0.19, −0.17

Computer programs: *CrysAlis PRO* (Agilent, 2013 ▸), *SHELXS2013* and *SHELXTL* (Sheldrick, 2008 ▸), *SHELXL2014* (Sheldrick, 2015 ▸) and *CifTab* (Sheldrick, 2015 ▸).

## supplementary crystallographic information

### (1) (S)-(+)-2-{[(1-Phenylethyl)imino]methyl}naphthalene Crystal data

**Table:**

C_19_H_17_N	*D*_x_ = 1.157 Mg m^−^^3^
*M**_r_* = 259.33	Melting point: 389 K
Monoclinic, *P*2_1_	Cu *K*α radiation, λ = 1.54184 Å
*a* = 14.9328 (2) Å	Cell parameters from 11908 reflections
*b* = 6.01143 (10) Å	θ = 3.6–74.0°
*c* = 33.9985 (7) Å	µ = 0.51 mm^−^^1^
β = 102.6011 (17)°	*T* = 298 K
*V* = 2978.45 (9) Å^3^	Prism, colorless
*Z* = 8	0.49 × 0.17 × 0.10 mm
*F*(000) = 1104	

### (1) (S)-(+)-2-{[(1-Phenylethyl)imino]methyl}naphthalene Data collection

**Table:**

Agilent Xcalibur Atlas Gemini diffractometer	10553 independent reflections
Radiation source: Enhance (Cu) X-ray Source	9322 reflections with *I* > 2σ(*I*)
Graphite monochromator	*R*_int_ = 0.045
Detector resolution: 10.5564 pixels mm^-1^	θ_max_ = 73.7°, θ_min_ = 3.0°
ω scans	*h* = −18→18
Absorption correction: analytical (CrysAlis PRO; Agilent, 2013)	*k* = −7→6
*T*_min_ = 0.862, *T*_max_ = 0.960	*l* = −42→42
33420 measured reflections	

### (1) (S)-(+)-2-{[(1-Phenylethyl)imino]methyl}naphthalene Refinement

**Table:**

Refinement on *F*^2^	Primary atom site location: structure-invariant direct methods
Least-squares matrix: full	Secondary atom site location: difference Fourier map
*R*[*F*^2^ > 2σ(*F*^2^)] = 0.039	Hydrogen site location: inferred from neighbouring sites
*wR*(*F*^2^) = 0.092	H-atom parameters constrained
*S* = 1.04	*w* = 1/[σ^2^(*F*_o_^2^) + (0.0357*P*)^2^ + 0.0936*P*] where *P* = (*F*_o_^2^ + 2*F*_c_^2^)/3
10553 reflections	(Δ/σ)_max_ < 0.001
722 parameters	Δρ_max_ = 0.13 e Å^−^^3^
1 restraint	Δρ_min_ = −0.12 e Å^−^^3^
0 constraints	

### (1) (S)-(+)-2-{[(1-Phenylethyl)imino]methyl}naphthalene Special details

**Table:**

Refinement. Refined as a 2-component twin.

### (1) (S)-(+)-2-{[(1-Phenylethyl)imino]methyl}naphthalene Fractional atomic coordinates and isotropic or equivalent isotropic displacement parameters (Å^2^)

**Table:**

	*x*	*y*	*z*	*U*_iso_*/*U*_eq_	
N1	0.3821 (2)	0.6149 (7)	0.12375 (10)	0.0691 (8)	
C1	0.36179 (19)	0.5467 (6)	0.05277 (10)	0.0517 (7)	
C2	0.33602 (19)	0.6359 (5)	0.01504 (10)	0.0489 (6)	
H2A	0.3079	0.7747	0.0118	0.059*	
C3	0.35134 (18)	0.5212 (5)	−0.01922 (10)	0.0484 (6)	
C4	0.3294 (2)	0.6113 (6)	−0.05836 (12)	0.0587 (8)	
H4A	0.2996	0.7479	−0.0626	0.070*	
C5	0.3509 (3)	0.5030 (8)	−0.09004 (11)	0.0693 (10)	
H5A	0.3367	0.5671	−0.1155	0.083*	
C6	0.3945 (3)	0.2943 (9)	−0.08457 (14)	0.0726 (11)	
H6A	0.4101	0.2218	−0.1063	0.087*	
C7	0.4139 (2)	0.1987 (7)	−0.04728 (13)	0.0654 (9)	
H7A	0.4409	0.0586	−0.0441	0.078*	
C8	0.39396 (18)	0.3070 (6)	−0.01371 (11)	0.0518 (7)	
C9	0.4167 (2)	0.2169 (6)	0.02562 (12)	0.0571 (8)	
H9A	0.4425	0.0757	0.0294	0.069*	
C10	0.4016 (2)	0.3322 (6)	0.05801 (11)	0.0567 (7)	
H10A	0.4175	0.2699	0.0836	0.068*	
C11	0.3529 (2)	0.6828 (7)	0.08803 (10)	0.0572 (8)	
H11A	0.3250	0.8217	0.0838	0.069*	
C12	0.3764 (3)	0.7728 (10)	0.15620 (13)	0.0813 (13)	
H12A	0.3492	0.9126	0.1444	0.098*	
C13	0.3142 (3)	0.6688 (17)	0.18172 (17)	0.125 (3)	
H13A	0.3092	0.7684	0.2032	0.188*	
H13B	0.2544	0.6425	0.1651	0.188*	
H13C	0.3402	0.5304	0.1928	0.188*	
C14	0.4726 (3)	0.8153 (8)	0.17974 (11)	0.0680 (9)	
C15	0.5234 (3)	0.6523 (9)	0.20243 (13)	0.0803 (11)	
H15A	0.4965	0.5137	0.2038	0.096*	
C16	0.6118 (4)	0.6838 (14)	0.22324 (16)	0.104 (2)	
H16A	0.6437	0.5694	0.2387	0.125*	
C17	0.6526 (4)	0.8840 (14)	0.2211 (2)	0.111 (2)	
H17A	0.7129	0.9070	0.2348	0.133*	
C18	0.6054 (5)	1.0499 (14)	0.1989 (2)	0.116 (2)	
H18A	0.6333	1.1871	0.1976	0.140*	
C19	0.5146 (4)	1.0165 (10)	0.17805 (16)	0.0916 (13)	
H19A	0.4827	1.1318	0.1629	0.110*	
N2	0.8709 (2)	0.5109 (6)	0.10287 (9)	0.0609 (7)	
C21	0.86187 (19)	0.5227 (6)	0.03158 (9)	0.0477 (6)	
C22	0.83652 (18)	0.6499 (6)	−0.00250 (9)	0.0472 (6)	
H22A	0.8084	0.7867	−0.0010	0.057*	
C23	0.85254 (19)	0.5763 (6)	−0.04015 (10)	0.0473 (7)	
C24	0.8294 (2)	0.7087 (7)	−0.07530 (11)	0.0587 (8)	
H24A	0.8012	0.8460	−0.0744	0.070*	
C25	0.8484 (3)	0.6352 (9)	−0.11067 (11)	0.0713 (10)	
H25A	0.8327	0.7227	−0.1337	0.086*	
C26	0.8913 (3)	0.4295 (9)	−0.11252 (11)	0.0713 (10)	
H26A	0.9045	0.3823	−0.1367	0.086*	
C27	0.9137 (2)	0.2985 (7)	−0.07933 (12)	0.0611 (8)	
H27A	0.9418	0.1620	−0.0810	0.073*	
C28	0.89465 (18)	0.3671 (6)	−0.04203 (10)	0.0491 (7)	
C29	0.9181 (2)	0.2384 (6)	−0.00648 (11)	0.0527 (7)	
H29A	0.9445	0.0991	−0.0075	0.063*	
C30	0.9028 (2)	0.3138 (6)	0.02941 (10)	0.0523 (7)	
H30A	0.9194	0.2267	0.0524	0.063*	
C31	0.8472 (2)	0.6129 (6)	0.07003 (10)	0.0505 (7)	
H31A	0.8192	0.7511	0.0699	0.061*	
C32	0.8530 (3)	0.6215 (8)	0.13915 (10)	0.0645 (9)	
H32A	0.8228	0.7645	0.1314	0.077*	
C33	0.7889 (3)	0.4731 (11)	0.15689 (12)	0.0841 (13)	
H33A	0.7764	0.5420	0.1806	0.126*	
H33B	0.7325	0.4525	0.1374	0.126*	
H33C	0.8176	0.3313	0.1639	0.126*	
C34	0.9416 (2)	0.6614 (7)	0.16909 (9)	0.0612 (8)	
C35	0.9537 (4)	0.8582 (9)	0.19069 (13)	0.0822 (11)	
H35A	0.9076	0.9651	0.1861	0.099*	
C36	1.0340 (4)	0.8977 (11)	0.21920 (14)	0.0984 (16)	
H36A	1.0414	1.0313	0.2333	0.118*	
C37	1.1019 (4)	0.7420 (12)	0.22665 (14)	0.0954 (17)	
H37A	1.1554	0.7685	0.2459	0.115*	
C38	1.0906 (3)	0.5450 (10)	0.20537 (13)	0.0819 (12)	
H38A	1.1369	0.4384	0.2102	0.098*	
C39	1.0103 (3)	0.5050 (8)	0.17674 (11)	0.0670 (9)	
H39A	1.0030	0.3713	0.1626	0.080*	
N3	0.7737 (2)	0.5492 (6)	0.39508 (9)	0.0652 (7)	
C41	0.8320 (2)	0.5196 (6)	0.46632 (10)	0.0507 (7)	
C42	0.8391 (2)	0.3881 (6)	0.49968 (10)	0.0497 (6)	
H42A	0.8097	0.2508	0.4972	0.060*	
C43	0.89007 (19)	0.4564 (5)	0.53793 (10)	0.0487 (7)	
C44	0.8999 (2)	0.3201 (7)	0.57233 (11)	0.0590 (8)	
H44A	0.8704	0.1830	0.5704	0.071*	
C45	0.9519 (3)	0.3873 (9)	0.60815 (12)	0.0748 (11)	
H45A	0.9578	0.2957	0.6306	0.090*	
C46	0.9970 (3)	0.5927 (9)	0.61173 (12)	0.0740 (11)	
H46A	1.0335	0.6350	0.6364	0.089*	
C47	0.9877 (2)	0.7321 (7)	0.57922 (12)	0.0614 (8)	
H47A	1.0167	0.8700	0.5821	0.074*	
C48	0.9341 (2)	0.6669 (6)	0.54124 (10)	0.0512 (7)	
C49	0.9242 (2)	0.8019 (6)	0.50660 (11)	0.0545 (7)	
H49A	0.9516	0.9415	0.5086	0.065*	
C50	0.8751 (2)	0.7305 (6)	0.47012 (10)	0.0540 (7)	
H50A	0.8699	0.8213	0.4476	0.065*	
C51	0.7827 (2)	0.4360 (7)	0.42688 (11)	0.0556 (8)	
H51A	0.7571	0.2944	0.4254	0.067*	
C52	0.7257 (3)	0.4463 (9)	0.35727 (11)	0.0717 (10)	
H52A	0.7092	0.2928	0.3623	0.086*	
C53	0.6389 (3)	0.5813 (15)	0.34092 (14)	0.107 (2)	
H53A	0.6062	0.5168	0.3161	0.161*	
H53B	0.6005	0.5802	0.3602	0.161*	
H53C	0.6553	0.7318	0.3362	0.161*	
C54	0.7881 (2)	0.4484 (7)	0.32731 (10)	0.0625 (8)	
C55	0.7900 (3)	0.2742 (9)	0.30191 (13)	0.0816 (11)	
H55A	0.7537	0.1500	0.3033	0.098*	
C56	0.8453 (4)	0.2788 (11)	0.27403 (14)	0.0933 (16)	
H56A	0.8462	0.1570	0.2573	0.112*	
C57	0.8982 (3)	0.4596 (12)	0.27090 (13)	0.0930 (16)	
H57A	0.9346	0.4633	0.2519	0.112*	
C58	0.8966 (3)	0.6345 (11)	0.29607 (13)	0.0908 (14)	
H58A	0.9326	0.7589	0.2943	0.109*	
C59	0.8428 (3)	0.6304 (9)	0.32411 (13)	0.0800 (11)	
H59A	0.8431	0.7515	0.3412	0.096*	
N4	0.2651 (2)	0.3804 (7)	0.37650 (10)	0.0689 (8)	
C61	0.3102 (2)	0.4677 (6)	0.44685 (11)	0.0517 (7)	
C62	0.3211 (2)	0.3891 (6)	0.48543 (10)	0.0506 (7)	
H62A	0.2969	0.2509	0.4897	0.061*	
C63	0.3682 (2)	0.5138 (6)	0.51895 (10)	0.0520 (7)	
C64	0.3834 (3)	0.4321 (7)	0.55866 (12)	0.0627 (8)	
H64A	0.3582	0.2963	0.5637	0.075*	
C65	0.4350 (3)	0.5507 (10)	0.59009 (12)	0.0786 (12)	
H65A	0.4455	0.4943	0.6162	0.094*	
C66	0.4716 (3)	0.7560 (10)	0.58280 (15)	0.0811 (13)	
H66A	0.5074	0.8343	0.6042	0.097*	
C67	0.4561 (2)	0.8437 (7)	0.54536 (14)	0.0688 (10)	
H67A	0.4794	0.9834	0.5414	0.083*	
C68	0.4040 (2)	0.7234 (6)	0.51177 (12)	0.0561 (8)	
C69	0.3902 (2)	0.8050 (6)	0.47211 (12)	0.0608 (8)	
H69A	0.4123	0.9450	0.4675	0.073*	
C70	0.3452 (2)	0.6821 (6)	0.44050 (11)	0.0587 (8)	
H70A	0.3370	0.7382	0.4145	0.070*	
C71	0.2688 (2)	0.3262 (7)	0.41287 (11)	0.0579 (8)	
H71A	0.2441	0.1902	0.4181	0.069*	
C72	0.2241 (3)	0.2199 (10)	0.34534 (12)	0.0836 (13)	
H72A	0.2053	0.0878	0.3584	0.100*	
C73	0.1402 (4)	0.3254 (17)	0.31912 (17)	0.121 (2)	
H73A	0.1123	0.2225	0.2985	0.182*	
H73B	0.0972	0.3630	0.3353	0.182*	
H73C	0.1578	0.4577	0.3069	0.182*	
C74	0.2959 (4)	0.1528 (9)	0.32225 (12)	0.0798 (11)	
C75	0.3347 (5)	−0.0576 (13)	0.32531 (17)	0.1092 (19)	
H75A	0.3136	−0.1648	0.3408	0.131*	
C76	0.4044 (6)	−0.1128 (17)	0.3057 (3)	0.132 (3)	
H76A	0.4299	−0.2546	0.3087	0.158*	
C77	0.4352 (6)	0.0374 (18)	0.2825 (2)	0.131 (3)	
H77A	0.4807	0.0001	0.2689	0.157*	
C78	0.3987 (8)	0.2431 (19)	0.2795 (3)	0.151 (4)	
H78A	0.4192	0.3484	0.2634	0.181*	
C79	0.3318 (7)	0.3004 (16)	0.2996 (2)	0.136 (3)	
H79A	0.3102	0.4459	0.2977	0.164*	

### (1) (S)-(+)-2-{[(1-Phenylethyl)imino]methyl}naphthalene Atomic displacement parameters (Å^2^)

**Table:**

	*U*^11^	*U*^22^	*U*^33^	*U*^12^	*U*^13^	*U*^23^
N1	0.0601 (15)	0.077 (2)	0.0677 (17)	−0.0013 (15)	0.0091 (13)	−0.0007 (16)
C1	0.0347 (12)	0.0521 (18)	0.0677 (18)	−0.0048 (12)	0.0099 (12)	0.0009 (15)
C2	0.0362 (12)	0.0388 (15)	0.0698 (17)	0.0004 (12)	0.0072 (12)	−0.0024 (14)
C3	0.0333 (12)	0.0418 (16)	0.0692 (17)	−0.0062 (11)	0.0094 (11)	−0.0010 (14)
C4	0.0510 (15)	0.052 (2)	0.0708 (19)	−0.0034 (15)	0.0091 (14)	0.0035 (16)
C5	0.0639 (19)	0.080 (3)	0.0636 (18)	−0.0076 (19)	0.0139 (15)	−0.0013 (18)
C6	0.0554 (17)	0.084 (3)	0.081 (2)	−0.0093 (19)	0.0214 (16)	−0.022 (2)
C7	0.0431 (14)	0.058 (2)	0.096 (3)	0.0009 (14)	0.0172 (14)	−0.0174 (19)
C8	0.0314 (11)	0.0428 (16)	0.0807 (19)	−0.0057 (11)	0.0109 (11)	−0.0042 (15)
C9	0.0411 (13)	0.0373 (16)	0.091 (2)	0.0020 (12)	0.0097 (13)	0.0071 (16)
C10	0.0430 (13)	0.0550 (19)	0.0700 (17)	−0.0026 (13)	0.0073 (12)	0.0120 (16)
C11	0.0434 (13)	0.064 (2)	0.0633 (17)	−0.0009 (14)	0.0092 (12)	−0.0011 (16)
C12	0.070 (2)	0.107 (4)	0.066 (2)	0.015 (2)	0.0116 (16)	−0.010 (2)
C13	0.072 (2)	0.218 (9)	0.092 (3)	−0.019 (4)	0.031 (2)	−0.021 (5)
C14	0.0732 (19)	0.081 (3)	0.0526 (15)	0.0040 (19)	0.0188 (14)	−0.0093 (17)
C15	0.077 (2)	0.087 (3)	0.074 (2)	0.008 (2)	0.0121 (18)	−0.006 (2)
C16	0.085 (3)	0.140 (6)	0.081 (3)	0.026 (4)	0.002 (2)	−0.019 (3)
C17	0.086 (3)	0.138 (6)	0.107 (4)	−0.006 (4)	0.014 (3)	−0.051 (4)
C18	0.121 (5)	0.111 (5)	0.123 (4)	−0.043 (4)	0.039 (4)	−0.039 (4)
C19	0.111 (3)	0.086 (3)	0.080 (3)	−0.005 (3)	0.026 (2)	−0.006 (2)
N2	0.0652 (15)	0.0618 (19)	0.0551 (13)	0.0062 (14)	0.0121 (11)	0.0011 (13)
C21	0.0358 (11)	0.0485 (17)	0.0584 (15)	−0.0045 (12)	0.0096 (11)	−0.0025 (14)
C22	0.0382 (12)	0.0427 (15)	0.0620 (16)	0.0049 (12)	0.0139 (11)	−0.0005 (13)
C23	0.0336 (11)	0.0522 (18)	0.0564 (15)	−0.0022 (11)	0.0102 (11)	−0.0021 (13)
C24	0.0537 (15)	0.059 (2)	0.0645 (17)	0.0071 (15)	0.0160 (13)	0.0081 (16)
C25	0.070 (2)	0.088 (3)	0.0585 (17)	0.006 (2)	0.0205 (15)	0.0104 (19)
C26	0.071 (2)	0.088 (3)	0.0589 (18)	0.004 (2)	0.0230 (15)	−0.0137 (19)
C27	0.0509 (15)	0.063 (2)	0.0704 (19)	0.0080 (16)	0.0159 (14)	−0.0114 (18)
C28	0.0344 (11)	0.0513 (18)	0.0617 (15)	−0.0037 (12)	0.0103 (11)	−0.0103 (14)
C29	0.0395 (12)	0.0425 (16)	0.0752 (18)	0.0043 (12)	0.0107 (12)	−0.0061 (14)
C30	0.0420 (12)	0.0513 (18)	0.0615 (15)	−0.0025 (13)	0.0067 (11)	0.0039 (14)
C31	0.0452 (13)	0.0489 (18)	0.0577 (16)	0.0015 (12)	0.0120 (12)	−0.0003 (14)
C32	0.0684 (18)	0.073 (2)	0.0526 (15)	0.0123 (17)	0.0146 (14)	0.0010 (16)
C33	0.067 (2)	0.118 (4)	0.069 (2)	0.001 (2)	0.0188 (16)	0.005 (2)
C34	0.0719 (18)	0.066 (2)	0.0499 (14)	0.0010 (16)	0.0222 (13)	0.0059 (14)
C35	0.100 (3)	0.078 (3)	0.070 (2)	0.002 (2)	0.020 (2)	−0.006 (2)
C36	0.118 (4)	0.102 (4)	0.073 (2)	−0.027 (3)	0.018 (3)	−0.019 (3)
C37	0.086 (3)	0.129 (5)	0.067 (2)	−0.033 (3)	0.0081 (19)	0.006 (3)
C38	0.0641 (19)	0.108 (4)	0.074 (2)	0.001 (2)	0.0160 (17)	0.018 (2)
C39	0.0649 (17)	0.076 (2)	0.0631 (17)	−0.0012 (18)	0.0197 (14)	0.0042 (17)
N3	0.0677 (16)	0.071 (2)	0.0580 (15)	−0.0017 (15)	0.0175 (12)	−0.0013 (14)
C41	0.0413 (13)	0.0545 (18)	0.0588 (16)	0.0012 (13)	0.0166 (11)	−0.0020 (14)
C42	0.0425 (13)	0.0440 (15)	0.0646 (16)	−0.0014 (12)	0.0159 (12)	−0.0032 (14)
C43	0.0388 (13)	0.0463 (17)	0.0631 (17)	0.0032 (12)	0.0157 (12)	0.0002 (14)
C44	0.0535 (15)	0.0554 (19)	0.0695 (19)	0.0018 (14)	0.0166 (13)	0.0063 (16)
C45	0.073 (2)	0.087 (3)	0.0613 (18)	0.003 (2)	0.0094 (16)	0.014 (2)
C46	0.0618 (18)	0.093 (3)	0.0635 (18)	−0.001 (2)	0.0047 (15)	−0.004 (2)
C47	0.0519 (16)	0.061 (2)	0.072 (2)	−0.0042 (16)	0.0135 (14)	−0.0123 (17)
C48	0.0424 (13)	0.0504 (17)	0.0641 (16)	0.0028 (12)	0.0189 (11)	−0.0064 (14)
C49	0.0507 (14)	0.0435 (16)	0.0741 (19)	−0.0032 (13)	0.0240 (14)	−0.0026 (15)
C50	0.0549 (15)	0.0520 (18)	0.0598 (16)	0.0016 (14)	0.0226 (13)	0.0060 (14)
C51	0.0457 (14)	0.062 (2)	0.0612 (17)	−0.0011 (14)	0.0164 (13)	−0.0012 (16)
C52	0.070 (2)	0.088 (3)	0.0584 (17)	−0.012 (2)	0.0151 (15)	−0.0020 (19)
C53	0.069 (2)	0.181 (7)	0.073 (2)	0.013 (3)	0.0195 (18)	0.002 (3)
C54	0.0586 (16)	0.076 (2)	0.0503 (14)	−0.0027 (16)	0.0062 (12)	0.0018 (15)
C55	0.081 (2)	0.090 (3)	0.073 (2)	−0.014 (2)	0.0142 (18)	−0.011 (2)
C56	0.089 (3)	0.119 (5)	0.073 (2)	−0.005 (3)	0.021 (2)	−0.031 (3)
C57	0.067 (2)	0.154 (5)	0.0576 (18)	0.002 (3)	0.0134 (16)	0.001 (3)
C58	0.083 (2)	0.122 (4)	0.071 (2)	−0.029 (3)	0.0226 (18)	0.003 (3)
C59	0.082 (2)	0.088 (3)	0.073 (2)	−0.015 (2)	0.0221 (18)	−0.007 (2)
N4	0.0760 (18)	0.070 (2)	0.0617 (16)	−0.0039 (16)	0.0165 (14)	0.0041 (15)
C61	0.0439 (13)	0.0472 (17)	0.0668 (18)	0.0027 (13)	0.0185 (13)	0.0023 (14)
C62	0.0434 (13)	0.0412 (15)	0.0704 (18)	0.0001 (12)	0.0194 (13)	0.0018 (14)
C63	0.0406 (13)	0.0502 (19)	0.0692 (17)	0.0036 (13)	0.0208 (12)	0.0000 (14)
C64	0.0589 (17)	0.062 (2)	0.069 (2)	−0.0008 (16)	0.0190 (15)	−0.0039 (17)
C65	0.077 (2)	0.096 (3)	0.0624 (19)	0.009 (2)	0.0164 (17)	−0.007 (2)
C66	0.0578 (19)	0.097 (4)	0.089 (3)	−0.009 (2)	0.0176 (18)	−0.034 (3)
C67	0.0538 (16)	0.062 (2)	0.095 (3)	−0.0091 (16)	0.0258 (17)	−0.019 (2)
C68	0.0392 (13)	0.0497 (19)	0.084 (2)	0.0008 (13)	0.0229 (13)	−0.0095 (16)
C69	0.0547 (16)	0.0414 (18)	0.092 (2)	0.0024 (14)	0.0279 (16)	0.0087 (17)
C70	0.0533 (15)	0.055 (2)	0.0707 (19)	0.0037 (15)	0.0203 (13)	0.0101 (16)
C71	0.0479 (15)	0.059 (2)	0.0685 (19)	−0.0018 (14)	0.0158 (13)	0.0034 (17)
C72	0.090 (3)	0.101 (4)	0.0569 (18)	−0.022 (3)	0.0116 (18)	−0.005 (2)
C73	0.095 (3)	0.177 (7)	0.082 (3)	0.009 (4)	−0.001 (3)	−0.005 (4)
C74	0.098 (3)	0.082 (3)	0.0578 (18)	−0.015 (2)	0.0135 (18)	−0.0021 (19)
C75	0.122 (4)	0.119 (5)	0.083 (3)	0.001 (4)	0.016 (3)	0.018 (3)
C76	0.121 (5)	0.137 (7)	0.135 (5)	0.028 (5)	0.026 (4)	−0.011 (5)
C77	0.137 (5)	0.166 (8)	0.103 (4)	−0.005 (5)	0.053 (4)	−0.029 (5)
C78	0.185 (8)	0.155 (8)	0.141 (6)	0.006 (7)	0.098 (7)	0.022 (6)
C79	0.171 (7)	0.122 (6)	0.142 (6)	0.011 (5)	0.091 (6)	0.031 (5)

### (1) (S)-(+)-2-{[(1-Phenylethyl)imino]methyl}naphthalene Geometric parameters (Å, º)

**Table:**

N1—C11	1.265 (5)	N3—C51	1.260 (5)
N1—C12	1.472 (6)	N3—C52	1.465 (5)
C1—C2	1.366 (5)	C41—C42	1.368 (5)
C1—C10	1.415 (5)	C41—C50	1.415 (5)
C1—C11	1.481 (5)	C41—C51	1.470 (5)
C2—C3	1.414 (5)	C42—C43	1.417 (5)
C2—H2A	0.9300	C42—H42A	0.9300
C3—C4	1.407 (5)	C43—C44	1.409 (5)
C3—C8	1.430 (5)	C43—C48	1.420 (5)
C4—C5	1.355 (6)	C44—C45	1.356 (6)
C4—H4A	0.9300	C44—H44A	0.9300
C5—C6	1.408 (7)	C45—C46	1.399 (7)
C5—H5A	0.9300	C45—H45A	0.9300
C6—C7	1.364 (7)	C46—C47	1.370 (6)
C6—H6A	0.9300	C46—H46A	0.9300
C7—C8	1.401 (5)	C47—C48	1.418 (5)
C7—H7A	0.9300	C47—H47A	0.9300
C8—C9	1.414 (5)	C48—C49	1.411 (5)
C9—C10	1.361 (6)	C49—C50	1.365 (5)
C9—H9A	0.9300	C49—H49A	0.9300
C10—H10A	0.9300	C50—H50A	0.9300
C11—H11A	0.9300	C51—H51A	0.9300
C12—C14	1.506 (6)	C52—C54	1.523 (5)
C12—C13	1.535 (8)	C52—C53	1.528 (8)
C12—H12A	0.9800	C52—H52A	0.9800
C13—H13A	0.9600	C53—H53A	0.9600
C13—H13B	0.9600	C53—H53B	0.9600
C13—H13C	0.9600	C53—H53C	0.9600
C14—C15	1.370 (7)	C54—C55	1.362 (6)
C14—C19	1.370 (7)	C54—C59	1.384 (6)
C15—C16	1.369 (8)	C55—C56	1.386 (7)
C15—H15A	0.9300	C55—H55A	0.9300
C16—C17	1.358 (11)	C56—C57	1.361 (9)
C16—H16A	0.9300	C56—H56A	0.9300
C17—C18	1.352 (11)	C57—C58	1.359 (9)
C17—H17A	0.9300	C57—H57A	0.9300
C18—C19	1.401 (9)	C58—C59	1.375 (6)
C18—H18A	0.9300	C58—H58A	0.9300
C19—H19A	0.9300	C59—H59A	0.9300
N2—C31	1.255 (5)	N4—C71	1.268 (5)
N2—C32	1.476 (5)	N4—C72	1.464 (6)
C21—C22	1.371 (5)	C61—C62	1.370 (5)
C21—C30	1.405 (5)	C61—C70	1.425 (5)
C21—C31	1.475 (4)	C61—C71	1.459 (5)
C22—C23	1.423 (4)	C62—C63	1.417 (5)
C22—H22A	0.9300	C62—H62A	0.9300
C23—C28	1.413 (5)	C63—C64	1.408 (5)
C23—C24	1.415 (5)	C63—C68	1.410 (5)
C24—C25	1.368 (5)	C64—C65	1.373 (6)
C24—H24A	0.9300	C64—H64A	0.9300
C25—C26	1.400 (7)	C65—C66	1.393 (8)
C25—H25A	0.9300	C65—H65A	0.9300
C26—C27	1.357 (6)	C66—C67	1.350 (7)
C26—H26A	0.9300	C66—H66A	0.9300
C27—C28	1.420 (5)	C67—C68	1.429 (6)
C27—H27A	0.9300	C67—H67A	0.9300
C28—C29	1.413 (5)	C68—C69	1.407 (6)
C29—C30	1.367 (5)	C69—C70	1.356 (6)
C29—H29A	0.9300	C69—H69A	0.9300
C30—H30A	0.9300	C70—H70A	0.9300
C31—H31A	0.9300	C71—H71A	0.9300
C32—C34	1.502 (5)	C72—C73	1.510 (8)
C32—C33	1.525 (6)	C72—C74	1.515 (7)
C32—H32A	0.9800	C72—H72A	0.9800
C33—H33A	0.9600	C73—H73A	0.9600
C33—H33B	0.9600	C73—H73B	0.9600
C33—H33C	0.9600	C73—H73C	0.9600
C34—C39	1.374 (6)	C74—C79	1.359 (9)
C34—C35	1.383 (6)	C74—C75	1.385 (9)
C35—C36	1.388 (8)	C75—C76	1.394 (11)
C35—H35A	0.9300	C75—H75A	0.9300
C36—C37	1.363 (10)	C76—C77	1.344 (12)
C36—H36A	0.9300	C76—H76A	0.9300
C37—C38	1.379 (8)	C77—C78	1.346 (13)
C37—H37A	0.9300	C77—H77A	0.9300
C38—C39	1.391 (6)	C78—C79	1.373 (11)
C38—H38A	0.9300	C78—H78A	0.9300
C39—H39A	0.9300	C79—H79A	0.9300
			
C11—N1—C12	116.5 (4)	C51—N3—C52	117.7 (4)
C2—C1—C10	120.0 (3)	C42—C41—C50	119.4 (3)
C2—C1—C11	119.1 (3)	C42—C41—C51	119.5 (3)
C10—C1—C11	120.7 (3)	C50—C41—C51	121.1 (3)
C1—C2—C3	121.3 (3)	C41—C42—C43	121.5 (3)
C1—C2—H2A	119.4	C41—C42—H42A	119.2
C3—C2—H2A	119.4	C43—C42—H42A	119.2
C4—C3—C2	123.0 (3)	C44—C43—C42	122.1 (3)
C4—C3—C8	118.4 (3)	C44—C43—C48	119.3 (3)
C2—C3—C8	118.6 (3)	C42—C43—C48	118.6 (3)
C5—C4—C3	121.3 (4)	C45—C44—C43	120.5 (4)
C5—C4—H4A	119.3	C45—C44—H44A	119.7
C3—C4—H4A	119.3	C43—C44—H44A	119.7
C4—C5—C6	120.4 (4)	C44—C45—C46	120.8 (4)
C4—C5—H5A	119.8	C44—C45—H45A	119.6
C6—C5—H5A	119.8	C46—C45—H45A	119.6
C7—C6—C5	119.8 (4)	C47—C46—C45	120.5 (4)
C7—C6—H6A	120.1	C47—C46—H46A	119.7
C5—C6—H6A	120.1	C45—C46—H46A	119.7
C6—C7—C8	121.4 (4)	C46—C47—C48	120.2 (4)
C6—C7—H7A	119.3	C46—C47—H47A	119.9
C8—C7—H7A	119.3	C48—C47—H47A	119.9
C7—C8—C9	122.9 (3)	C49—C48—C47	122.4 (3)
C7—C8—C3	118.6 (3)	C49—C48—C43	118.9 (3)
C9—C8—C3	118.5 (3)	C47—C48—C43	118.6 (3)
C10—C9—C8	121.5 (3)	C50—C49—C48	121.0 (3)
C10—C9—H9A	119.3	C50—C49—H49A	119.5
C8—C9—H9A	119.3	C48—C49—H49A	119.5
C9—C10—C1	120.1 (3)	C49—C50—C41	120.5 (3)
C9—C10—H10A	120.0	C49—C50—H50A	119.7
C1—C10—H10A	120.0	C41—C50—H50A	119.7
N1—C11—C1	121.7 (4)	N3—C51—C41	122.4 (3)
N1—C11—H11A	119.1	N3—C51—H51A	118.8
C1—C11—H11A	119.1	C41—C51—H51A	118.8
N1—C12—C14	107.6 (3)	N3—C52—C54	109.3 (3)
N1—C12—C13	107.6 (5)	N3—C52—C53	108.0 (4)
C14—C12—C13	113.1 (4)	C54—C52—C53	110.3 (3)
N1—C12—H12A	109.5	N3—C52—H52A	109.8
C14—C12—H12A	109.5	C54—C52—H52A	109.8
C13—C12—H12A	109.5	C53—C52—H52A	109.8
C12—C13—H13A	109.5	C52—C53—H53A	109.5
C12—C13—H13B	109.5	C52—C53—H53B	109.5
H13A—C13—H13B	109.5	H53A—C53—H53B	109.5
C12—C13—H13C	109.5	C52—C53—H53C	109.5
H13A—C13—H13C	109.5	H53A—C53—H53C	109.5
H13B—C13—H13C	109.5	H53B—C53—H53C	109.5
C15—C14—C19	116.9 (5)	C55—C54—C59	117.6 (4)
C15—C14—C12	121.6 (5)	C55—C54—C52	121.3 (4)
C19—C14—C12	121.4 (5)	C59—C54—C52	121.1 (4)
C16—C15—C14	123.1 (6)	C54—C55—C56	121.1 (5)
C16—C15—H15A	118.5	C54—C55—H55A	119.4
C14—C15—H15A	118.5	C56—C55—H55A	119.4
C17—C16—C15	119.2 (6)	C57—C56—C55	120.8 (5)
C17—C16—H16A	120.4	C57—C56—H56A	119.6
C15—C16—H16A	120.4	C55—C56—H56A	119.6
C18—C17—C16	119.9 (6)	C58—C57—C56	118.6 (4)
C18—C17—H17A	120.0	C58—C57—H57A	120.7
C16—C17—H17A	120.0	C56—C57—H57A	120.7
C17—C18—C19	120.4 (6)	C57—C58—C59	121.0 (5)
C17—C18—H18A	119.8	C57—C58—H58A	119.5
C19—C18—H18A	119.8	C59—C58—H58A	119.5
C14—C19—C18	120.5 (6)	C58—C59—C54	120.9 (5)
C14—C19—H19A	119.8	C58—C59—H59A	119.6
C18—C19—H19A	119.8	C54—C59—H59A	119.6
C31—N2—C32	117.1 (3)	C71—N4—C72	117.3 (4)
C22—C21—C30	119.8 (3)	C62—C61—C70	119.1 (3)
C22—C21—C31	118.5 (3)	C62—C61—C71	119.9 (3)
C30—C21—C31	121.7 (3)	C70—C61—C71	120.9 (3)
C21—C22—C23	121.1 (3)	C61—C62—C63	121.5 (3)
C21—C22—H22A	119.4	C61—C62—H62A	119.3
C23—C22—H22A	119.4	C63—C62—H62A	119.3
C28—C23—C24	119.4 (3)	C64—C63—C68	119.3 (4)
C28—C23—C22	118.8 (3)	C64—C63—C62	122.3 (4)
C24—C23—C22	121.8 (3)	C68—C63—C62	118.3 (3)
C25—C24—C23	120.1 (4)	C65—C64—C63	120.7 (4)
C25—C24—H24A	119.9	C65—C64—H64A	119.7
C23—C24—H24A	119.9	C63—C64—H64A	119.7
C24—C25—C26	120.6 (4)	C64—C65—C66	119.8 (4)
C24—C25—H25A	119.7	C64—C65—H65A	120.1
C26—C25—H25A	119.7	C66—C65—H65A	120.1
C27—C26—C25	120.5 (3)	C67—C66—C65	121.3 (4)
C27—C26—H26A	119.7	C67—C66—H66A	119.3
C25—C26—H26A	119.7	C65—C66—H66A	119.3
C26—C27—C28	120.9 (4)	C66—C67—C68	120.5 (4)
C26—C27—H27A	119.6	C66—C67—H67A	119.8
C28—C27—H27A	119.6	C68—C67—H67A	119.8
C29—C28—C23	118.5 (3)	C69—C68—C63	119.7 (3)
C29—C28—C27	123.0 (3)	C69—C68—C67	122.0 (4)
C23—C28—C27	118.4 (3)	C63—C68—C67	118.3 (4)
C30—C29—C28	121.6 (3)	C70—C69—C68	120.9 (3)
C30—C29—H29A	119.2	C70—C69—H69A	119.6
C28—C29—H29A	119.2	C68—C69—H69A	119.6
C29—C30—C21	120.2 (3)	C69—C70—C61	120.5 (3)
C29—C30—H30A	119.9	C69—C70—H70A	119.7
C21—C30—H30A	119.9	C61—C70—H70A	119.7
N2—C31—C21	122.8 (3)	N4—C71—C61	122.9 (4)
N2—C31—H31A	118.6	N4—C71—H71A	118.6
C21—C31—H31A	118.6	C61—C71—H71A	118.6
N2—C32—C34	110.2 (3)	N4—C72—C73	108.2 (5)
N2—C32—C33	108.1 (4)	N4—C72—C74	108.6 (4)
C34—C32—C33	111.2 (3)	C73—C72—C74	113.3 (4)
N2—C32—H32A	109.1	N4—C72—H72A	108.9
C34—C32—H32A	109.1	C73—C72—H72A	108.9
C33—C32—H32A	109.1	C74—C72—H72A	108.9
C32—C33—H33A	109.5	C72—C73—H73A	109.5
C32—C33—H33B	109.5	C72—C73—H73B	109.5
H33A—C33—H33B	109.5	H73A—C73—H73B	109.5
C32—C33—H33C	109.5	C72—C73—H73C	109.5
H33A—C33—H33C	109.5	H73A—C73—H73C	109.5
H33B—C33—H33C	109.5	H73B—C73—H73C	109.5
C39—C34—C35	118.6 (4)	C79—C74—C75	115.4 (6)
C39—C34—C32	122.0 (4)	C79—C74—C72	122.1 (6)
C35—C34—C32	119.3 (4)	C75—C74—C72	122.4 (5)
C34—C35—C36	120.6 (5)	C74—C75—C76	121.7 (7)
C34—C35—H35A	119.7	C74—C75—H75A	119.2
C36—C35—H35A	119.7	C76—C75—H75A	119.2
C37—C36—C35	120.5 (5)	C77—C76—C75	120.5 (8)
C37—C36—H36A	119.8	C77—C76—H76A	119.8
C35—C36—H36A	119.8	C75—C76—H76A	119.8
C36—C37—C38	119.4 (5)	C76—C77—C78	118.6 (7)
C36—C37—H37A	120.3	C76—C77—H77A	120.7
C38—C37—H37A	120.3	C78—C77—H77A	120.7
C37—C38—C39	120.2 (5)	C77—C78—C79	121.2 (8)
C37—C38—H38A	119.9	C77—C78—H78A	119.4
C39—C38—H38A	119.9	C79—C78—H78A	119.4
C34—C39—C38	120.6 (4)	C74—C79—C78	122.6 (9)
C34—C39—H39A	119.7	C74—C79—H79A	118.7
C38—C39—H39A	119.7	C78—C79—H79A	118.7
			
C10—C1—C2—C3	−2.4 (4)	C50—C41—C42—C43	−1.2 (5)
C11—C1—C2—C3	173.9 (3)	C51—C41—C42—C43	176.6 (3)
C1—C2—C3—C4	−177.2 (3)	C41—C42—C43—C44	−178.3 (3)
C1—C2—C3—C8	0.6 (4)	C41—C42—C43—C48	0.3 (4)
C2—C3—C4—C5	175.4 (3)	C42—C43—C44—C45	177.6 (3)
C8—C3—C4—C5	−2.4 (5)	C48—C43—C44—C45	−1.0 (5)
C3—C4—C5—C6	1.2 (6)	C43—C44—C45—C46	0.0 (6)
C4—C5—C6—C7	1.1 (6)	C44—C45—C46—C47	1.4 (7)
C5—C6—C7—C8	−2.1 (6)	C45—C46—C47—C48	−1.6 (6)
C6—C7—C8—C9	−177.2 (3)	C46—C47—C48—C49	−178.4 (3)
C6—C7—C8—C3	0.8 (5)	C46—C47—C48—C43	0.5 (5)
C4—C3—C8—C7	1.4 (4)	C44—C43—C48—C49	179.7 (3)
C2—C3—C8—C7	−176.4 (3)	C42—C43—C48—C49	1.1 (4)
C4—C3—C8—C9	179.5 (3)	C44—C43—C48—C47	0.8 (4)
C2—C3—C8—C9	1.7 (4)	C42—C43—C48—C47	−177.9 (3)
C7—C8—C9—C10	175.8 (3)	C47—C48—C49—C50	177.4 (3)
C3—C8—C9—C10	−2.2 (4)	C43—C48—C49—C50	−1.6 (5)
C8—C9—C10—C1	0.4 (4)	C48—C49—C50—C41	0.6 (5)
C2—C1—C10—C9	1.9 (4)	C42—C41—C50—C49	0.8 (5)
C11—C1—C10—C9	−174.3 (3)	C51—C41—C50—C49	−177.0 (3)
C12—N1—C11—C1	175.0 (3)	C52—N3—C51—C41	178.1 (3)
C2—C1—C11—N1	−174.5 (3)	C42—C41—C51—N3	179.9 (3)
C10—C1—C11—N1	1.7 (5)	C50—C41—C51—N3	−2.4 (5)
C11—N1—C12—C14	−118.7 (4)	C51—N3—C52—C54	−123.3 (4)
C11—N1—C12—C13	119.0 (5)	C51—N3—C52—C53	116.7 (4)
N1—C12—C14—C15	−67.2 (5)	N3—C52—C54—C55	142.0 (4)
C13—C12—C14—C15	51.5 (7)	C53—C52—C54—C55	−99.5 (6)
N1—C12—C14—C19	110.0 (5)	N3—C52—C54—C59	−40.0 (6)
C13—C12—C14—C19	−131.3 (6)	C53—C52—C54—C59	78.6 (5)
C19—C14—C15—C16	0.8 (7)	C59—C54—C55—C56	0.3 (7)
C12—C14—C15—C16	178.1 (4)	C52—C54—C55—C56	178.5 (5)
C14—C15—C16—C17	−1.1 (8)	C54—C55—C56—C57	−1.0 (8)
C15—C16—C17—C18	0.7 (9)	C55—C56—C57—C58	0.9 (8)
C16—C17—C18—C19	−0.2 (9)	C56—C57—C58—C59	−0.2 (8)
C15—C14—C19—C18	−0.2 (7)	C57—C58—C59—C54	−0.5 (8)
C12—C14—C19—C18	−177.5 (5)	C55—C54—C59—C58	0.4 (7)
C17—C18—C19—C14	−0.1 (9)	C52—C54—C59—C58	−177.8 (5)
C30—C21—C22—C23	−1.3 (4)	C70—C61—C62—C63	−1.6 (5)
C31—C21—C22—C23	177.3 (3)	C71—C61—C62—C63	174.3 (3)
C21—C22—C23—C28	0.6 (4)	C61—C62—C63—C64	−177.3 (3)
C21—C22—C23—C24	−178.0 (3)	C61—C62—C63—C68	0.2 (4)
C28—C23—C24—C25	−0.5 (5)	C68—C63—C64—C65	−2.2 (5)
C22—C23—C24—C25	178.0 (3)	C62—C63—C64—C65	175.4 (3)
C23—C24—C25—C26	−0.3 (6)	C63—C64—C65—C66	1.1 (6)
C24—C25—C26—C27	0.8 (6)	C64—C65—C66—C67	1.2 (7)
C25—C26—C27—C28	−0.3 (6)	C65—C66—C67—C68	−2.3 (6)
C24—C23—C28—C29	179.4 (3)	C64—C63—C68—C69	179.2 (3)
C22—C23—C28—C29	0.8 (4)	C62—C63—C68—C69	1.6 (4)
C24—C23—C28—C27	1.0 (4)	C64—C63—C68—C67	1.0 (4)
C22—C23—C28—C27	−177.6 (3)	C62—C63—C68—C67	−176.6 (3)
C26—C27—C28—C29	−178.9 (3)	C66—C67—C68—C69	−176.9 (3)
C26—C27—C28—C23	−0.6 (5)	C66—C67—C68—C63	1.1 (5)
C23—C28—C29—C30	−1.5 (4)	C63—C68—C69—C70	−1.9 (5)
C27—C28—C29—C30	176.9 (3)	C67—C68—C69—C70	176.2 (3)
C28—C29—C30—C21	0.7 (5)	C68—C69—C70—C61	0.4 (5)
C22—C21—C30—C29	0.6 (4)	C62—C61—C70—C69	1.3 (5)
C31—C21—C30—C29	−177.9 (3)	C71—C61—C70—C69	−174.5 (3)
C32—N2—C31—C21	179.3 (3)	C72—N4—C71—C61	178.3 (4)
C22—C21—C31—N2	−177.8 (3)	C62—C61—C71—N4	−172.8 (4)
C30—C21—C31—N2	0.8 (5)	C70—C61—C71—N4	3.0 (5)
C31—N2—C32—C34	−118.9 (4)	C71—N4—C72—C73	117.8 (5)
C31—N2—C32—C33	119.5 (4)	C71—N4—C72—C74	−118.8 (4)
N2—C32—C34—C39	−41.5 (5)	N4—C72—C74—C79	−64.6 (8)
C33—C32—C34—C39	78.3 (4)	C73—C72—C74—C79	55.6 (9)
N2—C32—C34—C35	140.7 (4)	N4—C72—C74—C75	110.0 (6)
C33—C32—C34—C35	−99.5 (5)	C73—C72—C74—C75	−129.7 (7)
C39—C34—C35—C36	0.7 (6)	C79—C74—C75—C76	−1.2 (10)
C32—C34—C35—C36	178.5 (4)	C72—C74—C75—C76	−176.2 (6)
C34—C35—C36—C37	−0.6 (8)	C74—C75—C76—C77	−1.1 (12)
C35—C36—C37—C38	0.5 (8)	C75—C76—C77—C78	1.6 (14)
C36—C37—C38—C39	−0.4 (7)	C76—C77—C78—C79	0.3 (17)
C35—C34—C39—C38	−0.6 (5)	C75—C74—C79—C78	3.1 (13)
C32—C34—C39—C38	−178.4 (3)	C72—C74—C79—C78	178.1 (9)
C37—C38—C39—C34	0.4 (6)	C77—C78—C79—C74	−2.8 (18)

### (2) (S)-(+)-2-({[(4-Methylphenyl)ethyl]imino}methyl)naphthalene Crystal data

**Table:**

C_20_H_19_N	*D*_x_ = 1.155 Mg m^−^^3^
*M**_r_* = 273.36	Melting point: 397 K
Orthorhombic, *P*2_1_2_1_2_1_	Cu *K*α radiation, λ = 1.54184 Å
*a* = 6.0946 (5) Å	Cell parameters from 2035 reflections
*b* = 7.5732 (5) Å	θ = 5.2–68.7°
*c* = 34.046 (3) Å	µ = 0.51 mm^−^^1^
*V* = 1571.4 (2) Å^3^	*T* = 298 K
*Z* = 4	Prism, colorless
*F*(000) = 584	0.49 × 0.13 × 0.05 mm

### (2) (S)-(+)-2-({[(4-Methylphenyl)ethyl]imino}methyl)naphthalene Data collection

**Table:**

Agilent Xcalibur Atlas Gemini diffractometer	2770 independent reflections
Radiation source: Enhance (Cu) X-ray Source	2048 reflections with *I* > 2σ(*I*)
Graphite monochromator	*R*_int_ = 0.041
Detector resolution: 10.5564 pixels mm^-1^	θ_max_ = 66.6°, θ_min_ = 5.2°
ω scans	*h* = −6→7
Absorption correction: analytical (CrysAlis PRO; Agilent, 2013)	*k* = −9→9
*T*_min_ = 0.793, *T*_max_ = 0.969	*l* = −40→40
13738 measured reflections	

### (2) (S)-(+)-2-({[(4-Methylphenyl)ethyl]imino}methyl)naphthalene Refinement

**Table:**

Refinement on *F*^2^	Primary atom site location: structure-invariant direct methods
Least-squares matrix: full	Secondary atom site location: difference Fourier map
*R*[*F*^2^ > 2σ(*F*^2^)] = 0.044	Hydrogen site location: inferred from neighbouring sites
*wR*(*F*^2^) = 0.112	H-atom parameters constrained
*S* = 1.08	*w* = 1/[σ^2^(*F*_o_^2^) + (0.0467*P*)^2^ + 0.0773*P*] where *P* = (*F*_o_^2^ + 2*F*_c_^2^)/3
2770 reflections	(Δ/σ)_max_ = 0.001
192 parameters	Δρ_max_ = 0.09 e Å^−^^3^
0 restraints	Δρ_min_ = −0.11 e Å^−^^3^
0 constraints	

### (2) (S)-(+)-2-({[(4-Methylphenyl)ethyl]imino}methyl)naphthalene Fractional atomic coordinates and isotropic or equivalent isotropic displacement parameters (Å^2^)

**Table:**

	*x*	*y*	*z*	*U*_iso_*/*U*_eq_	
N1	0.9455 (5)	0.3422 (3)	0.88634 (7)	0.0756 (8)	
C1	0.9081 (5)	0.4073 (3)	0.95510 (8)	0.0555 (7)	
C2	1.0099 (5)	0.4065 (3)	0.99087 (8)	0.0548 (7)	
H2A	1.1462	0.3524	0.9933	0.066*	
C3	0.9129 (5)	0.4860 (3)	1.02429 (7)	0.0533 (7)	
C4	1.0188 (5)	0.4938 (4)	1.06118 (8)	0.0657 (8)	
H4A	1.1550	0.4403	1.0644	0.079*	
C5	0.9246 (6)	0.5786 (4)	1.09209 (9)	0.0762 (9)	
H5A	0.9983	0.5846	1.1160	0.091*	
C6	0.7180 (7)	0.6567 (4)	1.08826 (10)	0.0796 (10)	
H6A	0.6546	0.7140	1.1096	0.095*	
C7	0.6094 (6)	0.6492 (4)	1.05335 (10)	0.0704 (9)	
H7A	0.4716	0.7009	1.0511	0.084*	
C8	0.7030 (5)	0.5642 (3)	1.02042 (8)	0.0572 (7)	
C9	0.5983 (5)	0.5589 (4)	0.98321 (9)	0.0631 (7)	
H9A	0.4590	0.6074	0.9804	0.076*	
C10	0.6976 (5)	0.4846 (4)	0.95165 (8)	0.0616 (8)	
H10A	0.6266	0.4844	0.9275	0.074*	
C11	1.0219 (6)	0.3337 (4)	0.92099 (8)	0.0638 (8)	
H11A	1.1564	0.2783	0.9249	0.077*	
C12	1.0836 (7)	0.2693 (5)	0.85469 (9)	0.0843 (10)	
H12A	1.2022	0.1999	0.8665	0.101*	
C13	0.9409 (9)	0.1467 (5)	0.82976 (11)	0.1177 (16)	
H13A	1.0259	0.1012	0.8083	0.177*	
H13B	0.8177	0.2114	0.8197	0.177*	
H13C	0.8894	0.0505	0.8457	0.177*	
C14	1.1828 (6)	0.4228 (5)	0.83256 (9)	0.0739 (9)	
C15	1.3934 (7)	0.4772 (6)	0.84071 (9)	0.0895 (11)	
H15A	1.4771	0.4136	0.8586	0.107*	
C16	1.4836 (7)	0.6251 (7)	0.82277 (12)	0.1003 (13)	
H16A	1.6259	0.6592	0.8291	0.120*	
C17	1.3669 (8)	0.7220 (6)	0.79583 (11)	0.0940 (12)	
C18	1.1577 (7)	0.6669 (6)	0.78760 (10)	0.0914 (11)	
H18A	1.0747	0.7299	0.7695	0.110*	
C19	1.0668 (6)	0.5213 (5)	0.80540 (9)	0.0817 (10)	
H19A	0.9242	0.4883	0.7990	0.098*	
C20	1.4631 (9)	0.8829 (7)	0.77589 (13)	0.145 (2)	
H20A	1.6194	0.8836	0.7794	0.218*	
H20B	1.4015	0.9877	0.7873	0.218*	
H20C	1.4294	0.8795	0.7484	0.218*	

### (2) (S)-(+)-2-({[(4-Methylphenyl)ethyl]imino}methyl)naphthalene Atomic displacement parameters (Å^2^)

**Table:**

	*U*^11^	*U*^22^	*U*^33^	*U*^12^	*U*^13^	*U*^23^
N1	0.083 (2)	0.0739 (16)	0.0700 (15)	0.0109 (15)	0.0006 (14)	0.0041 (12)
C1	0.0567 (18)	0.0421 (14)	0.0675 (16)	−0.0004 (14)	−0.0007 (14)	0.0106 (12)
C2	0.0447 (15)	0.0438 (14)	0.0759 (17)	0.0032 (13)	−0.0005 (14)	0.0077 (12)
C3	0.0505 (17)	0.0386 (13)	0.0709 (16)	−0.0027 (13)	0.0021 (14)	0.0073 (12)
C4	0.066 (2)	0.0528 (15)	0.0786 (18)	−0.0022 (16)	−0.0034 (16)	0.0042 (14)
C5	0.087 (3)	0.0661 (19)	0.0754 (19)	−0.007 (2)	−0.0038 (18)	−0.0033 (15)
C6	0.089 (3)	0.0602 (19)	0.089 (2)	−0.0029 (19)	0.020 (2)	−0.0065 (17)
C7	0.061 (2)	0.0476 (15)	0.102 (2)	0.0016 (16)	0.0172 (19)	0.0041 (15)
C8	0.0521 (18)	0.0353 (13)	0.0841 (18)	−0.0017 (13)	0.0042 (15)	0.0067 (13)
C9	0.0454 (17)	0.0469 (15)	0.097 (2)	0.0017 (14)	−0.0027 (16)	0.0096 (14)
C10	0.0571 (19)	0.0500 (16)	0.0776 (18)	0.0000 (15)	−0.0104 (15)	0.0096 (14)
C11	0.066 (2)	0.0494 (15)	0.0765 (18)	0.0066 (15)	−0.0002 (16)	0.0064 (13)
C12	0.095 (3)	0.082 (2)	0.0757 (19)	0.022 (2)	0.0023 (19)	0.0000 (17)
C13	0.164 (5)	0.088 (3)	0.101 (3)	−0.014 (3)	0.006 (3)	−0.019 (2)
C14	0.071 (2)	0.086 (2)	0.0640 (17)	0.0179 (19)	−0.0007 (16)	−0.0085 (17)
C15	0.082 (3)	0.114 (3)	0.072 (2)	0.023 (3)	−0.0097 (19)	−0.014 (2)
C16	0.068 (3)	0.140 (4)	0.093 (3)	−0.002 (3)	0.006 (2)	−0.037 (3)
C17	0.093 (3)	0.104 (3)	0.085 (2)	−0.011 (3)	0.021 (2)	−0.014 (2)
C18	0.093 (3)	0.100 (3)	0.081 (2)	0.006 (2)	0.000 (2)	0.009 (2)
C19	0.073 (2)	0.091 (2)	0.082 (2)	0.006 (2)	−0.0036 (18)	0.0047 (19)
C20	0.151 (5)	0.139 (4)	0.145 (4)	−0.042 (4)	0.042 (4)	−0.005 (3)

### (2) (S)-(+)-2-({[(4-Methylphenyl)ethyl]imino}methyl)naphthalene Geometric parameters (Å, º)

**Table:**

N1—C11	1.270 (3)	C11—H11A	0.9300
N1—C12	1.475 (4)	C12—C14	1.511 (5)
C1—C2	1.367 (4)	C12—C13	1.529 (5)
C1—C10	1.415 (4)	C12—H12A	0.9800
C1—C11	1.463 (4)	C13—H13A	0.9600
C2—C3	1.416 (3)	C13—H13B	0.9600
C2—H2A	0.9300	C13—H13C	0.9600
C3—C4	1.414 (4)	C14—C15	1.376 (5)
C3—C8	1.416 (4)	C14—C19	1.382 (4)
C4—C5	1.360 (4)	C15—C16	1.389 (6)
C4—H4A	0.9300	C15—H15A	0.9300
C5—C6	1.397 (5)	C16—C17	1.373 (6)
C5—H5A	0.9300	C16—H16A	0.9300
C6—C7	1.361 (4)	C17—C18	1.370 (6)
C6—H6A	0.9300	C17—C20	1.513 (6)
C7—C8	1.413 (4)	C18—C19	1.375 (5)
C7—H7A	0.9300	C18—H18A	0.9300
C8—C9	1.419 (4)	C19—H19A	0.9300
C9—C10	1.355 (4)	C20—H20A	0.9600
C9—H9A	0.9300	C20—H20B	0.9600
C10—H10A	0.9300	C20—H20C	0.9600
			
C11—N1—C12	116.8 (3)	N1—C12—C13	108.0 (3)
C2—C1—C10	119.2 (3)	C14—C12—C13	114.7 (3)
C2—C1—C11	119.4 (3)	N1—C12—H12A	108.8
C10—C1—C11	121.5 (3)	C14—C12—H12A	108.8
C1—C2—C3	121.6 (3)	C13—C12—H12A	108.8
C1—C2—H2A	119.2	C12—C13—H13A	109.5
C3—C2—H2A	119.2	C12—C13—H13B	109.5
C4—C3—C8	118.5 (3)	H13A—C13—H13B	109.5
C4—C3—C2	122.8 (3)	C12—C13—H13C	109.5
C8—C3—C2	118.7 (3)	H13A—C13—H13C	109.5
C5—C4—C3	121.0 (3)	H13B—C13—H13C	109.5
C5—C4—H4A	119.5	C15—C14—C19	116.7 (4)
C3—C4—H4A	119.5	C15—C14—C12	120.2 (3)
C4—C5—C6	120.5 (3)	C19—C14—C12	123.0 (3)
C4—C5—H5A	119.7	C14—C15—C16	121.5 (4)
C6—C5—H5A	119.7	C14—C15—H15A	119.3
C7—C6—C5	120.1 (3)	C16—C15—H15A	119.3
C7—C6—H6A	119.9	C17—C16—C15	121.3 (4)
C5—C6—H6A	119.9	C17—C16—H16A	119.4
C6—C7—C8	121.0 (3)	C15—C16—H16A	119.4
C6—C7—H7A	119.5	C18—C17—C16	117.1 (4)
C8—C7—H7A	119.5	C18—C17—C20	120.9 (5)
C7—C8—C3	118.8 (3)	C16—C17—C20	121.9 (5)
C7—C8—C9	122.7 (3)	C17—C18—C19	121.9 (4)
C3—C8—C9	118.5 (3)	C17—C18—H18A	119.0
C10—C9—C8	121.2 (3)	C19—C18—H18A	119.0
C10—C9—H9A	119.4	C18—C19—C14	121.5 (4)
C8—C9—H9A	119.4	C18—C19—H19A	119.3
C9—C10—C1	120.7 (3)	C14—C19—H19A	119.3
C9—C10—H10A	119.6	C17—C20—H20A	109.5
C1—C10—H10A	119.6	C17—C20—H20B	109.5
N1—C11—C1	123.0 (3)	H20A—C20—H20B	109.5
N1—C11—H11A	118.5	C17—C20—H20C	109.5
C1—C11—H11A	118.5	H20A—C20—H20C	109.5
N1—C12—C14	107.7 (3)	H20B—C20—H20C	109.5
			
C10—C1—C2—C3	−2.0 (4)	C12—N1—C11—C1	177.3 (3)
C11—C1—C2—C3	175.9 (2)	C2—C1—C11—N1	−174.3 (3)
C1—C2—C3—C4	−177.1 (3)	C10—C1—C11—N1	3.6 (4)
C1—C2—C3—C8	1.5 (4)	C11—N1—C12—C14	−104.8 (3)
C8—C3—C4—C5	−1.7 (4)	C11—N1—C12—C13	130.8 (3)
C2—C3—C4—C5	176.9 (3)	N1—C12—C14—C15	98.3 (4)
C3—C4—C5—C6	1.4 (5)	C13—C12—C14—C15	−141.5 (4)
C4—C5—C6—C7	−0.3 (5)	N1—C12—C14—C19	−77.7 (4)
C5—C6—C7—C8	−0.5 (5)	C13—C12—C14—C19	42.5 (5)
C6—C7—C8—C3	0.1 (4)	C19—C14—C15—C16	0.7 (5)
C6—C7—C8—C9	−177.8 (3)	C12—C14—C15—C16	−175.6 (3)
C4—C3—C8—C7	1.0 (4)	C14—C15—C16—C17	−0.7 (5)
C2—C3—C8—C7	−177.7 (2)	C15—C16—C17—C18	0.3 (5)
C4—C3—C8—C9	178.9 (2)	C15—C16—C17—C20	−179.7 (4)
C2—C3—C8—C9	0.3 (4)	C16—C17—C18—C19	0.1 (6)
C7—C8—C9—C10	176.4 (3)	C20—C17—C18—C19	−179.9 (4)
C3—C8—C9—C10	−1.5 (4)	C17—C18—C19—C14	0.0 (6)
C8—C9—C10—C1	1.0 (4)	C15—C14—C19—C18	−0.3 (5)
C2—C1—C10—C9	0.7 (4)	C12—C14—C19—C18	175.9 (3)
C11—C1—C10—C9	−177.1 (3)		

### (3) (R)-(–)-2-({[(4-Methoxylphenyl)ethyl]imino}methyl)naphthalene Crystal data

**Table:**

C_20_H_19_NO	*D*_x_ = 1.190 Mg m^−^^3^
*M**_r_* = 289.36	Melting point: 384 K
Orthorhombic, *P*2_1_2_1_2_1_	Cu *K*α radiation, λ = 1.54184 Å
*a* = 6.1094 (5) Å	Cell parameters from 1762 reflections
*b* = 7.7266 (7) Å	θ = 5.2–52.1°
*c* = 34.225 (4) Å	µ = 0.57 mm^−^^1^
*V* = 1615.6 (3) Å^3^	*T* = 298 K
*Z* = 4	Block, colorless
*F*(000) = 616	0.39 × 0.25 × 0.23 mm

### (3) (R)-(–)-2-({[(4-Methoxylphenyl)ethyl]imino}methyl)naphthalene Data collection

**Table:**

Agilent Xcalibur Atlas Gemini diffractometer	3223 independent reflections
Radiation source: Enhance (Cu) X-ray Source	1652 reflections with *I* > 2σ(*I*)
Graphite monochromator	*R*_int_ = 0.068
ω scans	θ_max_ = 74.3°, θ_min_ = 5.2°
Absorption correction: multi-scan (CrysAlis PRO; Agilent, 2013)	*h* = −7→7
*T*_min_ = 0.777, *T*_max_ = 1.000	*k* = −9→9
14921 measured reflections	*l* = −39→41

### (3) (R)-(–)-2-({[(4-Methoxylphenyl)ethyl]imino}methyl)naphthalene Refinement

**Table:**

Refinement on *F*^2^	Primary atom site location: structure-invariant direct methods
Least-squares matrix: full	Secondary atom site location: difference Fourier map
*R*[*F*^2^ > 2σ(*F*^2^)] = 0.063	Hydrogen site location: inferred from neighbouring sites
*wR*(*F*^2^) = 0.155	H-atom parameters constrained
*S* = 1.08	*w* = 1/[σ^2^(*F*_o_^2^) + (0.050*P*)^2^] where *P* = (*F*_o_^2^ + 2*F*_c_^2^)/3
3223 reflections	(Δ/σ)_max_ < 0.001
201 parameters	Δρ_max_ = 0.15 e Å^−^^3^
0 restraints	Δρ_min_ = −0.20 e Å^−^^3^
0 constraints	

### (3) (R)-(–)-2-({[(4-Methoxylphenyl)ethyl]imino}methyl)naphthalene Fractional atomic coordinates and isotropic or equivalent isotropic displacement parameters (Å^2^)

**Table:**

	*x*	*y*	*z*	*U*_iso_*/*U*_eq_	
N1	−0.0201 (6)	0.5080 (5)	0.13755 (13)	0.0761 (12)	
O1	−0.4133 (6)	1.0450 (6)	0.02034 (10)	0.0962 (12)	
C1	0.0868 (7)	0.5184 (6)	0.20532 (14)	0.0630 (12)	
C2	0.0378 (8)	0.4754 (6)	0.24308 (14)	0.0651 (12)	
H2A	−0.0953	0.4216	0.2482	0.078*	
C3	0.1798 (7)	0.5088 (6)	0.27452 (15)	0.0629 (12)	
C4	0.1334 (8)	0.4602 (7)	0.31300 (16)	0.0750 (14)	
H4A	0.0006	0.4068	0.3186	0.090*	
C5	0.2790 (10)	0.4894 (8)	0.34246 (17)	0.0886 (16)	
H5A	0.2457	0.4539	0.3677	0.106*	
C6	0.4796 (10)	0.5729 (8)	0.33493 (18)	0.0903 (17)	
H6A	0.5782	0.5935	0.3551	0.108*	
C7	0.5273 (8)	0.6233 (6)	0.29766 (18)	0.0797 (15)	
H7A	0.6598	0.6785	0.2928	0.096*	
C8	0.3836 (7)	0.5947 (6)	0.26640 (17)	0.0661 (13)	
C9	0.4302 (8)	0.6393 (6)	0.22713 (16)	0.0721 (14)	
H9A	0.5617	0.6944	0.2214	0.086*	
C10	0.2901 (8)	0.6045 (7)	0.19785 (16)	0.0735 (14)	
H10A	0.3257	0.6367	0.1724	0.088*	
C11	−0.0607 (8)	0.4759 (7)	0.17332 (15)	0.0732 (14)	
H11A	−0.1926	0.4221	0.1794	0.088*	
C12	−0.1820 (8)	0.4500 (8)	0.10844 (16)	0.0832 (15)	
H12A	−0.3096	0.4024	0.1221	0.100*	
C13	−0.0727 (11)	0.3053 (7)	0.08438 (16)	0.108 (2)	
H13A	−0.1724	0.2655	0.0646	0.163*	
H13B	−0.0352	0.2107	0.1013	0.163*	
H13C	0.0574	0.3493	0.0722	0.163*	
C14	−0.2531 (9)	0.6019 (8)	0.08414 (15)	0.0776 (15)	
C15	−0.1076 (9)	0.6836 (8)	0.05893 (16)	0.0901 (17)	
H15A	0.0326	0.6386	0.0560	0.108*	
C16	−0.1656 (8)	0.8295 (8)	0.03809 (18)	0.0862 (16)	
H16A	−0.0644	0.8820	0.0216	0.103*	
C17	−0.3741 (9)	0.8978 (8)	0.04169 (16)	0.0814 (15)	
C18	−0.5227 (9)	0.8167 (8)	0.06551 (15)	0.0879 (17)	
H18A	−0.6642	0.8599	0.0678	0.105*	
C19	−0.4615 (9)	0.6698 (8)	0.08625 (15)	0.0836 (16)	
H19A	−0.5645	0.6155	0.1021	0.100*	
C20	−0.6143 (10)	1.1351 (9)	0.0268 (2)	0.132 (3)	
H20A	−0.6120	1.2429	0.0129	0.198*	
H20B	−0.6316	1.1571	0.0543	0.198*	
H20C	−0.7342	1.0657	0.0177	0.198*	

### (3) (R)-(–)-2-({[(4-Methoxylphenyl)ethyl]imino}methyl)naphthalene Atomic displacement parameters (Å^2^)

**Table:**

	*U*^11^	*U*^22^	*U*^33^	*U*^12^	*U*^13^	*U*^23^
N1	0.074 (3)	0.074 (3)	0.080 (3)	−0.001 (2)	0.002 (2)	−0.001 (2)
O1	0.085 (3)	0.095 (3)	0.108 (3)	0.001 (2)	−0.004 (2)	0.012 (2)
C1	0.057 (3)	0.053 (3)	0.080 (3)	0.006 (3)	0.005 (3)	−0.003 (3)
C2	0.054 (3)	0.057 (3)	0.085 (3)	0.005 (2)	0.008 (3)	0.000 (3)
C3	0.060 (3)	0.051 (3)	0.078 (3)	0.007 (2)	0.001 (3)	−0.004 (3)
C4	0.071 (3)	0.066 (3)	0.088 (4)	0.004 (3)	0.000 (3)	0.002 (3)
C5	0.099 (4)	0.076 (4)	0.091 (4)	0.017 (4)	−0.001 (3)	−0.001 (3)
C6	0.086 (4)	0.087 (4)	0.097 (5)	0.009 (4)	−0.018 (4)	−0.008 (3)
C7	0.065 (3)	0.065 (3)	0.109 (4)	0.007 (3)	−0.008 (4)	−0.007 (3)
C8	0.053 (3)	0.050 (3)	0.096 (4)	0.006 (2)	−0.002 (3)	−0.003 (3)
C9	0.058 (3)	0.056 (3)	0.102 (4)	0.001 (3)	0.009 (3)	0.002 (3)
C10	0.070 (3)	0.064 (3)	0.086 (4)	0.003 (3)	0.008 (3)	0.006 (3)
C11	0.061 (3)	0.066 (3)	0.092 (4)	0.005 (3)	0.002 (3)	−0.006 (3)
C12	0.080 (3)	0.086 (4)	0.084 (4)	−0.015 (3)	0.000 (3)	−0.004 (3)
C13	0.143 (6)	0.074 (4)	0.108 (4)	0.002 (5)	−0.003 (4)	−0.010 (3)
C14	0.073 (3)	0.084 (4)	0.076 (4)	−0.014 (3)	−0.001 (3)	−0.005 (3)
C15	0.067 (3)	0.084 (4)	0.119 (5)	0.000 (3)	0.010 (3)	0.007 (4)
C16	0.065 (3)	0.076 (4)	0.117 (5)	−0.010 (3)	0.019 (3)	0.003 (3)
C17	0.074 (3)	0.093 (4)	0.078 (4)	−0.011 (3)	−0.008 (3)	−0.004 (3)
C18	0.061 (3)	0.114 (5)	0.088 (4)	−0.005 (3)	−0.002 (3)	0.003 (4)
C19	0.068 (3)	0.106 (5)	0.076 (4)	−0.014 (3)	0.004 (3)	0.005 (3)
C20	0.093 (5)	0.142 (7)	0.161 (7)	0.034 (5)	−0.002 (4)	0.029 (5)

### (3) (R)-(–)-2-({[(4-Methoxylphenyl)ethyl]imino}methyl)naphthalene Geometric parameters (Å, º)

**Table:**

N1—C11	1.273 (5)	C10—H10A	0.9300
N1—C12	1.474 (6)	C11—H11A	0.9300
O1—C17	1.373 (6)	C12—C14	1.503 (7)
O1—C20	1.429 (6)	C12—C13	1.541 (7)
C1—C2	1.367 (6)	C12—H12A	0.9800
C1—C10	1.432 (6)	C13—H13A	0.9600
C1—C11	1.456 (6)	C13—H13B	0.9600
C2—C3	1.406 (6)	C13—H13C	0.9600
C2—H2A	0.9300	C14—C19	1.379 (7)
C3—C4	1.399 (6)	C14—C15	1.390 (7)
C3—C8	1.438 (6)	C15—C16	1.380 (7)
C4—C5	1.364 (7)	C15—H15A	0.9300
C4—H4A	0.9300	C16—C17	1.385 (7)
C5—C6	1.408 (8)	C16—H16A	0.9300
C5—H5A	0.9300	C17—C18	1.372 (7)
C6—C7	1.365 (6)	C18—C19	1.390 (7)
C6—H6A	0.9300	C18—H18A	0.9300
C7—C8	1.401 (6)	C19—H19A	0.9300
C7—H7A	0.9300	C20—H20A	0.9600
C8—C9	1.416 (6)	C20—H20B	0.9600
C9—C10	1.345 (6)	C20—H20C	0.9600
C9—H9A	0.9300		
			
C11—N1—C12	117.4 (4)	N1—C12—C13	106.9 (5)
C17—O1—C20	118.1 (5)	C14—C12—C13	113.4 (4)
C2—C1—C10	118.1 (5)	N1—C12—H12A	109.0
C2—C1—C11	121.4 (4)	C14—C12—H12A	109.0
C10—C1—C11	120.5 (5)	C13—C12—H12A	109.0
C1—C2—C3	122.9 (4)	C12—C13—H13A	109.5
C1—C2—H2A	118.5	C12—C13—H13B	109.5
C3—C2—H2A	118.5	H13A—C13—H13B	109.5
C4—C3—C2	123.1 (4)	C12—C13—H13C	109.5
C4—C3—C8	118.8 (5)	H13A—C13—H13C	109.5
C2—C3—C8	118.1 (4)	H13B—C13—H13C	109.5
C5—C4—C3	121.3 (5)	C19—C14—C15	116.8 (5)
C5—C4—H4A	119.4	C19—C14—C12	122.3 (5)
C3—C4—H4A	119.4	C15—C14—C12	120.9 (5)
C4—C5—C6	120.6 (6)	C16—C15—C14	121.8 (5)
C4—C5—H5A	119.7	C16—C15—H15A	119.1
C6—C5—H5A	119.7	C14—C15—H15A	119.1
C7—C6—C5	119.2 (5)	C15—C16—C17	120.1 (5)
C7—C6—H6A	120.4	C15—C16—H16A	120.0
C5—C6—H6A	120.4	C17—C16—H16A	120.0
C6—C7—C8	122.3 (5)	C18—C17—O1	125.4 (5)
C6—C7—H7A	118.8	C18—C17—C16	119.2 (6)
C8—C7—H7A	118.8	O1—C17—C16	115.4 (5)
C7—C8—C9	124.1 (5)	C17—C18—C19	119.9 (5)
C7—C8—C3	117.9 (5)	C17—C18—H18A	120.1
C9—C8—C3	118.0 (5)	C19—C18—H18A	120.1
C10—C9—C8	122.0 (5)	C14—C19—C18	122.2 (5)
C10—C9—H9A	119.0	C14—C19—H19A	118.9
C8—C9—H9A	119.0	C18—C19—H19A	118.9
C9—C10—C1	120.8 (5)	O1—C20—H20A	109.5
C9—C10—H10A	119.6	O1—C20—H20B	109.5
C1—C10—H10A	119.6	H20A—C20—H20B	109.5
N1—C11—C1	124.0 (5)	O1—C20—H20C	109.5
N1—C11—H11A	118.0	H20A—C20—H20C	109.5
C1—C11—H11A	118.0	H20B—C20—H20C	109.5
N1—C12—C14	109.3 (5)		
			
C10—C1—C2—C3	−1.3 (6)	C2—C1—C11—N1	−177.7 (4)
C11—C1—C2—C3	177.7 (4)	C10—C1—C11—N1	1.3 (7)
C1—C2—C3—C4	−177.9 (4)	C11—N1—C12—C14	124.6 (5)
C1—C2—C3—C8	1.1 (6)	C11—N1—C12—C13	−112.3 (5)
C2—C3—C4—C5	177.6 (5)	N1—C12—C14—C19	−111.5 (5)
C8—C3—C4—C5	−1.4 (7)	C13—C12—C14—C19	129.3 (6)
C3—C4—C5—C6	1.2 (8)	N1—C12—C14—C15	67.0 (6)
C4—C5—C6—C7	−0.5 (8)	C13—C12—C14—C15	−52.2 (7)
C5—C6—C7—C8	−0.1 (8)	C19—C14—C15—C16	2.4 (8)
C6—C7—C8—C9	−177.7 (5)	C12—C14—C15—C16	−176.1 (5)
C6—C7—C8—C3	−0.1 (7)	C14—C15—C16—C17	−0.6 (9)
C4—C3—C8—C7	0.8 (6)	C20—O1—C17—C18	8.5 (8)
C2—C3—C8—C7	−178.2 (4)	C20—O1—C17—C16	−171.6 (5)
C4—C3—C8—C9	178.5 (4)	C15—C16—C17—C18	−1.3 (8)
C2—C3—C8—C9	−0.5 (6)	C15—C16—C17—O1	178.7 (5)
C7—C8—C9—C10	177.8 (5)	O1—C17—C18—C19	−178.8 (5)
C3—C8—C9—C10	0.3 (6)	C16—C17—C18—C19	1.3 (8)
C8—C9—C10—C1	−0.6 (7)	C15—C14—C19—C18	−2.5 (8)
C2—C1—C10—C9	1.1 (7)	C12—C14—C19—C18	176.0 (5)
C11—C1—C10—C9	−177.9 (4)	C17—C18—C19—C14	0.7 (8)
C12—N1—C11—C1	177.4 (4)		

### (4) (R)-(–)-2-({[(4-Fluorophenyl)ethyl]imino}methyl)naphthalene Crystal data

**Table:**

C_19_H_16_FN	*D*_x_ = 1.226 Mg m^−^^3^
*M**_r_* = 277.33	Melting point: 395 K
Monoclinic, *P*2_1_	Mo *K*α radiation, λ = 0.71073 Å
*a* = 7.5950 (11) Å	Cell parameters from 1285 reflections
*b* = 5.8997 (9) Å	θ = 3.3–22.0°
*c* = 16.996 (3) Å	µ = 0.08 mm^−^^1^
β = 99.420 (15)°	*T* = 298 K
*V* = 751.3 (2) Å^3^	Prism, colorless
*Z* = 2	0.74 × 0.21 × 0.09 mm
*F*(000) = 292	

### (4) (R)-(–)-2-({[(4-Fluorophenyl)ethyl]imino}methyl)naphthalene Data collection

**Table:**

Agilent Xcalibur Atlas Gemini diffractometer	2627 independent reflections
Radiation source: Enhance (Mo) X-ray Source	1640 reflections with *I* > 2σ(*I*)
Graphite monochromator	*R*_int_ = 0.051
Detector resolution: 10.5564 pixels mm^-1^	θ_max_ = 25.0°, θ_min_ = 3.2°
ω scans	*h* = −9→9
Absorption correction: analytical (CrysAlis PRO; Agilent, 2013)	*k* = −7→6
*T*_min_ = 0.970, *T*_max_ = 0.994	*l* = −20→20
7823 measured reflections	

### (4) (R)-(–)-2-({[(4-Fluorophenyl)ethyl]imino}methyl)naphthalene Refinement

**Table:**

Refinement on *F*^2^	Primary atom site location: structure-invariant direct methods
Least-squares matrix: full	Secondary atom site location: difference Fourier map
*R*[*F*^2^ > 2σ(*F*^2^)] = 0.066	Hydrogen site location: inferred from neighbouring sites
*wR*(*F*^2^) = 0.153	H-atom parameters constrained
*S* = 1.25	*w* = 1/[σ^2^(*F*_o_^2^) + (0.050*P*)^2^] where *P* = (*F*_o_^2^ + 2*F*_c_^2^)/3
2627 reflections	(Δ/σ)_max_ < 0.001
190 parameters	Δρ_max_ = 0.23 e Å^−^^3^
1 restraint	Δρ_min_ = −0.19 e Å^−^^3^
0 constraints	

### (4) (R)-(–)-2-({[(4-Fluorophenyl)ethyl]imino}methyl)naphthalene Fractional atomic coordinates and isotropic or equivalent isotropic displacement parameters (Å^2^)

**Table:**

	*x*	*y*	*z*	*U*_iso_*/*U*_eq_	
F1	0.0966 (5)	0.5690 (9)	0.4846 (2)	0.1060 (14)	
N1	0.7143 (6)	0.6311 (8)	0.7811 (3)	0.0578 (12)	
C1	0.7641 (6)	0.6386 (9)	0.9244 (3)	0.0446 (13)	
C2	0.8265 (6)	0.5262 (9)	0.9935 (3)	0.0468 (12)	
H2A	0.8832	0.3874	0.9907	0.056*	
C3	0.8080 (6)	0.6138 (8)	1.0693 (3)	0.0442 (12)	
C4	0.8659 (7)	0.4961 (10)	1.1407 (3)	0.0592 (15)	
H4A	0.9260	0.3592	1.1395	0.071*	
C5	0.8346 (8)	0.5810 (12)	1.2119 (4)	0.0747 (18)	
H5A	0.8723	0.5002	1.2586	0.090*	
C6	0.7465 (8)	0.7884 (11)	1.2153 (4)	0.072 (2)	
H6A	0.7247	0.8438	1.2640	0.086*	
C7	0.6934 (7)	0.9077 (10)	1.1480 (4)	0.0638 (17)	
H7A	0.6375	1.0469	1.1513	0.077*	
C8	0.7203 (6)	0.8270 (9)	1.0726 (3)	0.0460 (13)	
C9	0.6637 (7)	0.9449 (9)	1.0009 (4)	0.0576 (16)	
H9A	0.6122	1.0874	1.0027	0.069*	
C10	0.6824 (7)	0.8554 (9)	0.9292 (3)	0.0552 (15)	
H10A	0.6416	0.9358	0.8828	0.066*	
C11	0.7749 (6)	0.5335 (10)	0.8471 (3)	0.0522 (14)	
H11A	0.8275	0.3914	0.8461	0.063*	
C12	0.7282 (7)	0.5075 (11)	0.7078 (3)	0.0632 (16)	
H12A	0.7562	0.3483	0.7207	0.076*	
C13	0.8789 (7)	0.6126 (16)	0.6692 (4)	0.094 (2)	
H13A	0.8889	0.5323	0.6210	0.142*	
H13B	0.8522	0.7689	0.6570	0.142*	
H13C	0.9896	0.6019	0.7055	0.142*	
C14	0.5538 (7)	0.5214 (10)	0.6512 (3)	0.0546 (14)	
C15	0.5010 (9)	0.3482 (11)	0.5979 (3)	0.0685 (17)	
H15A	0.5708	0.2182	0.5996	0.082*	
C16	0.3472 (9)	0.3628 (13)	0.5419 (4)	0.0777 (19)	
H16A	0.3135	0.2450	0.5063	0.093*	
C17	0.2474 (8)	0.5521 (13)	0.5404 (4)	0.0712 (17)	
C18	0.2909 (8)	0.7271 (12)	0.5918 (4)	0.0662 (17)	
H18A	0.2181	0.8544	0.5897	0.079*	
C19	0.4457 (7)	0.7122 (10)	0.6472 (3)	0.0616 (16)	
H19A	0.4777	0.8318	0.6823	0.074*	

### (4) (R)-(–)-2-({[(4-Fluorophenyl)ethyl]imino}methyl)naphthalene Atomic displacement parameters (Å^2^)

**Table:**

	*U*^11^	*U*^22^	*U*^33^	*U*^12^	*U*^13^	*U*^23^
F1	0.078 (2)	0.141 (4)	0.089 (3)	−0.014 (3)	−0.014 (2)	−0.005 (2)
N1	0.058 (3)	0.062 (3)	0.053 (3)	0.000 (2)	0.005 (2)	0.001 (3)
C1	0.037 (3)	0.043 (3)	0.052 (3)	−0.002 (3)	0.004 (2)	0.000 (3)
C2	0.043 (3)	0.038 (3)	0.061 (4)	0.004 (2)	0.011 (2)	0.002 (3)
C3	0.039 (3)	0.041 (3)	0.054 (3)	−0.001 (3)	0.011 (2)	−0.001 (3)
C4	0.064 (3)	0.056 (4)	0.060 (4)	0.008 (3)	0.020 (3)	0.009 (3)
C5	0.078 (4)	0.092 (5)	0.057 (4)	0.011 (4)	0.017 (3)	0.006 (4)
C6	0.074 (4)	0.081 (5)	0.064 (4)	−0.005 (4)	0.022 (4)	−0.017 (4)
C7	0.058 (4)	0.056 (4)	0.080 (5)	0.000 (3)	0.018 (3)	−0.011 (3)
C8	0.039 (3)	0.042 (3)	0.057 (4)	−0.004 (3)	0.007 (2)	−0.003 (3)
C9	0.053 (3)	0.035 (3)	0.086 (5)	0.004 (3)	0.016 (3)	−0.003 (3)
C10	0.054 (3)	0.047 (4)	0.061 (4)	0.003 (3)	−0.001 (3)	0.006 (3)
C11	0.048 (3)	0.053 (3)	0.056 (4)	0.005 (3)	0.009 (2)	−0.002 (3)
C12	0.065 (4)	0.068 (4)	0.055 (4)	0.008 (3)	0.008 (3)	0.004 (3)
C13	0.060 (3)	0.154 (7)	0.072 (4)	−0.001 (5)	0.019 (3)	0.013 (5)
C14	0.060 (3)	0.059 (4)	0.049 (3)	−0.001 (3)	0.020 (3)	0.003 (3)
C15	0.086 (4)	0.062 (4)	0.062 (4)	0.002 (4)	0.026 (4)	−0.009 (3)
C16	0.089 (5)	0.084 (5)	0.059 (4)	−0.017 (4)	0.011 (4)	−0.016 (4)
C17	0.062 (4)	0.090 (5)	0.060 (4)	−0.011 (4)	0.005 (3)	0.000 (4)
C18	0.056 (4)	0.074 (4)	0.069 (4)	0.002 (3)	0.013 (3)	0.009 (4)
C19	0.065 (4)	0.063 (4)	0.057 (4)	−0.006 (3)	0.012 (3)	−0.005 (3)

### (4) (R)-(–)-2-({[(4-Fluorophenyl)ethyl]imino}methyl)naphthalene Geometric parameters (Å, º)

**Table:**

F1—C17	1.367 (6)	C9—H9A	0.9300
N1—C11	1.277 (6)	C10—H10A	0.9300
N1—C12	1.462 (7)	C11—H11A	0.9300
C1—C2	1.364 (7)	C12—C14	1.507 (7)
C1—C10	1.430 (7)	C12—C13	1.540 (8)
C1—C11	1.467 (7)	C12—H12A	0.9800
C2—C3	1.417 (7)	C13—H13A	0.9600
C2—H2A	0.9300	C13—H13B	0.9600
C3—C4	1.405 (7)	C13—H13C	0.9600
C3—C8	1.428 (7)	C14—C15	1.381 (7)
C4—C5	1.365 (7)	C14—C19	1.389 (7)
C4—H4A	0.9300	C15—C16	1.383 (8)
C5—C6	1.400 (9)	C15—H15A	0.9300
C5—H5A	0.9300	C16—C17	1.348 (9)
C6—C7	1.347 (8)	C16—H16A	0.9300
C6—H6A	0.9300	C17—C18	1.357 (9)
C7—C8	1.413 (7)	C18—C19	1.384 (8)
C7—H7A	0.9300	C18—H18A	0.9300
C8—C9	1.408 (7)	C19—H19A	0.9300
C9—C10	1.356 (7)		
			
C11—N1—C12	117.2 (5)	N1—C11—H11A	119.0
C2—C1—C10	118.6 (5)	C1—C11—H11A	119.0
C2—C1—C11	120.2 (5)	N1—C12—C14	109.8 (5)
C10—C1—C11	121.2 (5)	N1—C12—C13	109.0 (5)
C1—C2—C3	122.3 (5)	C14—C12—C13	110.1 (5)
C1—C2—H2A	118.9	N1—C12—H12A	109.3
C3—C2—H2A	118.9	C14—C12—H12A	109.3
C4—C3—C2	122.8 (5)	C13—C12—H12A	109.3
C4—C3—C8	118.9 (5)	C12—C13—H13A	109.5
C2—C3—C8	118.2 (5)	C12—C13—H13B	109.5
C5—C4—C3	120.6 (5)	H13A—C13—H13B	109.5
C5—C4—H4A	119.7	C12—C13—H13C	109.5
C3—C4—H4A	119.7	H13A—C13—H13C	109.5
C4—C5—C6	120.7 (6)	H13B—C13—H13C	109.5
C4—C5—H5A	119.7	C15—C14—C19	117.6 (5)
C6—C5—H5A	119.7	C15—C14—C12	120.6 (6)
C7—C6—C5	120.0 (6)	C19—C14—C12	121.7 (5)
C7—C6—H6A	120.0	C14—C15—C16	121.8 (6)
C5—C6—H6A	120.0	C14—C15—H15A	119.1
C6—C7—C8	121.7 (6)	C16—C15—H15A	119.1
C6—C7—H7A	119.1	C17—C16—C15	118.3 (6)
C8—C7—H7A	119.1	C17—C16—H16A	120.8
C9—C8—C7	123.3 (5)	C15—C16—H16A	120.8
C9—C8—C3	118.7 (5)	C16—C17—C18	122.8 (6)
C7—C8—C3	118.0 (5)	C16—C17—F1	118.6 (7)
C10—C9—C8	121.5 (5)	C18—C17—F1	118.6 (7)
C10—C9—H9A	119.2	C17—C18—C19	118.7 (6)
C8—C9—H9A	119.2	C17—C18—H18A	120.7
C9—C10—C1	120.7 (5)	C19—C18—H18A	120.7
C9—C10—H10A	119.7	C18—C19—C14	120.9 (6)
C1—C10—H10A	119.7	C18—C19—H19A	119.5
N1—C11—C1	122.0 (5)	C14—C19—H19A	119.5
			
C10—C1—C2—C3	2.0 (7)	C12—N1—C11—C1	−178.4 (4)
C11—C1—C2—C3	−175.6 (4)	C2—C1—C11—N1	178.4 (4)
C1—C2—C3—C4	177.6 (4)	C10—C1—C11—N1	0.9 (7)
C1—C2—C3—C8	0.1 (7)	C11—N1—C12—C14	134.2 (5)
C2—C3—C4—C5	−175.7 (5)	C11—N1—C12—C13	−105.2 (6)
C8—C3—C4—C5	1.7 (7)	N1—C12—C14—C15	−150.1 (5)
C3—C4—C5—C6	−0.9 (9)	C13—C12—C14—C15	90.0 (7)
C4—C5—C6—C7	−0.8 (9)	N1—C12—C14—C19	33.7 (7)
C5—C6—C7—C8	1.5 (9)	C13—C12—C14—C19	−86.2 (7)
C6—C7—C8—C9	178.8 (5)	C19—C14—C15—C16	0.4 (8)
C6—C7—C8—C3	−0.6 (8)	C12—C14—C15—C16	−175.9 (5)
C4—C3—C8—C9	179.6 (5)	C14—C15—C16—C17	−0.2 (9)
C2—C3—C8—C9	−2.8 (6)	C15—C16—C17—C18	−0.6 (9)
C4—C3—C8—C7	−1.0 (7)	C15—C16—C17—F1	179.1 (5)
C2—C3—C8—C7	176.6 (5)	C16—C17—C18—C19	1.0 (9)
C7—C8—C9—C10	−176.0 (5)	F1—C17—C18—C19	−178.6 (5)
C3—C8—C9—C10	3.4 (7)	C17—C18—C19—C14	−0.8 (8)
C8—C9—C10—C1	−1.3 (8)	C15—C14—C19—C18	0.1 (8)
C2—C1—C10—C9	−1.5 (7)	C12—C14—C19—C18	176.3 (5)
C11—C1—C10—C9	176.1 (5)		

### (5) (S)-(+)-2-({[(4-Chlorophenyl)ethyl]imino}methyl)naphthalene Crystal data

**Table:**

C_19_H_16_ClN	*D*_x_ = 1.250 Mg m^−^^3^
*M**_r_* = 293.78	Melting point: 400 K
Orthorhombic, *P*2_1_2_1_2_1_	Mo *K*α radiation, λ = 0.71073 Å
*a* = 6.0567 (5) Å	Cell parameters from 3689 reflections
*b* = 7.6139 (5) Å	θ = 3.6–22.5°
*c* = 33.853 (3) Å	µ = 0.24 mm^−^^1^
*V* = 1561.1 (2) Å^3^	*T* = 298 K
*Z* = 4	Prism, colorless
*F*(000) = 616	0.38 × 0.32 × 0.14 mm

### (5) (S)-(+)-2-({[(4-Chlorophenyl)ethyl]imino}methyl)naphthalene Data collection

**Table:**

Agilent Xcalibur Atlas Gemini diffractometer	2752 independent reflections
Radiation source: Enhance (Mo) X-ray Source	2014 reflections with *I* > 2σ(*I*)
Graphite monochromator	*R*_int_ = 0.065
Detector resolution: 10.5564 pixels mm^-1^	θ_max_ = 25.0°, θ_min_ = 2.9°
ω scans	*h* = −7→7
Absorption correction: analytical (CrysAlis PRO; Agilent, 2013)	*k* = −9→9
*T*_min_ = 0.437, *T*_max_ = 0.703	*l* = −40→40
21167 measured reflections	

### (5) (S)-(+)-2-({[(4-Chlorophenyl)ethyl]imino}methyl)naphthalene Refinement

**Table:**

Refinement on *F*^2^	Secondary atom site location: difference Fourier map
Least-squares matrix: full	Hydrogen site location: inferred from neighbouring sites
*R*[*F*^2^ > 2σ(*F*^2^)] = 0.057	H-atom parameters constrained
*wR*(*F*^2^) = 0.139	*w* = 1/[σ^2^(*F*_o_^2^) + (0.0534*P*)^2^ + 0.452*P*] where *P* = (*F*_o_^2^ + 2*F*_c_^2^)/3
*S* = 1.07	(Δ/σ)_max_ < 0.001
2752 reflections	Δρ_max_ = 0.18 e Å^−^^3^
191 parameters	Δρ_min_ = −0.29 e Å^−^^3^
0 restraints	Absolute structure: Flack *x* determined using 645 quotients [(*I*^+^)-(*I*^-^)]/[(*I*^+^)+(*I*^-^)] (Parsons *et al.*, 2013)
0 constraints	Absolute structure parameter: 0.02 (6)
Primary atom site location: structure-invariant direct methods	

### (5) (S)-(+)-2-({[(4-Chlorophenyl)ethyl]imino}methyl)naphthalene Fractional atomic coordinates and isotropic or equivalent isotropic displacement parameters (Å^2^)

**Table:**

	*x*	*y*	*z*	*U*_iso_*/*U*_eq_	
Cl1	0.9465 (4)	0.9082 (3)	0.27391 (6)	0.1435 (8)	
N1	0.4109 (7)	0.3451 (5)	0.38543 (11)	0.0696 (10)	
C1	0.3744 (6)	0.4088 (5)	0.45465 (12)	0.0465 (9)	
C2	0.4749 (6)	0.4065 (5)	0.49087 (11)	0.0460 (9)	
H2A	0.6113	0.3515	0.4934	0.055*	
C3	0.3788 (6)	0.4845 (4)	0.52462 (11)	0.0443 (9)	
C4	0.4842 (7)	0.4899 (5)	0.56185 (11)	0.0558 (10)	
H4A	0.6207	0.4358	0.5649	0.067*	
C5	0.3899 (8)	0.5727 (6)	0.59339 (13)	0.0672 (12)	
H5A	0.4637	0.5767	0.6175	0.081*	
C6	0.1815 (9)	0.6517 (6)	0.58949 (15)	0.0709 (13)	
H6A	0.1174	0.7074	0.6111	0.085*	
C7	0.0722 (7)	0.6473 (5)	0.55418 (14)	0.0621 (12)	
H7A	−0.0659	0.6998	0.5521	0.075*	
C8	0.1658 (6)	0.5642 (5)	0.52077 (12)	0.0488 (10)	
C9	0.0627 (7)	0.5609 (5)	0.48332 (13)	0.0558 (11)	
H9A	−0.0765	0.6108	0.4805	0.067*	
C10	0.1610 (7)	0.4874 (5)	0.45155 (12)	0.0537 (10)	
H10A	0.0887	0.4879	0.4273	0.064*	
C11	0.4872 (7)	0.3361 (5)	0.42020 (12)	0.0552 (10)	
H11A	0.6222	0.2804	0.4240	0.066*	
C12	0.5487 (10)	0.2724 (7)	0.35352 (13)	0.0784 (14)	
H12A	0.6675	0.2031	0.3655	0.094*	
C13	0.4087 (13)	0.1510 (7)	0.32800 (17)	0.111 (2)	
H13A	0.3580	0.0538	0.3436	0.167*	
H13B	0.4959	0.1080	0.3064	0.167*	
H13C	0.2841	0.2146	0.3179	0.167*	
C14	0.6516 (8)	0.4269 (7)	0.33160 (12)	0.0681 (12)	
C15	0.5323 (8)	0.5253 (7)	0.30452 (13)	0.0717 (13)	
H15A	0.3893	0.4914	0.2980	0.086*	
C16	0.6227 (9)	0.6738 (8)	0.28686 (15)	0.0820 (15)	
H16A	0.5407	0.7389	0.2688	0.098*	
C17	0.8316 (10)	0.7226 (8)	0.29619 (16)	0.0865 (16)	
C18	0.9557 (9)	0.6285 (9)	0.32290 (16)	0.0914 (18)	
H18A	1.0983	0.6637	0.3293	0.110*	
C19	0.8638 (9)	0.4801 (8)	0.34011 (14)	0.0829 (15)	
H19A	0.9476	0.4149	0.3579	0.100*	

### (5) (S)-(+)-2-({[(4-Chlorophenyl)ethyl]imino}methyl)naphthalene Atomic displacement parameters (Å^2^)

**Table:**

	*U*^11^	*U*^22^	*U*^33^	*U*^12^	*U*^13^	*U*^23^
Cl1	0.1497 (17)	0.1490 (17)	0.1320 (15)	−0.0588 (14)	0.0173 (13)	0.0114 (12)
N1	0.082 (3)	0.068 (2)	0.059 (2)	0.013 (2)	−0.003 (2)	0.0038 (18)
C1	0.044 (2)	0.0332 (18)	0.062 (2)	−0.0013 (16)	−0.0031 (19)	0.0092 (17)
C2	0.0378 (19)	0.0349 (18)	0.065 (2)	0.0024 (17)	−0.0001 (19)	0.0088 (16)
C3	0.042 (2)	0.0295 (17)	0.062 (2)	−0.0060 (16)	0.0005 (18)	0.0073 (17)
C4	0.054 (2)	0.047 (2)	0.066 (3)	−0.003 (2)	−0.002 (2)	0.003 (2)
C5	0.075 (3)	0.055 (2)	0.071 (3)	−0.008 (3)	−0.004 (2)	−0.003 (2)
C6	0.085 (4)	0.050 (2)	0.078 (3)	−0.003 (3)	0.018 (3)	−0.007 (2)
C7	0.053 (2)	0.042 (2)	0.091 (3)	0.002 (2)	0.018 (2)	0.005 (2)
C8	0.043 (2)	0.0279 (18)	0.076 (3)	−0.0029 (17)	0.0058 (19)	0.0073 (18)
C9	0.039 (2)	0.040 (2)	0.088 (3)	−0.0001 (19)	−0.005 (2)	0.010 (2)
C10	0.052 (2)	0.043 (2)	0.065 (3)	−0.004 (2)	−0.011 (2)	0.0100 (19)
C11	0.058 (2)	0.044 (2)	0.064 (3)	0.004 (2)	0.002 (2)	0.0036 (18)
C12	0.094 (4)	0.078 (3)	0.062 (3)	0.026 (3)	0.000 (3)	0.000 (2)
C13	0.164 (6)	0.077 (3)	0.093 (4)	−0.011 (4)	0.006 (4)	−0.020 (3)
C14	0.070 (3)	0.079 (3)	0.056 (3)	0.024 (3)	−0.001 (2)	−0.011 (2)
C15	0.065 (3)	0.085 (3)	0.065 (3)	0.003 (3)	−0.005 (2)	0.001 (3)
C16	0.082 (4)	0.093 (4)	0.070 (3)	0.004 (3)	−0.008 (3)	0.006 (3)
C17	0.083 (4)	0.110 (4)	0.066 (3)	−0.017 (4)	0.012 (3)	−0.013 (3)
C18	0.061 (3)	0.130 (5)	0.083 (4)	−0.001 (4)	0.000 (3)	−0.034 (4)
C19	0.075 (4)	0.110 (4)	0.064 (3)	0.019 (3)	−0.006 (3)	−0.014 (3)

### (5) (S)-(+)-2-({[(4-Chlorophenyl)ethyl]imino}methyl)naphthalene Geometric parameters (Å, º)

**Table:**

Cl1—C17	1.746 (6)	C9—H9A	0.9300
N1—C11	1.266 (5)	C10—H10A	0.9300
N1—C12	1.473 (6)	C11—H11A	0.9300
C1—C2	1.369 (5)	C12—C13	1.523 (7)
C1—C10	1.428 (5)	C12—C14	1.524 (7)
C1—C11	1.461 (5)	C12—H12A	0.9800
C2—C3	1.413 (5)	C13—H13A	0.9600
C2—H2A	0.9300	C13—H13B	0.9600
C3—C4	1.413 (5)	C13—H13C	0.9600
C3—C8	1.432 (5)	C14—C19	1.378 (7)
C4—C5	1.365 (6)	C14—C15	1.387 (6)
C4—H4A	0.9300	C15—C16	1.391 (7)
C5—C6	1.405 (7)	C15—H15A	0.9300
C5—H5A	0.9300	C16—C17	1.356 (8)
C6—C7	1.367 (6)	C16—H16A	0.9300
C6—H6A	0.9300	C17—C18	1.377 (8)
C7—C8	1.415 (5)	C18—C19	1.388 (8)
C7—H7A	0.9300	C18—H18A	0.9300
C8—C9	1.413 (5)	C19—H19A	0.9300
C9—C10	1.351 (5)		
			
C11—N1—C12	117.0 (4)	N1—C11—H11A	118.3
C2—C1—C10	118.3 (4)	C1—C11—H11A	118.3
C2—C1—C11	120.1 (3)	N1—C12—C13	109.2 (5)
C10—C1—C11	121.6 (4)	N1—C12—C14	107.4 (4)
C1—C2—C3	122.4 (3)	C13—C12—C14	114.8 (4)
C1—C2—H2A	118.8	N1—C12—H12A	108.4
C3—C2—H2A	118.8	C13—C12—H12A	108.4
C2—C3—C4	123.2 (3)	C14—C12—H12A	108.4
C2—C3—C8	118.4 (3)	C12—C13—H13A	109.5
C4—C3—C8	118.4 (3)	C12—C13—H13B	109.5
C5—C4—C3	121.5 (4)	H13A—C13—H13B	109.5
C5—C4—H4A	119.3	C12—C13—H13C	109.5
C3—C4—H4A	119.3	H13A—C13—H13C	109.5
C4—C5—C6	120.0 (4)	H13B—C13—H13C	109.5
C4—C5—H5A	120.0	C19—C14—C15	117.7 (5)
C6—C5—H5A	120.0	C19—C14—C12	120.5 (5)
C7—C6—C5	120.5 (4)	C15—C14—C12	121.7 (5)
C7—C6—H6A	119.8	C14—C15—C16	121.2 (5)
C5—C6—H6A	119.8	C14—C15—H15A	119.4
C6—C7—C8	121.1 (4)	C16—C15—H15A	119.4
C6—C7—H7A	119.5	C17—C16—C15	119.3 (5)
C8—C7—H7A	119.5	C17—C16—H16A	120.3
C9—C8—C7	123.2 (4)	C15—C16—H16A	120.3
C9—C8—C3	118.2 (4)	C16—C17—C18	121.3 (6)
C7—C8—C3	118.5 (4)	C16—C17—Cl1	119.5 (5)
C10—C9—C8	121.8 (4)	C18—C17—Cl1	119.2 (5)
C10—C9—H9A	119.1	C17—C18—C19	118.7 (5)
C8—C9—H9A	119.1	C17—C18—H18A	120.6
C9—C10—C1	120.9 (4)	C19—C18—H18A	120.6
C9—C10—H10A	119.5	C14—C19—C18	121.7 (5)
C1—C10—H10A	119.5	C14—C19—H19A	119.1
N1—C11—C1	123.4 (4)	C18—C19—H19A	119.1
			
C10—C1—C2—C3	−2.5 (5)	C12—N1—C11—C1	177.4 (4)
C11—C1—C2—C3	176.0 (3)	C2—C1—C11—N1	−174.8 (4)
C1—C2—C3—C4	−177.3 (3)	C10—C1—C11—N1	3.6 (6)
C1—C2—C3—C8	1.9 (5)	C11—N1—C12—C13	130.7 (5)
C2—C3—C4—C5	177.4 (4)	C11—N1—C12—C14	−104.2 (5)
C8—C3—C4—C5	−1.8 (5)	N1—C12—C14—C19	97.9 (5)
C3—C4—C5—C6	1.4 (6)	C13—C12—C14—C19	−140.5 (5)
C4—C5—C6—C7	−0.4 (6)	N1—C12—C14—C15	−77.9 (6)
C5—C6—C7—C8	−0.3 (6)	C13—C12—C14—C15	43.8 (6)
C6—C7—C8—C9	−177.9 (4)	C19—C14—C15—C16	−0.7 (7)
C6—C7—C8—C3	−0.1 (6)	C12—C14—C15—C16	175.2 (4)
C2—C3—C8—C9	−0.2 (5)	C14—C15—C16—C17	0.3 (8)
C4—C3—C8—C9	179.0 (3)	C15—C16—C17—C18	−0.2 (8)
C2—C3—C8—C7	−178.1 (3)	C15—C16—C17—Cl1	179.7 (4)
C4—C3—C8—C7	1.1 (5)	C16—C17—C18—C19	0.6 (8)
C7—C8—C9—C10	176.9 (4)	Cl1—C17—C18—C19	−179.3 (4)
C3—C8—C9—C10	−0.8 (5)	C15—C14—C19—C18	1.1 (7)
C8—C9—C10—C1	0.2 (6)	C12—C14—C19—C18	−174.9 (4)
C2—C1—C10—C9	1.4 (5)	C17—C18—C19—C14	−1.0 (8)
C11—C1—C10—C9	−177.1 (4)		

### (6) (S)-(+)-2-({[(4-Bromophenyl)ethyl]imino}methyl)naphthalene Crystal data

**Table:**

C_19_H_16_BrN	*D*_x_ = 1.425 Mg m^−^^3^
*M**_r_* = 338.24	Melting point: 415 K
Orthorhombic, *P*2_1_2_1_2_1_	Mo *K*α radiation, λ = 0.71073 Å
*a* = 6.0526 (2) Å	Cell parameters from 4299 reflections
*b* = 7.6671 (4) Å	θ = 3.2–20.7°
*c* = 33.9712 (19) Å	µ = 2.60 mm^−^^1^
*V* = 1576.46 (13) Å^3^	*T* = 298 K
*Z* = 4	Block, colorless
*F*(000) = 688	0.36 × 0.22 × 0.19 mm

### (6) (S)-(+)-2-({[(4-Bromophenyl)ethyl]imino}methyl)naphthalene Data collection

**Table:**

Agilent Xcalibur Atlas Gemini diffractometer	3120 independent reflections
Radiation source: Enhance (Mo) X-ray Source	2017 reflections with *I* > 2σ(*I*)
Graphite monochromator	*R*_int_ = 0.047
Detector resolution: 10.5564 pixels mm^-1^	θ_max_ = 26.1°, θ_min_ = 2.9°
ω scans	*h* = −7→7
Absorption correction: analytical (CrysAlis PRO; Agilent, 2013)	*k* = −9→9
*T*_min_ = 0.876, *T*_max_ = 0.920	*l* = −41→41
19221 measured reflections	

### (6) (S)-(+)-2-({[(4-Bromophenyl)ethyl]imino}methyl)naphthalene Refinement

**Table:**

Refinement on *F*^2^	Secondary atom site location: difference Fourier map
Least-squares matrix: full	Hydrogen site location: inferred from neighbouring sites
*R*[*F*^2^ > 2σ(*F*^2^)] = 0.044	H-atom parameters constrained
*wR*(*F*^2^) = 0.098	*w* = 1/[σ^2^(*F*_o_^2^) + (0.0301*P*)^2^ + 0.937*P*] where *P* = (*F*_o_^2^ + 2*F*_c_^2^)/3
*S* = 1.02	(Δ/σ)_max_ < 0.001
3120 reflections	Δρ_max_ = 0.32 e Å^−^^3^
191 parameters	Δρ_min_ = −0.44 e Å^−^^3^
0 restraints	Absolute structure: Flack *x* determined using 643 quotients [(*I*^+^)-(*I*^-^)]/[(*I*^+^)+(*I*^-^)] (Parsons *et al.*, 2013)
0 constraints	Absolute structure parameter: −0.009 (6)
Primary atom site location: structure-invariant direct methods	

### (6) (S)-(+)-2-({[(4-Bromophenyl)ethyl]imino}methyl)naphthalene Fractional atomic coordinates and isotropic or equivalent isotropic displacement parameters (Å^2^)

**Table:**

	*x*	*y*	*z*	*U*_iso_*/*U*_eq_	
Br1	0.95591 (14)	0.41300 (11)	0.77288 (2)	0.1173 (4)	
N1	0.4062 (7)	−0.1554 (6)	0.88682 (12)	0.0624 (12)	
C1	0.3683 (7)	−0.0914 (6)	0.95548 (13)	0.0437 (11)	
C2	0.4704 (7)	−0.0932 (6)	0.99143 (12)	0.0435 (10)	
H2A	0.6069	−0.1479	0.9938	0.052*	
C3	0.3747 (7)	−0.0144 (5)	1.02523 (13)	0.0433 (11)	
C4	0.4816 (8)	−0.0089 (6)	1.06203 (13)	0.0533 (12)	
H4A	0.6188	−0.0617	1.0651	0.064*	
C5	0.3854 (9)	0.0731 (7)	1.09306 (16)	0.0662 (14)	
H5A	0.4576	0.0759	1.1172	0.079*	
C6	0.1798 (10)	0.1531 (7)	1.08916 (18)	0.0700 (17)	
H6A	0.1173	0.2103	1.1106	0.084*	
C7	0.0695 (9)	0.1482 (6)	1.05408 (16)	0.0601 (14)	
H7A	−0.0686	0.2005	1.0519	0.072*	
C8	0.1636 (7)	0.0644 (6)	1.02110 (14)	0.0438 (11)	
C9	0.0578 (8)	0.0608 (6)	0.98390 (14)	0.0519 (12)	
H9A	−0.0820	0.1095	0.9812	0.062*	
C10	0.1572 (8)	−0.0125 (6)	0.95233 (15)	0.0509 (12)	
H10A	0.0858	−0.0110	0.9281	0.061*	
C11	0.4829 (9)	−0.1642 (6)	0.92147 (14)	0.0529 (12)	
H11A	0.6179	−0.2195	0.9254	0.063*	
C12	0.5447 (10)	−0.2281 (7)	0.85503 (14)	0.0682 (15)	
H12A	0.6637	−0.2967	0.8670	0.082*	
C13	0.4035 (13)	−0.3500 (8)	0.82992 (18)	0.097 (2)	
H13A	0.4890	−0.3915	0.8080	0.145*	
H13B	0.2767	−0.2878	0.8203	0.145*	
H13C	0.3559	−0.4472	0.8456	0.145*	
C14	0.6470 (8)	−0.0754 (8)	0.83342 (13)	0.0575 (13)	
C15	0.8594 (9)	−0.0227 (9)	0.84199 (16)	0.0704 (16)	
H15A	0.9426	−0.0869	0.8599	0.084*	
C16	0.9516 (9)	0.1229 (8)	0.82467 (16)	0.0741 (17)	
H16A	1.0945	0.1574	0.8311	0.089*	
C17	0.8283 (9)	0.2170 (9)	0.79764 (15)	0.0687 (16)	
C18	0.6181 (9)	0.1667 (8)	0.78799 (15)	0.0699 (16)	
H18A	0.5364	0.2299	0.7697	0.084*	
C19	0.5288 (9)	0.0199 (7)	0.80586 (14)	0.0633 (14)	
H19A	0.3866	−0.0154	0.7992	0.076*	

### (6) (S)-(+)-2-({[(4-Bromophenyl)ethyl]imino}methyl)naphthalene Atomic displacement parameters (Å^2^)

**Table:**

	*U*^11^	*U*^22^	*U*^33^	*U*^12^	*U*^13^	*U*^23^
Br1	0.1195 (6)	0.1287 (6)	0.1036 (5)	−0.0465 (5)	0.0041 (5)	0.0162 (5)
N1	0.067 (3)	0.066 (3)	0.055 (3)	0.006 (2)	0.004 (2)	0.007 (2)
C1	0.044 (3)	0.037 (2)	0.050 (3)	−0.001 (2)	−0.001 (2)	0.008 (2)
C2	0.035 (2)	0.035 (2)	0.061 (3)	−0.002 (2)	0.004 (2)	0.009 (2)
C3	0.041 (3)	0.033 (2)	0.056 (3)	−0.005 (2)	0.000 (2)	0.006 (2)
C4	0.055 (3)	0.050 (3)	0.056 (3)	−0.004 (2)	−0.004 (2)	0.003 (2)
C5	0.078 (4)	0.059 (3)	0.062 (3)	−0.004 (3)	0.001 (3)	−0.007 (3)
C6	0.084 (4)	0.053 (3)	0.073 (4)	−0.007 (3)	0.023 (3)	−0.011 (3)
C7	0.051 (3)	0.044 (3)	0.085 (4)	0.004 (2)	0.017 (3)	0.005 (3)
C8	0.039 (2)	0.030 (2)	0.063 (3)	−0.004 (2)	0.001 (2)	0.008 (2)
C9	0.035 (2)	0.043 (3)	0.078 (3)	0.001 (2)	−0.001 (3)	0.013 (2)
C10	0.049 (3)	0.045 (3)	0.059 (3)	0.000 (2)	−0.013 (2)	0.011 (2)
C11	0.060 (3)	0.041 (3)	0.057 (3)	0.004 (2)	−0.001 (3)	0.008 (2)
C12	0.081 (4)	0.068 (4)	0.056 (3)	0.022 (3)	0.001 (3)	−0.002 (3)
C13	0.139 (6)	0.073 (4)	0.079 (4)	−0.013 (4)	0.003 (4)	−0.016 (3)
C14	0.058 (3)	0.073 (3)	0.041 (3)	0.019 (3)	0.000 (2)	−0.007 (3)
C15	0.063 (4)	0.095 (5)	0.053 (3)	0.017 (3)	−0.012 (3)	−0.012 (3)
C16	0.052 (3)	0.106 (5)	0.064 (3)	−0.003 (4)	0.001 (3)	−0.028 (3)
C17	0.065 (3)	0.095 (5)	0.046 (3)	−0.005 (3)	0.005 (3)	−0.012 (3)
C18	0.065 (4)	0.089 (4)	0.056 (3)	0.002 (3)	−0.007 (3)	0.006 (3)
C19	0.050 (3)	0.080 (4)	0.060 (3)	0.004 (3)	−0.005 (3)	0.000 (3)

### (6) (S)-(+)-2-({[(4-Bromophenyl)ethyl]imino}methyl)naphthalene Geometric parameters (Å, º)

**Table:**

Br1—C17	1.887 (6)	C9—H9A	0.9300
N1—C11	1.267 (6)	C10—H10A	0.9300
N1—C12	1.477 (6)	C11—H11A	0.9300
C1—C2	1.369 (6)	C12—C14	1.515 (8)
C1—C10	1.418 (6)	C12—C13	1.527 (8)
C1—C11	1.459 (6)	C12—H12A	0.9800
C2—C3	1.421 (6)	C13—H13A	0.9600
C2—H2A	0.9300	C13—H13B	0.9600
C3—C4	1.408 (6)	C13—H13C	0.9600
C3—C8	1.420 (6)	C14—C15	1.379 (7)
C4—C5	1.358 (7)	C14—C19	1.386 (7)
C4—H4A	0.9300	C15—C16	1.380 (8)
C5—C6	1.393 (8)	C15—H15A	0.9300
C5—H5A	0.9300	C16—C17	1.386 (8)
C6—C7	1.366 (7)	C16—H16A	0.9300
C6—H6A	0.9300	C17—C18	1.369 (8)
C7—C8	1.412 (7)	C18—C19	1.388 (8)
C7—H7A	0.9300	C18—H18A	0.9300
C8—C9	1.417 (6)	C19—H19A	0.9300
C9—C10	1.352 (6)		
			
C11—N1—C12	116.8 (5)	N1—C11—H11A	118.6
C2—C1—C10	118.6 (4)	C1—C11—H11A	118.6
C2—C1—C11	119.2 (4)	N1—C12—C14	107.1 (4)
C10—C1—C11	122.2 (4)	N1—C12—C13	108.8 (5)
C1—C2—C3	122.2 (4)	C14—C12—C13	115.5 (5)
C1—C2—H2A	118.9	N1—C12—H12A	108.4
C3—C2—H2A	118.9	C14—C12—H12A	108.4
C4—C3—C8	119.2 (4)	C13—C12—H12A	108.4
C4—C3—C2	122.9 (4)	C12—C13—H13A	109.5
C8—C3—C2	117.9 (4)	C12—C13—H13B	109.5
C5—C4—C3	120.4 (5)	H13A—C13—H13B	109.5
C5—C4—H4A	119.8	C12—C13—H13C	109.5
C3—C4—H4A	119.8	H13A—C13—H13C	109.5
C4—C5—C6	120.8 (5)	H13B—C13—H13C	109.5
C4—C5—H5A	119.6	C15—C14—C19	118.0 (6)
C6—C5—H5A	119.6	C15—C14—C12	120.4 (5)
C7—C6—C5	120.5 (5)	C19—C14—C12	121.6 (5)
C7—C6—H6A	119.8	C14—C15—C16	121.6 (6)
C5—C6—H6A	119.8	C14—C15—H15A	119.2
C6—C7—C8	120.5 (5)	C16—C15—H15A	119.2
C6—C7—H7A	119.8	C15—C16—C17	119.1 (5)
C8—C7—H7A	119.8	C15—C16—H16A	120.5
C7—C8—C9	122.3 (4)	C17—C16—H16A	120.5
C7—C8—C3	118.6 (4)	C18—C17—C16	120.8 (6)
C9—C8—C3	119.1 (4)	C18—C17—Br1	119.9 (5)
C10—C9—C8	121.0 (4)	C16—C17—Br1	119.3 (4)
C10—C9—H9A	119.5	C17—C18—C19	119.1 (6)
C8—C9—H9A	119.5	C17—C18—H18A	120.5
C9—C10—C1	121.2 (4)	C19—C18—H18A	120.5
C9—C10—H10A	119.4	C14—C19—C18	121.4 (5)
C1—C10—H10A	119.4	C14—C19—H19A	119.3
N1—C11—C1	122.7 (5)	C18—C19—H19A	119.3
			
C10—C1—C2—C3	−1.9 (7)	C12—N1—C11—C1	177.4 (5)
C11—C1—C2—C3	175.8 (4)	C2—C1—C11—N1	−174.6 (5)
C1—C2—C3—C4	−177.3 (4)	C10—C1—C11—N1	3.0 (7)
C1—C2—C3—C8	1.5 (6)	C11—N1—C12—C14	−104.2 (5)
C8—C3—C4—C5	−1.0 (7)	C11—N1—C12—C13	130.3 (5)
C2—C3—C4—C5	177.9 (5)	N1—C12—C14—C15	98.1 (6)
C3—C4—C5—C6	0.0 (8)	C13—C12—C14—C15	−140.6 (6)
C4—C5—C6—C7	1.0 (8)	N1—C12—C14—C19	−79.2 (6)
C5—C6—C7—C8	−1.0 (8)	C13—C12—C14—C19	42.1 (7)
C6—C7—C8—C9	−178.1 (5)	C19—C14—C15—C16	1.7 (8)
C6—C7—C8—C3	0.0 (7)	C12—C14—C15—C16	−175.7 (5)
C4—C3—C8—C7	0.9 (6)	C14—C15—C16—C17	−0.8 (8)
C2—C3—C8—C7	−178.0 (4)	C15—C16—C17—C18	−0.2 (8)
C4—C3—C8—C9	179.1 (4)	C15—C16—C17—Br1	−178.5 (4)
C2—C3—C8—C9	0.2 (6)	C16—C17—C18—C19	0.3 (8)
C7—C8—C9—C10	176.5 (5)	Br1—C17—C18—C19	178.6 (4)
C3—C8—C9—C10	−1.7 (7)	C15—C14—C19—C18	−1.6 (7)
C8—C9—C10—C1	1.4 (7)	C12—C14—C19—C18	175.7 (5)
C2—C1—C10—C9	0.4 (7)	C17—C18—C19—C14	0.6 (8)
C11—C1—C10—C9	−177.2 (4)		

### (7) (S)-(+)-2-({[1-(Naphthalen-1-yl)ethyl]imino}methyl)naphthalene Crystal data

**Table:**

C_23_H_19_N	*D*_x_ = 1.200 Mg m^−^^3^
*M**_r_* = 309.39	Melting point: 404 K
Monoclinic, *P*2_1_	Mo *K*α radiation, λ = 0.71073 Å
*a* = 7.8555 (5) Å	Cell parameters from 1835 reflections
*b* = 7.8724 (4) Å	θ = 3.6–22.1°
*c* = 14.0494 (9) Å	µ = 0.07 mm^−^^1^
β = 99.859 (6)°	*T* = 298 K
*V* = 856.01 (9) Å^3^	Prism, colorless
*Z* = 2	0.45 × 0.35 × 0.11 mm
*F*(000) = 328	

### (7) (S)-(+)-2-({[1-(Naphthalen-1-yl)ethyl]imino}methyl)naphthalene Data collection

**Table:**

Agilent Xcalibur Atlas Gemini diffractometer	3367 independent reflections
Radiation source: Enhance (Mo) X-ray Source	2063 reflections with *I* > 2σ(*I*)
Graphite monochromator	*R*_int_ = 0.043
Detector resolution: 10.5564 pixels mm^-1^	θ_max_ = 26.4°, θ_min_ = 2.9°
ω scans	*h* = −9→9
Absorption correction: multi-scan (CrysAlis PRO; Agilent, 2013)	*k* = −9→9
*T*_min_ = 0.921, *T*_max_ = 1.000	*l* = −17→17
9508 measured reflections	

### (7) (S)-(+)-2-({[1-(Naphthalen-1-yl)ethyl]imino}methyl)naphthalene Refinement

**Table:**

Refinement on *F*^2^	Primary atom site location: structure-invariant direct methods
Least-squares matrix: full	Secondary atom site location: difference Fourier map
*R*[*F*^2^ > 2σ(*F*^2^)] = 0.048	Hydrogen site location: inferred from neighbouring sites
*wR*(*F*^2^) = 0.119	H-atom parameters constrained
*S* = 1.01	*w* = 1/[σ^2^(*F*_o_^2^) + (0.0473*P*)^2^] where *P* = (*F*_o_^2^ + 2*F*_c_^2^)/3
3367 reflections	(Δ/σ)_max_ < 0.001
218 parameters	Δρ_max_ = 0.12 e Å^−^^3^
1 restraint	Δρ_min_ = −0.14 e Å^−^^3^
0 constraints	

### (7) (S)-(+)-2-({[1-(Naphthalen-1-yl)ethyl]imino}methyl)naphthalene Fractional atomic coordinates and isotropic or equivalent isotropic displacement parameters (Å^2^)

**Table:**

	*x*	*y*	*z*	*U*_iso_*/*U*_eq_	
N1	−0.1489 (4)	0.3645 (4)	0.7552 (2)	0.0594 (8)	
C1	0.0105 (4)	0.3346 (4)	0.6238 (2)	0.0533 (9)	
C2	0.0180 (5)	0.3772 (5)	0.5300 (3)	0.0600 (9)	
H2A	−0.0747	0.4341	0.4937	0.072*	
C3	0.1634 (5)	0.3367 (4)	0.4871 (3)	0.0579 (9)	
C4	0.1732 (6)	0.3813 (5)	0.3899 (3)	0.0744 (11)	
H4A	0.0820	0.4388	0.3525	0.089*	
C5	0.3159 (7)	0.3397 (5)	0.3515 (3)	0.0840 (13)	
H5A	0.3211	0.3690	0.2879	0.101*	
C6	0.4533 (6)	0.2545 (6)	0.4061 (4)	0.0841 (13)	
H6A	0.5506	0.2295	0.3793	0.101*	
C7	0.4475 (5)	0.2068 (5)	0.4987 (3)	0.0723 (11)	
H7A	0.5400	0.1479	0.5340	0.087*	
C8	0.3019 (4)	0.2462 (4)	0.5417 (3)	0.0560 (9)	
C9	0.2885 (5)	0.1980 (5)	0.6371 (3)	0.0644 (10)	
H9A	0.3766	0.1346	0.6732	0.077*	
C10	0.1488 (5)	0.2429 (5)	0.6772 (3)	0.0601 (10)	
H10A	0.1447	0.2125	0.7408	0.072*	
C11	−0.1433 (5)	0.3774 (5)	0.6659 (3)	0.0572 (9)	
H11A	−0.2413	0.4159	0.6248	0.069*	
C12	−0.3172 (5)	0.4008 (4)	0.7839 (3)	0.0557 (9)	
H12A	−0.4047	0.4169	0.7259	0.067*	
C13	−0.3647 (5)	0.2462 (5)	0.8398 (3)	0.0716 (11)	
H13A	−0.4768	0.2630	0.8568	0.107*	
H13B	−0.2807	0.2320	0.8975	0.107*	
H13C	−0.3665	0.1466	0.8002	0.107*	
C14	−0.3091 (4)	0.5589 (4)	0.8471 (2)	0.0498 (9)	
C15	−0.4631 (5)	0.6534 (4)	0.8513 (3)	0.0513 (9)	
C16	−0.6267 (5)	0.6113 (5)	0.7974 (3)	0.0594 (10)	
H16A	−0.6371	0.5155	0.7581	0.071*	
C17	−0.7704 (5)	0.7080 (6)	0.8015 (3)	0.0720 (11)	
H17A	−0.8762	0.6774	0.7651	0.086*	
C18	−0.7582 (6)	0.8529 (6)	0.8604 (3)	0.0733 (12)	
H18A	−0.8554	0.9194	0.8622	0.088*	
C19	−0.6045 (6)	0.8956 (5)	0.9146 (3)	0.0683 (11)	
H19A	−0.5981	0.9906	0.9544	0.082*	
C20	−0.4538 (5)	0.7991 (4)	0.9120 (3)	0.0548 (9)	
C21	−0.2945 (6)	0.8411 (5)	0.9699 (3)	0.0693 (11)	
H21A	−0.2880	0.9335	1.0117	0.083*	
C22	−0.1513 (5)	0.7487 (5)	0.9655 (3)	0.0721 (11)	
H22A	−0.0473	0.7786	1.0041	0.086*	
C23	−0.1572 (5)	0.6078 (5)	0.9034 (3)	0.0612 (10)	
H23A	−0.0566	0.5471	0.9005	0.073*	

### (7) (S)-(+)-2-({[1-(Naphthalen-1-yl)ethyl]imino}methyl)naphthalene Atomic displacement parameters (Å^2^)

**Table:**

	*U*^11^	*U*^22^	*U*^33^	*U*^12^	*U*^13^	*U*^23^
N1	0.0636 (19)	0.0600 (19)	0.0557 (19)	0.0067 (16)	0.0132 (15)	−0.0052 (16)
C1	0.055 (2)	0.050 (2)	0.054 (2)	0.0035 (17)	0.0075 (18)	−0.0057 (17)
C2	0.066 (2)	0.055 (2)	0.058 (2)	0.011 (2)	0.0069 (19)	0.0004 (19)
C3	0.072 (3)	0.046 (2)	0.057 (2)	−0.0050 (19)	0.016 (2)	−0.0079 (18)
C4	0.103 (3)	0.057 (2)	0.067 (3)	0.006 (2)	0.024 (2)	−0.003 (2)
C5	0.121 (4)	0.061 (3)	0.080 (3)	−0.006 (3)	0.045 (3)	−0.010 (2)
C6	0.093 (3)	0.070 (3)	0.100 (4)	−0.008 (3)	0.048 (3)	−0.024 (3)
C7	0.066 (3)	0.065 (3)	0.087 (3)	0.001 (2)	0.018 (2)	−0.019 (2)
C8	0.059 (2)	0.0451 (19)	0.064 (2)	0.0001 (18)	0.0118 (19)	−0.0117 (18)
C9	0.064 (3)	0.058 (2)	0.068 (3)	0.007 (2)	0.001 (2)	−0.010 (2)
C10	0.068 (2)	0.057 (2)	0.054 (2)	0.000 (2)	0.0061 (19)	−0.0075 (19)
C11	0.059 (2)	0.050 (2)	0.062 (2)	0.0047 (18)	0.0078 (19)	−0.0023 (19)
C12	0.060 (2)	0.051 (2)	0.059 (2)	0.0008 (17)	0.0151 (18)	−0.0023 (18)
C13	0.085 (3)	0.052 (2)	0.079 (3)	−0.009 (2)	0.016 (2)	−0.002 (2)
C14	0.056 (2)	0.047 (2)	0.048 (2)	−0.0038 (17)	0.0130 (17)	0.0013 (16)
C15	0.063 (2)	0.045 (2)	0.050 (2)	−0.0091 (17)	0.0208 (17)	0.0036 (17)
C16	0.065 (3)	0.056 (2)	0.061 (2)	0.000 (2)	0.021 (2)	0.0025 (18)
C17	0.062 (3)	0.079 (3)	0.077 (3)	−0.002 (2)	0.019 (2)	0.006 (2)
C18	0.072 (3)	0.067 (3)	0.088 (3)	0.014 (2)	0.034 (2)	0.009 (3)
C19	0.086 (3)	0.054 (2)	0.072 (3)	−0.001 (2)	0.035 (2)	−0.001 (2)
C20	0.062 (2)	0.048 (2)	0.057 (2)	−0.0079 (18)	0.0213 (19)	0.0043 (17)
C21	0.085 (3)	0.055 (2)	0.072 (3)	−0.012 (2)	0.024 (2)	−0.011 (2)
C22	0.074 (3)	0.067 (3)	0.074 (3)	−0.020 (2)	0.006 (2)	−0.010 (2)
C23	0.063 (2)	0.058 (2)	0.064 (3)	0.000 (2)	0.012 (2)	0.002 (2)

### (7) (S)-(+)-2-({[1-(Naphthalen-1-yl)ethyl]imino}methyl)naphthalene Geometric parameters (Å, º)

**Table:**

N1—C11	1.268 (4)	C12—C13	1.529 (5)
N1—C12	1.474 (4)	C12—H12A	0.9800
C1—C2	1.371 (5)	C13—H13A	0.9600
C1—C10	1.409 (5)	C13—H13B	0.9600
C1—C11	1.472 (5)	C13—H13C	0.9600
C2—C3	1.416 (5)	C14—C23	1.369 (5)
C2—H2A	0.9300	C14—C15	1.430 (5)
C3—C8	1.413 (5)	C15—C16	1.415 (5)
C3—C4	1.425 (5)	C15—C20	1.424 (4)
C4—C5	1.365 (6)	C16—C17	1.370 (5)
C4—H4A	0.9300	C16—H16A	0.9300
C5—C6	1.386 (6)	C17—C18	1.403 (6)
C5—H5A	0.9300	C17—H17A	0.9300
C6—C7	1.362 (6)	C18—C19	1.355 (5)
C6—H6A	0.9300	C18—H18A	0.9300
C7—C8	1.417 (5)	C19—C20	1.412 (5)
C7—H7A	0.9300	C19—H19A	0.9300
C8—C9	1.414 (5)	C20—C21	1.411 (5)
C9—C10	1.363 (5)	C21—C22	1.350 (5)
C9—H9A	0.9300	C21—H21A	0.9300
C10—H10A	0.9300	C22—C23	1.407 (5)
C11—H11A	0.9300	C22—H22A	0.9300
C12—C14	1.524 (5)	C23—H23A	0.9300
			
C11—N1—C12	116.1 (3)	C14—C12—H12A	109.4
C2—C1—C10	118.8 (3)	C13—C12—H12A	109.4
C2—C1—C11	120.2 (3)	C12—C13—H13A	109.5
C10—C1—C11	121.0 (3)	C12—C13—H13B	109.5
C1—C2—C3	121.7 (3)	H13A—C13—H13B	109.5
C1—C2—H2A	119.2	C12—C13—H13C	109.5
C3—C2—H2A	119.2	H13A—C13—H13C	109.5
C8—C3—C2	119.0 (3)	H13B—C13—H13C	109.5
C8—C3—C4	118.8 (4)	C23—C14—C15	119.3 (3)
C2—C3—C4	122.2 (4)	C23—C14—C12	120.8 (3)
C5—C4—C3	120.2 (4)	C15—C14—C12	119.8 (3)
C5—C4—H4A	119.9	C16—C15—C20	117.3 (3)
C3—C4—H4A	119.9	C16—C15—C14	123.6 (3)
C4—C5—C6	120.8 (4)	C20—C15—C14	119.1 (3)
C4—C5—H5A	119.6	C17—C16—C15	121.8 (4)
C6—C5—H5A	119.6	C17—C16—H16A	119.1
C7—C6—C5	120.9 (4)	C15—C16—H16A	119.1
C7—C6—H6A	119.6	C16—C17—C18	120.2 (4)
C5—C6—H6A	119.6	C16—C17—H17A	119.9
C6—C7—C8	120.6 (4)	C18—C17—H17A	119.9
C6—C7—H7A	119.7	C19—C18—C17	119.8 (4)
C8—C7—H7A	119.7	C19—C18—H18A	120.1
C3—C8—C9	118.4 (3)	C17—C18—H18A	120.1
C3—C8—C7	118.8 (4)	C18—C19—C20	121.4 (4)
C9—C8—C7	122.8 (4)	C18—C19—H19A	119.3
C10—C9—C8	121.2 (4)	C20—C19—H19A	119.3
C10—C9—H9A	119.4	C21—C20—C19	121.6 (4)
C8—C9—H9A	119.4	C21—C20—C15	119.0 (3)
C9—C10—C1	121.0 (3)	C19—C20—C15	119.5 (3)
C9—C10—H10A	119.5	C22—C21—C20	120.7 (4)
C1—C10—H10A	119.5	C22—C21—H21A	119.6
N1—C11—C1	123.3 (3)	C20—C21—H21A	119.6
N1—C11—H11A	118.3	C21—C22—C23	120.9 (4)
C1—C11—H11A	118.3	C21—C22—H22A	119.5
N1—C12—C14	111.6 (3)	C23—C22—H22A	119.5
N1—C12—C13	107.2 (3)	C14—C23—C22	120.9 (4)
C14—C12—C13	109.9 (3)	C14—C23—H23A	119.6
N1—C12—H12A	109.4	C22—C23—H23A	119.6
			
C10—C1—C2—C3	−2.3 (5)	N1—C12—C14—C23	−27.4 (4)
C11—C1—C2—C3	−179.5 (3)	C13—C12—C14—C23	91.3 (4)
C1—C2—C3—C8	1.7 (5)	N1—C12—C14—C15	155.4 (3)
C1—C2—C3—C4	−179.6 (3)	C13—C12—C14—C15	−85.9 (4)
C8—C3—C4—C5	−1.3 (5)	C23—C14—C15—C16	−178.8 (3)
C2—C3—C4—C5	180.0 (3)	C12—C14—C15—C16	−1.6 (5)
C3—C4—C5—C6	−0.2 (6)	C23—C14—C15—C20	1.7 (5)
C4—C5—C6—C7	1.5 (6)	C12—C14—C15—C20	179.0 (3)
C5—C6—C7—C8	−1.2 (6)	C20—C15—C16—C17	1.2 (5)
C2—C3—C8—C9	0.7 (5)	C14—C15—C16—C17	−178.2 (3)
C4—C3—C8—C9	−178.1 (3)	C15—C16—C17—C18	−0.2 (5)
C2—C3—C8—C7	−179.6 (3)	C16—C17—C18—C19	−1.1 (6)
C4—C3—C8—C7	1.6 (5)	C17—C18—C19—C20	1.3 (6)
C6—C7—C8—C3	−0.4 (5)	C18—C19—C20—C21	−178.6 (4)
C6—C7—C8—C9	179.3 (4)	C18—C19—C20—C15	−0.2 (5)
C3—C8—C9—C10	−2.5 (5)	C16—C15—C20—C21	177.4 (3)
C7—C8—C9—C10	177.9 (4)	C14—C15—C20—C21	−3.1 (4)
C8—C9—C10—C1	1.9 (5)	C16—C15—C20—C19	−1.1 (4)
C2—C1—C10—C9	0.5 (5)	C14—C15—C20—C19	178.4 (3)
C11—C1—C10—C9	177.7 (3)	C19—C20—C21—C22	−179.1 (3)
C12—N1—C11—C1	−175.7 (3)	C15—C20—C21—C22	2.4 (5)
C2—C1—C11—N1	−169.3 (3)	C20—C21—C22—C23	−0.3 (6)
C10—C1—C11—N1	13.5 (5)	C15—C14—C23—C22	0.5 (5)
C11—N1—C12—C14	−114.4 (3)	C12—C14—C23—C22	−176.8 (3)
C11—N1—C12—C13	125.3 (3)	C21—C22—C23—C14	−1.2 (6)

### (8) (S)-(+)-2-{[(1-Cyclohexylethyl)imino]methyl}naphthalene Crystal data

**Table:**

C_19_H_23_N	*D*_x_ = 1.088 Mg m^−^^3^
*M**_r_* = 265.38	Melting point: 356 K
Monoclinic, *P*2_1_	Mo *K*α radiation, λ = 0.71073 Å
*a* = 15.406 (3) Å	Cell parameters from 2217 reflections
*b* = 5.9722 (7) Å	θ = 3.5–22.2°
*c* = 36.002 (7) Å	µ = 0.06 mm^−^^1^
β = 102.058 (18)°	*T* = 298 K
*V* = 3239.4 (10) Å^3^	Prism, colorless
*Z* = 8	0.54 × 0.09 × 0.07 mm
*F*(000) = 1152	

### (8) (S)-(+)-2-{[(1-Cyclohexylethyl)imino]methyl}naphthalene Data collection

**Table:**

Agilent Xcalibur Atlas Gemini diffractometer	10600 independent reflections
Radiation source: Enhance (Mo) X-ray Source	3810 reflections with *I* > 2σ(*I*)
Graphite monochromator	*R*_int_ = 0.119
Detector resolution: 10.5564 pixels mm^-1^	θ_max_ = 25.0°, θ_min_ = 2.9°
ω scans	*h* = −18→18
Absorption correction: analytical (CrysAlis PRO; Agilent, 2013)	*k* = −7→6
*T*_min_ = 0.995, *T*_max_ = 0.999	*l* = −42→42
19804 measured reflections	

### (8) (S)-(+)-2-{[(1-Cyclohexylethyl)imino]methyl}naphthalene Refinement

**Table:**

Refinement on *F*^2^	Secondary atom site location: difference Fourier map
Least-squares matrix: full	Hydrogen site location: inferred from neighbouring sites
*R*[*F*^2^ > 2σ(*F*^2^)] = 0.078	H-atom parameters constrained
*wR*(*F*^2^) = 0.231	*w* = 1/[σ^2^(*F*_o_^2^) + (0.050*P*)^2^] where *P* = (*F*_o_^2^ + 2*F*_c_^2^)/3
*S* = 0.99	(Δ/σ)_max_ < 0.001
10600 reflections	Δρ_max_ = 0.23 e Å^−^^3^
727 parameters	Δρ_min_ = −0.18 e Å^−^^3^
37 restraints	Extinction correction: SHELXL2014 (Sheldrick, 2015), Fc^*^=kFc[1+0.001xFc^2^λ^3^/sin(2θ)]^-1/4^
0 constraints	Extinction coefficient: 0.0043 (8)
Primary atom site location: structure-invariant direct methods	

### (8) (S)-(+)-2-{[(1-Cyclohexylethyl)imino]methyl}naphthalene Special details

**Table:**

Refinement. Refined as a 2-component twin.

### (8) (S)-(+)-2-{[(1-Cyclohexylethyl)imino]methyl}naphthalene Fractional atomic coordinates and isotropic or equivalent isotropic displacement parameters (Å^2^)

**Table:**

	*x*	*y*	*z*	*U*_iso_*/*U*_eq_	
N1	0.3854 (7)	0.2863 (18)	0.1115 (3)	0.111 (3)	
C1	0.3747 (6)	0.3568 (16)	0.0448 (3)	0.076 (3)	
C2	0.3473 (6)	0.5063 (18)	0.0164 (4)	0.085 (3)	
H2A	0.3249	0.6442	0.0219	0.102*	
C3	0.3526 (6)	0.4535 (15)	−0.0220 (3)	0.068 (3)	
C4	0.3261 (7)	0.6069 (17)	−0.0510 (4)	0.087 (3)	
H4A	0.3018	0.7437	−0.0463	0.105*	
C5	0.3365 (7)	0.5529 (19)	−0.0873 (4)	0.105 (4)	
H5A	0.3144	0.6510	−0.1071	0.125*	
C6	0.3805 (7)	0.3490 (19)	−0.0958 (4)	0.102 (4)	
H6A	0.3935	0.3208	−0.1195	0.122*	
C7	0.4012 (7)	0.2032 (18)	−0.0662 (4)	0.088 (3)	
H7A	0.4240	0.0650	−0.0713	0.105*	
C8	0.3917 (6)	0.2399 (16)	−0.0296 (4)	0.080 (3)	
C9	0.4174 (6)	0.0945 (17)	0.0017 (4)	0.090 (3)	
H9A	0.4398	−0.0459	−0.0024	0.108*	
C10	0.4109 (7)	0.1511 (17)	0.0375 (4)	0.092 (4)	
H10A	0.4307	0.0519	0.0574	0.110*	
C11	0.3688 (7)	0.420 (2)	0.0843 (4)	0.096 (4)	
H11A	0.3520	0.5655	0.0888	0.116*	
C12	0.3782 (10)	0.371 (3)	0.1491 (4)	0.132 (5)	
H12A	0.3747	0.5348	0.1476	0.158*	
C13	0.2921 (11)	0.286 (4)	0.1584 (4)	0.212 (9)	
H13A	0.2734	0.3855	0.1761	0.318*	
H13B	0.3016	0.1390	0.1694	0.318*	
H13C	0.2471	0.2783	0.1355	0.318*	
C14	0.4604 (10)	0.309 (3)	0.1801 (5)	0.169 (7)	
H14A	0.4526	0.3827	0.2035	0.203*	
C15	0.4727 (13)	0.079 (4)	0.1887 (7)	0.232 (11)	
H15A	0.4847	−0.0002	0.1668	0.279*	
H15B	0.4191	0.0173	0.1948	0.279*	
C16	0.5527 (16)	0.047 (4)	0.2235 (6)	0.253 (12)	
H16A	0.5425	0.1323	0.2451	0.304*	
H16B	0.5588	−0.1096	0.2306	0.304*	
C17	0.6350 (13)	0.128 (4)	0.2118 (8)	0.270 (15)	
H17A	0.6872	0.0980	0.2315	0.325*	
H17B	0.6421	0.0564	0.1884	0.325*	
C18	0.6212 (15)	0.369 (5)	0.2063 (7)	0.309 (18)	
H18A	0.6742	0.4394	0.2012	0.371*	
H18B	0.6076	0.4368	0.2289	0.371*	
C19	0.5404 (13)	0.401 (4)	0.1711 (5)	0.279 (16)	
H19A	0.5319	0.5589	0.1652	0.335*	
H19B	0.5532	0.3256	0.1490	0.335*	
N2	0.2748 (6)	0.8298 (13)	0.4005 (3)	0.086 (3)	
C21	0.3327 (6)	0.7862 (15)	0.4684 (3)	0.065 (2)	
C22	0.3372 (5)	0.6515 (14)	0.4990 (3)	0.064 (2)	
H22A	0.3070	0.5158	0.4961	0.076*	
C23	0.3858 (6)	0.7105 (13)	0.5346 (3)	0.059 (2)	
C24	0.3952 (6)	0.5701 (16)	0.5669 (3)	0.076 (3)	
H24A	0.3667	0.4321	0.5648	0.092*	
C25	0.4457 (7)	0.6350 (18)	0.6011 (3)	0.090 (3)	
H25A	0.4502	0.5419	0.6221	0.108*	
C26	0.4903 (7)	0.8380 (19)	0.6048 (3)	0.098 (4)	
H26A	0.5243	0.8796	0.6283	0.118*	
C27	0.4848 (7)	0.9771 (17)	0.5744 (3)	0.083 (3)	
H27A	0.5159	1.1116	0.5773	0.100*	
C28	0.4331 (6)	0.9207 (15)	0.5388 (3)	0.065 (2)	
C29	0.4260 (6)	1.0599 (15)	0.5063 (3)	0.072 (3)	
H29A	0.4544	1.1981	0.5086	0.086*	
C30	0.3785 (6)	0.9945 (15)	0.4719 (3)	0.069 (3)	
H30A	0.3760	1.0852	0.4507	0.083*	
C31	0.2838 (6)	0.7128 (16)	0.4307 (3)	0.072 (3)	
H31A	0.2577	0.5716	0.4288	0.087*	
C32	0.2259 (7)	0.7339 (18)	0.3644 (3)	0.088 (3)	
H32A	0.2038	0.5852	0.3693	0.105*	
C33	0.1462 (7)	0.887 (2)	0.3490 (3)	0.112 (4)	
H33A	0.1144	0.8307	0.3250	0.168*	
H33B	0.1670	1.0358	0.3456	0.168*	
H33C	0.1075	0.8907	0.3667	0.168*	
C34	0.2894 (7)	0.7096 (17)	0.3371 (3)	0.094 (3)	
H34A	0.3399	0.6220	0.3508	0.113*	
C35	0.2493 (10)	0.574 (2)	0.3022 (3)	0.127 (5)	
H35A	0.2276	0.4326	0.3099	0.152*	
H35B	0.1993	0.6548	0.2873	0.152*	
C36	0.3181 (11)	0.530 (3)	0.2777 (4)	0.153 (6)	
H36A	0.3652	0.4365	0.2918	0.184*	
H36B	0.2901	0.4492	0.2550	0.184*	
C37	0.3565 (10)	0.740 (3)	0.2669 (4)	0.140 (5)	
H37A	0.3105	0.8258	0.2504	0.168*	
H37B	0.4020	0.7054	0.2528	0.168*	
C38	0.3964 (8)	0.880 (2)	0.3010 (4)	0.125 (4)	
H38A	0.4159	1.0226	0.2928	0.150*	
H38B	0.4478	0.8039	0.3158	0.150*	
C39	0.3272 (7)	0.9212 (18)	0.3261 (3)	0.103 (4)	
H39A	0.3554	1.0015	0.3489	0.123*	
H39B	0.2797	1.0142	0.3123	0.123*	
N3	0.7594 (6)	0.6491 (16)	0.3782 (3)	0.102 (3)	
C41	0.8088 (6)	0.7277 (15)	0.4437 (3)	0.066 (2)	
C42	0.8160 (5)	0.6512 (15)	0.4807 (3)	0.066 (2)	
H42A	0.7902	0.5153	0.4849	0.079*	
C43	0.8616 (5)	0.7762 (15)	0.5118 (3)	0.062 (2)	
C44	0.8733 (6)	0.6999 (16)	0.5498 (3)	0.073 (3)	
H44A	0.8486	0.5632	0.5544	0.088*	
C45	0.9190 (7)	0.8190 (18)	0.5796 (3)	0.083 (3)	
H45A	0.9259	0.7637	0.6042	0.100*	
C46	0.9567 (7)	1.0315 (19)	0.5729 (3)	0.087 (3)	
H46A	0.9873	1.1155	0.5932	0.104*	
C47	0.9472 (6)	1.1102 (16)	0.5363 (3)	0.080 (3)	
H47A	0.9722	1.2472	0.5321	0.096*	
C48	0.9004 (6)	0.9874 (15)	0.5052 (3)	0.065 (2)	
C49	0.8878 (6)	1.0654 (14)	0.4677 (3)	0.072 (3)	
H49A	0.9093	1.2061	0.4631	0.086*	
C50	0.8448 (6)	0.9397 (15)	0.4376 (3)	0.074 (3)	
H50A	0.8391	0.9939	0.4130	0.089*	
C51	0.7641 (6)	0.5892 (18)	0.4119 (3)	0.077 (3)	
H51A	0.7387	0.4540	0.4168	0.093*	
C52	0.7134 (8)	0.504 (2)	0.3469 (3)	0.104 (4)	
H52A	0.6936	0.3677	0.3577	0.125*	
C53	0.6334 (9)	0.625 (3)	0.3245 (4)	0.183 (8)	
H53A	0.6089	0.5400	0.3021	0.275*	
H53B	0.6507	0.7701	0.3173	0.275*	
H53C	0.5896	0.6403	0.3398	0.275*	
C54	0.7803 (8)	0.4390 (19)	0.3213 (3)	0.090 (3)	
H54A	0.7464	0.3596	0.2992	0.108*	
C55	0.8201 (12)	0.637 (2)	0.3071 (4)	0.145 (6)	
H55A	0.8519	0.7249	0.3283	0.174*	
H55B	0.7739	0.7301	0.2923	0.174*	
C56	0.8848 (15)	0.561 (3)	0.2820 (4)	0.167 (8)	
H56A	0.8526	0.4780	0.2603	0.201*	
H56B	0.9116	0.6907	0.2727	0.201*	
C57	0.9570 (11)	0.413 (3)	0.3052 (5)	0.165 (7)	
H57A	0.9997	0.3715	0.2901	0.198*	
H57B	0.9881	0.4933	0.3275	0.198*	
C58	0.9152 (9)	0.212 (3)	0.3168 (4)	0.127 (5)	
H58A	0.9601	0.1134	0.3311	0.152*	
H58B	0.8840	0.1320	0.2945	0.152*	
C59	0.8498 (7)	0.282 (2)	0.3414 (3)	0.103 (4)	
H59A	0.8213	0.1488	0.3488	0.124*	
H59B	0.8823	0.3526	0.3643	0.124*	
N4	0.8847 (6)	0.5784 (17)	0.1214 (3)	0.104 (3)	
C61	0.8680 (6)	0.4718 (17)	0.0557 (3)	0.079 (3)	
C62	0.8375 (6)	0.5241 (16)	0.0182 (3)	0.073 (3)	
H62A	0.8072	0.6587	0.0125	0.087*	
C63	0.8489 (6)	0.3873 (15)	−0.0124 (3)	0.070 (3)	
C64	0.8195 (7)	0.4542 (19)	−0.0494 (4)	0.092 (3)	
H64A	0.7905	0.5905	−0.0550	0.110*	
C65	0.8339 (8)	0.315 (2)	−0.0779 (4)	0.104 (4)	
H65A	0.8128	0.3554	−0.1032	0.125*	
C66	0.8806 (8)	0.110 (2)	−0.0693 (4)	0.108 (4)	
H66A	0.8927	0.0210	−0.0888	0.130*	
C67	0.9071 (7)	0.0467 (19)	−0.0331 (4)	0.093 (3)	
H67A	0.9347	−0.0915	−0.0278	0.112*	
C68	0.8941 (6)	0.1839 (17)	−0.0029 (4)	0.079 (3)	
C69	0.9230 (7)	0.1238 (17)	0.0361 (4)	0.089 (3)	
H69A	0.9506	−0.0138	0.0422	0.106*	
C70	0.9113 (7)	0.2626 (17)	0.0648 (3)	0.084 (3)	
H70A	0.9315	0.2209	0.0900	0.101*	
C71	0.8551 (7)	0.624 (2)	0.0865 (4)	0.092 (3)	
H71A	0.8245	0.7575	0.0802	0.110*	
C72	0.8708 (9)	0.754 (2)	0.1492 (4)	0.113 (4)	
H72A	0.8441	0.8868	0.1354	0.136*	
C73	0.8078 (11)	0.656 (3)	0.1714 (4)	0.163 (6)	
H73A	0.7531	0.6177	0.1544	0.245*	
H73B	0.7965	0.7635	0.1896	0.245*	
H73C	0.8335	0.5236	0.1844	0.245*	
C74	0.9592 (10)	0.816 (3)	0.1747 (5)	0.163 (7)	
H74A	0.9620	0.7059	0.1951	0.195*	
C75	0.9530 (11)	1.028 (3)	0.1957 (5)	0.179 (7)	
H75A	0.9453	1.1524	0.1780	0.215*	
H75B	0.9015	1.0218	0.2072	0.215*	
C76	1.0366 (15)	1.065 (5)	0.2265 (7)	0.305 (18)	
H76A	1.0309	1.2038	0.2397	0.366*	
H76B	1.0424	0.9439	0.2449	0.366*	
C77	1.1135 (16)	1.074 (3)	0.2111 (6)	0.253 (12)	
H77A	1.1651	1.0993	0.2313	0.303*	
H77B	1.1088	1.1970	0.1933	0.303*	
C78	1.1246 (10)	0.855 (3)	0.1909 (5)	0.192 (8)	
H78A	1.1688	0.8744	0.1755	0.230*	
H78B	1.1455	0.7395	0.2096	0.230*	
C79	1.0366 (11)	0.782 (4)	0.1656 (7)	0.316 (18)	
H79A	1.0336	0.8529	0.1411	0.379*	
H79B	1.0412	0.6223	0.1616	0.379*	

### (8) (S)-(+)-2-{[(1-Cyclohexylethyl)imino]methyl}naphthalene Atomic displacement parameters (Å^2^)

**Table:**

	*U*^11^	*U*^22^	*U*^33^	*U*^12^	*U*^13^	*U*^23^
N1	0.117 (8)	0.117 (8)	0.094 (8)	−0.004 (7)	0.013 (6)	−0.004 (7)
C1	0.055 (6)	0.063 (6)	0.104 (9)	−0.011 (5)	0.006 (6)	0.011 (6)
C2	0.057 (6)	0.072 (7)	0.124 (10)	−0.008 (5)	0.014 (6)	−0.002 (7)
C3	0.045 (5)	0.061 (6)	0.093 (8)	−0.003 (4)	0.000 (5)	0.000 (6)
C4	0.074 (7)	0.067 (6)	0.110 (9)	−0.008 (5)	−0.004 (7)	0.002 (7)
C5	0.074 (7)	0.074 (8)	0.151 (12)	−0.004 (6)	−0.012 (8)	0.014 (8)
C6	0.100 (9)	0.089 (8)	0.107 (9)	−0.020 (7)	−0.002 (7)	0.013 (8)
C7	0.073 (7)	0.072 (7)	0.113 (10)	−0.012 (5)	0.010 (7)	−0.015 (7)
C8	0.049 (6)	0.068 (7)	0.115 (9)	−0.004 (5)	−0.002 (6)	0.014 (7)
C9	0.058 (6)	0.057 (6)	0.144 (11)	0.009 (5)	−0.002 (7)	−0.008 (7)
C10	0.075 (7)	0.060 (7)	0.123 (10)	−0.008 (5)	−0.020 (7)	0.005 (7)
C11	0.086 (8)	0.092 (8)	0.106 (10)	−0.011 (6)	0.007 (7)	−0.014 (8)
C12	0.134 (13)	0.145 (12)	0.111 (11)	−0.001 (10)	0.016 (10)	−0.017 (9)
C13	0.123 (13)	0.37 (3)	0.156 (14)	−0.026 (18)	0.053 (11)	0.000 (17)
C14	0.129 (13)	0.168 (16)	0.190 (17)	−0.055 (13)	−0.012 (13)	0.017 (14)
C15	0.18 (2)	0.184 (19)	0.32 (3)	−0.024 (16)	0.01 (2)	0.04 (2)
C16	0.18 (2)	0.32 (3)	0.23 (2)	0.00 (2)	−0.025 (19)	0.14 (2)
C17	0.112 (16)	0.25 (3)	0.43 (4)	0.035 (19)	0.01 (2)	0.08 (3)
C18	0.21 (3)	0.37 (4)	0.29 (3)	−0.16 (3)	−0.06 (2)	0.06 (3)
C19	0.22 (2)	0.40 (4)	0.169 (17)	−0.19 (2)	−0.062 (16)	0.13 (2)
N2	0.094 (6)	0.074 (5)	0.084 (6)	−0.007 (5)	0.006 (5)	−0.003 (5)
C21	0.047 (5)	0.071 (6)	0.078 (7)	0.001 (4)	0.012 (5)	−0.007 (6)
C22	0.048 (5)	0.052 (5)	0.091 (7)	−0.007 (4)	0.013 (5)	−0.005 (5)
C23	0.053 (5)	0.046 (5)	0.079 (6)	−0.002 (4)	0.013 (5)	0.005 (5)
C24	0.062 (6)	0.066 (6)	0.098 (8)	−0.003 (5)	0.009 (6)	0.003 (6)
C25	0.091 (8)	0.086 (7)	0.088 (8)	−0.011 (6)	0.011 (6)	0.024 (6)
C26	0.097 (8)	0.106 (9)	0.083 (8)	−0.026 (7)	−0.003 (6)	0.004 (7)
C27	0.072 (7)	0.075 (7)	0.099 (8)	−0.012 (5)	0.011 (6)	−0.012 (6)
C28	0.065 (6)	0.059 (5)	0.071 (6)	−0.003 (4)	0.010 (5)	0.005 (5)
C29	0.064 (6)	0.051 (5)	0.101 (8)	0.000 (4)	0.017 (6)	−0.007 (6)
C30	0.060 (6)	0.061 (6)	0.092 (7)	0.000 (5)	0.026 (5)	−0.003 (5)
C31	0.056 (6)	0.068 (6)	0.094 (8)	0.004 (5)	0.016 (5)	0.004 (6)
C32	0.085 (7)	0.085 (7)	0.092 (8)	−0.009 (6)	0.015 (6)	−0.002 (6)
C33	0.091 (8)	0.132 (9)	0.108 (9)	0.023 (8)	0.009 (7)	−0.031 (8)
C34	0.092 (8)	0.083 (7)	0.099 (8)	0.006 (6)	−0.003 (7)	−0.001 (7)
C35	0.168 (13)	0.100 (9)	0.108 (10)	−0.009 (9)	0.017 (10)	−0.006 (8)
C36	0.192 (17)	0.126 (12)	0.156 (14)	−0.007 (12)	0.072 (13)	−0.043 (11)
C37	0.150 (13)	0.149 (13)	0.127 (12)	0.026 (11)	0.043 (10)	−0.017 (11)
C38	0.096 (9)	0.138 (11)	0.151 (12)	0.003 (8)	0.048 (9)	−0.016 (10)
C39	0.097 (8)	0.086 (7)	0.129 (10)	−0.008 (6)	0.032 (8)	−0.024 (7)
N3	0.098 (7)	0.114 (8)	0.083 (6)	0.007 (6)	−0.006 (6)	−0.021 (6)
C41	0.048 (5)	0.061 (6)	0.090 (7)	0.011 (4)	0.016 (5)	−0.005 (5)
C42	0.049 (5)	0.065 (6)	0.084 (7)	0.006 (4)	0.016 (5)	0.000 (5)
C43	0.043 (5)	0.066 (6)	0.078 (7)	0.004 (4)	0.017 (5)	−0.001 (5)
C44	0.071 (6)	0.062 (6)	0.087 (7)	−0.004 (5)	0.017 (6)	−0.010 (6)
C45	0.082 (7)	0.084 (7)	0.090 (8)	0.001 (6)	0.029 (6)	0.002 (6)
C46	0.073 (7)	0.099 (8)	0.091 (8)	−0.004 (6)	0.022 (6)	−0.010 (7)
C47	0.061 (6)	0.056 (5)	0.126 (9)	−0.007 (5)	0.024 (6)	−0.016 (6)
C48	0.050 (5)	0.062 (6)	0.084 (7)	0.010 (5)	0.018 (5)	−0.006 (6)
C49	0.057 (6)	0.050 (5)	0.113 (8)	0.008 (4)	0.030 (6)	0.008 (6)
C50	0.069 (7)	0.065 (6)	0.089 (7)	0.005 (5)	0.017 (6)	0.003 (6)
C51	0.057 (6)	0.097 (7)	0.077 (7)	−0.003 (5)	0.013 (5)	−0.004 (7)
C52	0.098 (9)	0.129 (10)	0.079 (8)	0.006 (8)	0.001 (7)	−0.009 (7)
C53	0.101 (10)	0.28 (2)	0.146 (12)	0.083 (13)	−0.016 (9)	−0.056 (14)
C54	0.101 (8)	0.103 (8)	0.066 (7)	−0.005 (7)	0.013 (6)	0.002 (6)
C55	0.243 (18)	0.111 (10)	0.082 (9)	0.033 (11)	0.038 (10)	0.017 (8)
C56	0.30 (3)	0.111 (12)	0.117 (13)	−0.032 (14)	0.110 (15)	0.004 (10)
C57	0.144 (15)	0.161 (15)	0.210 (19)	−0.047 (13)	0.080 (14)	−0.046 (14)
C58	0.096 (9)	0.140 (11)	0.154 (12)	0.014 (9)	0.045 (9)	−0.015 (10)
C59	0.087 (8)	0.122 (9)	0.099 (8)	0.001 (7)	0.015 (7)	0.000 (7)
N4	0.097 (7)	0.112 (7)	0.096 (7)	0.008 (6)	0.005 (6)	0.015 (7)
C61	0.058 (6)	0.079 (7)	0.099 (8)	0.003 (5)	0.014 (6)	0.025 (7)
C62	0.046 (5)	0.064 (6)	0.107 (8)	0.000 (4)	0.012 (5)	0.020 (6)
C63	0.058 (6)	0.051 (6)	0.100 (8)	−0.003 (4)	0.018 (6)	0.003 (6)
C64	0.091 (8)	0.080 (7)	0.100 (9)	0.009 (6)	0.010 (7)	0.006 (7)
C65	0.092 (9)	0.105 (9)	0.107 (10)	0.004 (8)	0.001 (7)	0.010 (8)
C66	0.100 (9)	0.103 (10)	0.118 (11)	−0.008 (8)	0.019 (8)	−0.024 (8)
C67	0.068 (7)	0.080 (7)	0.133 (10)	0.000 (6)	0.024 (7)	0.004 (8)
C68	0.042 (5)	0.068 (6)	0.126 (10)	−0.006 (5)	0.016 (6)	−0.009 (7)
C69	0.070 (7)	0.064 (7)	0.124 (10)	0.001 (5)	0.001 (7)	0.029 (7)
C70	0.073 (7)	0.069 (7)	0.103 (8)	−0.002 (6)	0.003 (6)	0.012 (6)
C71	0.083 (7)	0.092 (8)	0.100 (9)	0.008 (6)	0.017 (7)	0.013 (7)
C72	0.096 (9)	0.134 (11)	0.110 (10)	0.028 (8)	0.022 (8)	0.017 (9)
C73	0.183 (16)	0.186 (15)	0.109 (11)	0.032 (13)	0.003 (11)	0.025 (11)
C74	0.109 (11)	0.175 (14)	0.186 (15)	0.042 (11)	−0.012 (11)	−0.088 (12)
C75	0.158 (15)	0.180 (15)	0.182 (15)	0.030 (13)	−0.004 (13)	−0.082 (13)
C76	0.21 (3)	0.38 (4)	0.35 (4)	−0.02 (3)	0.10 (3)	−0.22 (3)
C77	0.28 (3)	0.20 (2)	0.27 (3)	−0.06 (2)	0.05 (2)	−0.162 (19)
C78	0.137 (14)	0.215 (19)	0.194 (16)	0.027 (14)	−0.032 (12)	−0.079 (15)
C79	0.112 (14)	0.37 (3)	0.43 (4)	0.029 (19)	−0.02 (2)	−0.26 (3)

### (8) (S)-(+)-2-{[(1-Cyclohexylethyl)imino]methyl}naphthalene Geometric parameters (Å, º)

**Table:**

N1—C11	1.248 (13)	N3—C51	1.255 (11)
N1—C12	1.472 (15)	N3—C52	1.478 (13)
C1—C2	1.355 (12)	C41—C42	1.390 (12)
C1—C10	1.396 (14)	C41—C50	1.417 (13)
C1—C11	1.493 (14)	C41—C51	1.462 (12)
C2—C3	1.439 (13)	C42—C43	1.406 (11)
C2—H2A	0.9300	C42—H42A	0.9300
C3—C4	1.385 (12)	C43—C44	1.418 (12)
C3—C8	1.460 (13)	C43—C48	1.436 (12)
C4—C5	1.387 (15)	C44—C45	1.357 (12)
C4—H4A	0.9300	C44—H44A	0.9300
C5—C6	1.457 (16)	C45—C46	1.436 (14)
C5—H5A	0.9300	C45—H45A	0.9300
C6—C7	1.360 (14)	C46—C47	1.378 (13)
C6—H6A	0.9300	C46—H46A	0.9300
C7—C8	1.376 (14)	C47—C48	1.406 (12)
C7—H7A	0.9300	C47—H47A	0.9300
C8—C9	1.411 (13)	C48—C49	1.403 (12)
C9—C10	1.355 (14)	C49—C50	1.368 (12)
C9—H9A	0.9300	C49—H49A	0.9300
C10—H10A	0.9300	C50—H50A	0.9300
C11—H11A	0.9300	C51—H51A	0.9300
C12—C13	1.522 (19)	C52—C53	1.507 (15)
C12—C14	1.549 (18)	C52—C54	1.567 (15)
C12—H12A	0.9800	C52—H52A	0.9800
C13—H13A	0.9600	C53—H53A	0.9600
C13—H13B	0.9600	C53—H53B	0.9600
C13—H13C	0.9600	C53—H53C	0.9600
C14—C15	1.411 (17)	C54—C55	1.473 (16)
C14—C19	1.447 (17)	C54—C59	1.493 (14)
C14—H14A	0.9800	C54—H54A	0.9800
C15—C16	1.573 (17)	C55—C56	1.55 (2)
C15—H15A	0.9700	C55—H55A	0.9700
C15—H15B	0.9700	C55—H55B	0.9700
C16—C17	1.50 (2)	C56—C57	1.53 (2)
C16—H16A	0.9700	C56—H56A	0.9700
C16—H16B	0.9700	C56—H56B	0.9700
C17—C18	1.46 (2)	C57—C58	1.463 (19)
C17—H17A	0.9700	C57—H57A	0.9700
C17—H17B	0.9700	C57—H57B	0.9700
C18—C19	1.591 (18)	C58—C59	1.531 (15)
C18—H18A	0.9700	C58—H58A	0.9700
C18—H18B	0.9700	C58—H58B	0.9700
C19—H19A	0.9700	C59—H59A	0.9700
C19—H19B	0.9700	C59—H59B	0.9700
N2—C31	1.273 (11)	N4—C71	1.272 (11)
N2—C32	1.477 (11)	N4—C72	1.493 (14)
C21—C22	1.353 (11)	C61—C62	1.369 (12)
C21—C30	1.423 (12)	C61—C70	1.422 (13)
C21—C31	1.477 (12)	C61—C71	1.481 (14)
C22—C23	1.388 (11)	C62—C63	1.413 (12)
C22—H22A	0.9300	C62—H62A	0.9300
C23—C24	1.416 (11)	C63—C64	1.372 (13)
C23—C28	1.443 (11)	C63—C68	1.406 (13)
C24—C25	1.368 (12)	C64—C65	1.376 (14)
C24—H24A	0.9300	C64—H64A	0.9300
C25—C26	1.386 (13)	C65—C66	1.419 (16)
C25—H25A	0.9300	C65—H65A	0.9300
C26—C27	1.363 (13)	C66—C67	1.338 (14)
C26—H26A	0.9300	C66—H66A	0.9300
C27—C28	1.400 (12)	C67—C68	1.410 (14)
C27—H27A	0.9300	C67—H67A	0.9300
C28—C29	1.422 (12)	C68—C69	1.428 (14)
C29—C30	1.358 (11)	C69—C70	1.364 (13)
C29—H29A	0.9300	C69—H69A	0.9300
C30—H30A	0.9300	C70—H70A	0.9300
C31—H31A	0.9300	C71—H71A	0.9300
C32—C34	1.532 (14)	C72—C73	1.499 (18)
C32—C33	1.538 (13)	C72—C74	1.520 (17)
C32—H32A	0.9800	C72—H72A	0.9800
C33—H33A	0.9600	C73—H73A	0.9600
C33—H33B	0.9600	C73—H73B	0.9600
C33—H33C	0.9600	C73—H73C	0.9600
C34—C39	1.479 (13)	C74—C79	1.315 (17)
C34—C35	1.515 (12)	C74—C75	1.488 (15)
C34—H34A	0.9800	C74—H74A	0.9800
C35—C36	1.535 (15)	C75—C76	1.531 (19)
C35—H35A	0.9700	C75—H75A	0.9700
C35—H35B	0.9700	C75—H75B	0.9700
C36—C37	1.476 (16)	C76—C77	1.41 (2)
C36—H36A	0.9700	C76—H76A	0.9700
C36—H36B	0.9700	C76—H76B	0.9700
C37—C38	1.508 (14)	C77—C78	1.521 (17)
C37—H37A	0.9700	C77—H77A	0.9700
C37—H37B	0.9700	C77—H77B	0.9700
C38—C39	1.554 (13)	C78—C79	1.531 (16)
C38—H38A	0.9700	C78—H78A	0.9700
C38—H38B	0.9700	C78—H78B	0.9700
C39—H39A	0.9700	C79—H79A	0.9700
C39—H39B	0.9700	C79—H79B	0.9700
			
C11—N1—C12	117.3 (12)	C51—N3—C52	119.5 (10)
C2—C1—C10	120.8 (11)	C42—C41—C50	119.1 (9)
C2—C1—C11	118.6 (10)	C42—C41—C51	119.6 (9)
C10—C1—C11	120.5 (10)	C50—C41—C51	121.4 (10)
C1—C2—C3	120.6 (10)	C41—C42—C43	121.0 (9)
C1—C2—H2A	119.7	C41—C42—H42A	119.5
C3—C2—H2A	119.7	C43—C42—H42A	119.5
C4—C3—C2	120.9 (10)	C42—C43—C44	122.6 (9)
C4—C3—C8	120.4 (10)	C42—C43—C48	119.4 (9)
C2—C3—C8	118.6 (9)	C44—C43—C48	117.9 (9)
C3—C4—C5	118.9 (11)	C45—C44—C43	122.4 (9)
C3—C4—H4A	120.6	C45—C44—H44A	118.8
C5—C4—H4A	120.6	C43—C44—H44A	118.8
C4—C5—C6	122.8 (11)	C44—C45—C46	119.4 (10)
C4—C5—H5A	118.6	C44—C45—H45A	120.3
C6—C5—H5A	118.6	C46—C45—H45A	120.3
C7—C6—C5	114.5 (12)	C47—C46—C45	119.7 (10)
C7—C6—H6A	122.7	C47—C46—H46A	120.1
C5—C6—H6A	122.7	C45—C46—H46A	120.1
C6—C7—C8	126.5 (11)	C46—C47—C48	121.3 (10)
C6—C7—H7A	116.8	C46—C47—H47A	119.4
C8—C7—H7A	116.8	C48—C47—H47A	119.4
C7—C8—C9	126.8 (11)	C49—C48—C47	122.6 (9)
C7—C8—C3	116.6 (10)	C49—C48—C43	118.2 (9)
C9—C8—C3	116.6 (11)	C47—C48—C43	119.2 (9)
C10—C9—C8	122.7 (10)	C50—C49—C48	121.6 (9)
C10—C9—H9A	118.6	C50—C49—H49A	119.2
C8—C9—H9A	118.6	C48—C49—H49A	119.2
C9—C10—C1	120.6 (11)	C49—C50—C41	120.6 (9)
C9—C10—H10A	119.7	C49—C50—H50A	119.7
C1—C10—H10A	119.7	C41—C50—H50A	119.7
N1—C11—C1	122.8 (11)	N3—C51—C41	121.3 (10)
N1—C11—H11A	118.6	N3—C51—H51A	119.4
C1—C11—H11A	118.6	C41—C51—H51A	119.4
N1—C12—C13	108.8 (13)	N3—C52—C53	109.7 (11)
N1—C12—C14	112.0 (14)	N3—C52—C54	108.9 (10)
C13—C12—C14	112.3 (14)	C53—C52—C54	111.6 (10)
N1—C12—H12A	107.9	N3—C52—H52A	108.9
C13—C12—H12A	107.9	C53—C52—H52A	108.9
C14—C12—H12A	107.9	C54—C52—H52A	108.9
C12—C13—H13A	109.5	C52—C53—H53A	109.5
C12—C13—H13B	109.5	C52—C53—H53B	109.5
H13A—C13—H13B	109.5	H53A—C53—H53B	109.5
C12—C13—H13C	109.5	C52—C53—H53C	109.5
H13A—C13—H13C	109.5	H53A—C53—H53C	109.5
H13B—C13—H13C	109.5	H53B—C53—H53C	109.5
C15—C14—C19	109.7 (18)	C55—C54—C59	111.4 (11)
C15—C14—C12	116.4 (15)	C55—C54—C52	112.2 (11)
C19—C14—C12	110.7 (15)	C59—C54—C52	111.5 (9)
C15—C14—H14A	106.5	C55—C54—H54A	107.2
C19—C14—H14A	106.5	C59—C54—H54A	107.2
C12—C14—H14A	106.5	C52—C54—H54A	107.2
C14—C15—C16	109.8 (16)	C54—C55—C56	109.5 (11)
C14—C15—H15A	109.7	C54—C55—H55A	109.8
C16—C15—H15A	109.7	C56—C55—H55A	109.8
C14—C15—H15B	109.7	C54—C55—H55B	109.8
C16—C15—H15B	109.7	C56—C55—H55B	109.8
H15A—C15—H15B	108.2	H55A—C55—H55B	108.2
C17—C16—C15	107.8 (16)	C57—C56—C55	109.6 (12)
C17—C16—H16A	110.1	C57—C56—H56A	109.7
C15—C16—H16A	110.1	C55—C56—H56A	109.7
C17—C16—H16B	110.1	C57—C56—H56B	109.7
C15—C16—H16B	110.1	C55—C56—H56B	109.7
H16A—C16—H16B	108.5	H56A—C56—H56B	108.2
C18—C17—C16	104 (2)	C58—C57—C56	108.4 (13)
C18—C17—H17A	110.9	C58—C57—H57A	110.0
C16—C17—H17A	110.9	C56—C57—H57A	110.0
C18—C17—H17B	110.9	C58—C57—H57B	110.0
C16—C17—H17B	110.9	C56—C57—H57B	110.0
H17A—C17—H17B	108.9	H57A—C57—H57B	108.4
C17—C18—C19	107.0 (18)	C57—C58—C59	108.9 (12)
C17—C18—H18A	110.3	C57—C58—H58A	109.9
C19—C18—H18A	110.3	C59—C58—H58A	109.9
C17—C18—H18B	110.3	C57—C58—H58B	109.9
C19—C18—H18B	110.3	C59—C58—H58B	109.9
H18A—C18—H18B	108.6	H58A—C58—H58B	108.3
C14—C19—C18	109.7 (14)	C54—C59—C58	112.5 (10)
C14—C19—H19A	109.7	C54—C59—H59A	109.1
C18—C19—H19A	109.7	C58—C59—H59A	109.1
C14—C19—H19B	109.7	C54—C59—H59B	109.1
C18—C19—H19B	109.7	C58—C59—H59B	109.1
H19A—C19—H19B	108.2	H59A—C59—H59B	107.8
C31—N2—C32	118.8 (8)	C71—N4—C72	116.0 (10)
C22—C21—C30	120.5 (8)	C62—C61—C70	118.4 (11)
C22—C21—C31	120.2 (8)	C62—C61—C71	121.8 (10)
C30—C21—C31	119.2 (9)	C70—C61—C71	119.8 (10)
C21—C22—C23	121.8 (8)	C61—C62—C63	124.3 (9)
C21—C22—H22A	119.1	C61—C62—H62A	117.8
C23—C22—H22A	119.1	C63—C62—H62A	117.8
C22—C23—C24	123.4 (8)	C64—C63—C68	122.3 (11)
C22—C23—C28	118.8 (8)	C64—C63—C62	121.2 (9)
C24—C23—C28	117.8 (8)	C68—C63—C62	116.4 (10)
C25—C24—C23	120.8 (9)	C65—C64—C63	118.5 (11)
C25—C24—H24A	119.6	C65—C64—H64A	120.8
C23—C24—H24A	119.6	C63—C64—H64A	120.8
C24—C25—C26	120.7 (10)	C64—C65—C66	120.7 (12)
C24—C25—H25A	119.6	C64—C65—H65A	119.6
C26—C25—H25A	119.6	C66—C65—H65A	119.6
C27—C26—C25	120.7 (10)	C67—C66—C65	119.7 (13)
C27—C26—H26A	119.7	C67—C66—H66A	120.2
C25—C26—H26A	119.7	C65—C66—H66A	120.2
C26—C27—C28	121.1 (9)	C66—C67—C68	121.6 (11)
C26—C27—H27A	119.5	C66—C67—H67A	119.2
C28—C27—H27A	119.5	C68—C67—H67A	119.2
C27—C28—C29	123.0 (9)	C63—C68—C67	117.1 (11)
C27—C28—C23	118.9 (8)	C63—C68—C69	119.6 (11)
C29—C28—C23	118.1 (8)	C67—C68—C69	123.2 (10)
C30—C29—C28	121.2 (9)	C70—C69—C68	121.9 (10)
C30—C29—H29A	119.4	C70—C69—H69A	119.1
C28—C29—H29A	119.4	C68—C69—H69A	119.1
C29—C30—C21	119.6 (9)	C69—C70—C61	119.3 (10)
C29—C30—H30A	120.2	C69—C70—H70A	120.3
C21—C30—H30A	120.2	C61—C70—H70A	120.3
N2—C31—C21	124.4 (9)	N4—C71—C61	122.1 (11)
N2—C31—H31A	117.8	N4—C71—H71A	118.9
C21—C31—H31A	117.8	C61—C71—H71A	118.9
N2—C32—C34	108.9 (9)	N4—C72—C73	106.5 (13)
N2—C32—C33	108.0 (9)	N4—C72—C74	109.8 (10)
C34—C32—C33	113.2 (9)	C73—C72—C74	111.7 (13)
N2—C32—H32A	108.9	N4—C72—H72A	109.6
C34—C32—H32A	108.9	C73—C72—H72A	109.6
C33—C32—H32A	108.9	C74—C72—H72A	109.6
C32—C33—H33A	109.5	C72—C73—H73A	109.5
C32—C33—H33B	109.5	C72—C73—H73B	109.5
H33A—C33—H33B	109.5	H73A—C73—H73B	109.5
C32—C33—H33C	109.5	C72—C73—H73C	109.5
H33A—C33—H33C	109.5	H73A—C73—H73C	109.5
H33B—C33—H33C	109.5	H73B—C73—H73C	109.5
C32—C34—C39	115.5 (9)	C79—C74—C72	123.9 (14)
C32—C34—C35	112.2 (10)	C79—C74—C75	114.4 (16)
C39—C34—C35	110.5 (9)	C72—C74—C75	111.7 (13)
C32—C34—H34A	106.0	C79—C74—H74A	100.6
C39—C34—H34A	106.0	C72—C74—H74A	100.6
C35—C34—H34A	106.0	C75—C74—H74A	100.6
C36—C35—C34	110.9 (11)	C76—C75—C74	111.0 (14)
C36—C35—H35A	109.5	C76—C75—H75A	109.4
C34—C35—H35A	109.5	C74—C75—H75A	109.4
C36—C35—H35B	109.5	C76—C75—H75B	109.4
C34—C35—H35B	109.5	C74—C75—H75B	109.4
H35A—C35—H35B	108.0	H75A—C75—H75B	108.0
C37—C36—C35	111.6 (11)	C77—C76—C75	112 (2)
C37—C36—H36A	109.3	C77—C76—H76A	109.3
C35—C36—H36A	109.3	C75—C76—H76A	109.3
C37—C36—H36B	109.3	C77—C76—H76B	109.3
C35—C36—H36B	109.3	C75—C76—H76B	109.3
H36A—C36—H36B	108.0	H76A—C76—H76B	108.0
C36—C37—C38	111.9 (12)	C76—C77—C78	110.1 (18)
C36—C37—H37A	109.2	C76—C77—H77A	109.6
C38—C37—H37A	109.2	C78—C77—H77A	109.6
C36—C37—H37B	109.2	C76—C77—H77B	109.6
C38—C37—H37B	109.2	C78—C77—H77B	109.6
H37A—C37—H37B	107.9	H77A—C77—H77B	108.2
C37—C38—C39	110.4 (10)	C79—C78—C77	110.7 (14)
C37—C38—H38A	109.6	C79—C78—H78A	109.5
C39—C38—H38A	109.6	C77—C78—H78A	109.5
C37—C38—H38B	109.6	C79—C78—H78B	109.5
C39—C38—H38B	109.6	C77—C78—H78B	109.5
H38A—C38—H38B	108.1	H78A—C78—H78B	108.1
C34—C39—C38	112.1 (9)	C74—C79—C78	123.1 (16)
C34—C39—H39A	109.2	C74—C79—H79A	106.6
C38—C39—H39A	109.2	C78—C79—H79A	106.6
C34—C39—H39B	109.2	C74—C79—H79B	106.6
C38—C39—H39B	109.2	C78—C79—H79B	106.6
H39A—C39—H39B	107.9	H79A—C79—H79B	106.5
			
C10—C1—C2—C3	1.7 (14)	C50—C41—C42—C43	−2.9 (12)
C11—C1—C2—C3	179.4 (8)	C51—C41—C42—C43	177.4 (8)
C1—C2—C3—C4	−178.8 (9)	C41—C42—C43—C44	−177.5 (8)
C1—C2—C3—C8	−2.7 (13)	C41—C42—C43—C48	0.8 (12)
C2—C3—C4—C5	176.8 (9)	C42—C43—C44—C45	178.6 (9)
C8—C3—C4—C5	0.7 (14)	C48—C43—C44—C45	0.2 (13)
C3—C4—C5—C6	−5.0 (15)	C43—C44—C45—C46	0.6 (15)
C4—C5—C6—C7	7.5 (15)	C44—C45—C46—C47	−1.0 (14)
C5—C6—C7—C8	−6.2 (16)	C45—C46—C47—C48	0.6 (14)
C6—C7—C8—C9	−176.3 (10)	C46—C47—C48—C49	178.5 (9)
C6—C7—C8—C3	2.4 (15)	C46—C47—C48—C43	0.2 (13)
C4—C3—C8—C7	0.7 (13)	C42—C43—C48—C49	2.6 (11)
C2—C3—C8—C7	−175.5 (8)	C44—C43—C48—C49	−179.0 (8)
C4—C3—C8—C9	179.5 (8)	C42—C43—C48—C47	−179.0 (8)
C2—C3—C8—C9	3.3 (12)	C44—C43—C48—C47	−0.6 (11)
C7—C8—C9—C10	175.4 (10)	C47—C48—C49—C50	177.7 (8)
C3—C8—C9—C10	−3.2 (14)	C43—C48—C49—C50	−4.1 (12)
C8—C9—C10—C1	2.3 (16)	C48—C49—C50—C41	2.0 (13)
C2—C1—C10—C9	−1.5 (15)	C42—C41—C50—C49	1.5 (12)
C11—C1—C10—C9	−179.1 (9)	C51—C41—C50—C49	−178.8 (8)
C12—N1—C11—C1	179.1 (10)	C52—N3—C51—C41	−179.8 (9)
C2—C1—C11—N1	173.7 (11)	C42—C41—C51—N3	−177.3 (9)
C10—C1—C11—N1	−8.6 (16)	C50—C41—C51—N3	3.0 (14)
C11—N1—C12—C13	103.3 (16)	C51—N3—C52—C53	116.4 (13)
C11—N1—C12—C14	−131.9 (14)	C51—N3—C52—C54	−121.2 (11)
N1—C12—C14—C15	−66 (2)	N3—C52—C54—C55	−55.6 (13)
C13—C12—C14—C15	56 (3)	C53—C52—C54—C55	65.7 (14)
N1—C12—C14—C19	60 (2)	N3—C52—C54—C59	70.1 (12)
C13—C12—C14—C19	−177.6 (18)	C53—C52—C54—C59	−168.6 (11)
C19—C14—C15—C16	59 (2)	C59—C54—C55—C56	54.5 (15)
C12—C14—C15—C16	−174.1 (16)	C52—C54—C55—C56	−179.7 (11)
C14—C15—C16—C17	−64 (3)	C54—C55—C56—C57	−59.0 (18)
C15—C16—C17—C18	66 (3)	C55—C56—C57—C58	62.9 (17)
C16—C17—C18—C19	−66 (3)	C56—C57—C58—C59	−61.0 (17)
C15—C14—C19—C18	−59 (3)	C55—C54—C59—C58	−54.8 (13)
C12—C14—C19—C18	170.9 (18)	C52—C54—C59—C58	179.1 (10)
C17—C18—C19—C14	64 (3)	C57—C58—C59—C54	57.9 (15)
C30—C21—C22—C23	0.3 (13)	C70—C61—C62—C63	−2.4 (14)
C31—C21—C22—C23	177.1 (8)	C71—C61—C62—C63	179.0 (9)
C21—C22—C23—C24	−177.3 (8)	C61—C62—C63—C64	−177.5 (9)
C21—C22—C23—C28	−0.4 (12)	C61—C62—C63—C68	0.7 (13)
C22—C23—C24—C25	178.4 (9)	C68—C63—C64—C65	0.9 (15)
C28—C23—C24—C25	1.6 (14)	C62—C63—C64—C65	179.0 (10)
C23—C24—C25—C26	−1.2 (16)	C63—C64—C65—C66	−2.0 (17)
C24—C25—C26—C27	−0.1 (17)	C64—C65—C66—C67	3.4 (18)
C25—C26—C27—C28	1.0 (17)	C65—C66—C67—C68	−3.6 (17)
C26—C27—C28—C29	−179.5 (10)	C64—C63—C68—C67	−1.1 (13)
C26—C27—C28—C23	−0.6 (15)	C62—C63—C68—C67	−179.2 (8)
C22—C23—C28—C27	−177.7 (9)	C64—C63—C68—C69	180.0 (9)
C24—C23—C28—C27	−0.7 (13)	C62—C63—C68—C69	1.9 (12)
C22—C23—C28—C29	1.3 (12)	C66—C67—C68—C63	2.4 (15)
C24—C23—C28—C29	178.3 (8)	C66—C67—C68—C69	−178.7 (10)
C27—C28—C29—C30	176.8 (9)	C63—C68—C69—C70	−2.7 (14)
C23—C28—C29—C30	−2.1 (13)	C67—C68—C69—C70	178.4 (9)
C28—C29—C30—C21	2.0 (13)	C68—C69—C70—C61	1.0 (15)
C22—C21—C30—C29	−1.1 (13)	C62—C61—C70—C69	1.5 (14)
C31—C21—C30—C29	−177.9 (8)	C71—C61—C70—C69	−179.8 (9)
C32—N2—C31—C21	178.6 (8)	C72—N4—C71—C61	176.9 (10)
C22—C21—C31—N2	178.8 (9)	C62—C61—C71—N4	−178.7 (10)
C30—C21—C31—N2	−4.4 (13)	C70—C61—C71—N4	2.7 (16)
C31—N2—C32—C34	−116.4 (10)	C71—N4—C72—C73	113.8 (12)
C31—N2—C32—C33	120.3 (10)	C71—N4—C72—C74	−125.0 (14)
N2—C32—C34—C39	−62.6 (12)	N4—C72—C74—C79	22 (3)
C33—C32—C34—C39	57.5 (12)	C73—C72—C74—C79	140 (2)
N2—C32—C34—C35	169.6 (9)	N4—C72—C74—C75	165.1 (14)
C33—C32—C34—C35	−70.3 (12)	C73—C72—C74—C75	−77.0 (19)
C32—C34—C35—C36	−173.4 (11)	C79—C74—C75—C76	−44 (3)
C39—C34—C35—C36	56.1 (14)	C72—C74—C75—C76	169.3 (18)
C34—C35—C36—C37	−56.3 (16)	C74—C75—C76—C77	59 (3)
C35—C36—C37—C38	55.6 (17)	C75—C76—C77—C78	−60 (3)
C36—C37—C38—C39	−54.0 (16)	C76—C77—C78—C79	46 (3)
C32—C34—C39—C38	175.5 (9)	C72—C74—C79—C78	177.2 (19)
C35—C34—C39—C38	−55.8 (13)	C75—C74—C79—C78	35 (4)
C37—C38—C39—C34	54.6 (15)	C77—C78—C79—C74	−35 (4)

### (9) (S)-(–)-2-{[(1,2,3,4-Tetrahydronaphthalen-1-yl)imino]methyl}naphthalene Crystal data

**Table:**

C_21_H_19_N	*D*_x_ = 1.159 Mg m^−^^3^
*M**_r_* = 285.37	Melting point: 359 K
Monoclinic, *P*2_1_	Mo *K*α radiation, λ = 0.7107 Å
*a* = 7.7571 (13) Å	Cell parameters from 2340 reflections
*b* = 5.9246 (10) Å	θ = 3.4–24.8°
*c* = 17.820 (4) Å	µ = 0.07 mm^−^^1^
β = 92.682 (16)°	*T* = 298 K
*V* = 818.1 (2) Å^3^	Plate, colorless
*Z* = 2	0.88 × 0.43 × 0.08 mm
*F*(000) = 304	

### (9) (S)-(–)-2-{[(1,2,3,4-Tetrahydronaphthalen-1-yl)imino]methyl}naphthalene Data collection

**Table:**

Agilent Xcalibur Atlas Gemini diffractometer	2884 independent reflections
Radiation source: Enhance (Mo) X-ray Source	1822 reflections with *I* > 2σ(*I*)
Graphite monochromator	*R*_int_ = 0.081
ω scans	θ_max_ = 25.0°, θ_min_ = 3.4°
Absorption correction: analytical (CrysAlis PRO; Agilent, 2013)	*h* = −9→9
*T*_min_ = 0.857, *T*_max_ = 0.983	*k* = −7→7
15266 measured reflections	*l* = −21→21

### (9) (S)-(–)-2-{[(1,2,3,4-Tetrahydronaphthalen-1-yl)imino]methyl}naphthalene Refinement

**Table:**

Refinement on *F*^2^	Primary atom site location: structure-invariant direct methods
Least-squares matrix: full	Secondary atom site location: difference Fourier map
*R*[*F*^2^ > 2σ(*F*^2^)] = 0.069	Hydrogen site location: inferred from neighbouring sites
*wR*(*F*^2^) = 0.175	H-atom parameters constrained
*S* = 1.44	*w* = 1/[σ^2^(*F*_o_^2^) + (0.050*P*)^2^] where *P* = (*F*_o_^2^ + 2*F*_c_^2^)/3
2884 reflections	(Δ/σ)_max_ < 0.001
199 parameters	Δρ_max_ = 0.21 e Å^−^^3^
1 restraint	Δρ_min_ = −0.19 e Å^−^^3^
0 constraints	

### (9) (S)-(–)-2-{[(1,2,3,4-Tetrahydronaphthalen-1-yl)imino]methyl}naphthalene Fractional atomic coordinates and isotropic or equivalent isotropic displacement parameters (Å^2^)

**Table:**

	*x*	*y*	*z*	*U*_iso_*/*U*_eq_	
N1	0.0570 (5)	0.8729 (8)	0.2802 (2)	0.0759 (12)	
C1	0.1450 (5)	0.8980 (8)	0.4110 (2)	0.0520 (11)	
C2	0.1520 (5)	0.7961 (7)	0.4795 (2)	0.0513 (11)	
H2A	0.0985	0.6570	0.4848	0.062*	
C3	0.2383 (4)	0.8954 (7)	0.5428 (2)	0.0505 (11)	
C4	0.2520 (5)	0.7902 (8)	0.6136 (2)	0.0623 (12)	
H4A	0.1981	0.6519	0.6203	0.075*	
C5	0.3425 (6)	0.8863 (10)	0.6725 (3)	0.0746 (14)	
H5A	0.3530	0.8119	0.7184	0.090*	
C6	0.4205 (6)	1.0996 (10)	0.6636 (3)	0.0736 (14)	
H6A	0.4812	1.1660	0.7041	0.088*	
C7	0.4077 (5)	1.2086 (9)	0.5965 (3)	0.0659 (13)	
H7A	0.4591	1.3493	0.5916	0.079*	
C8	0.3169 (5)	1.1104 (7)	0.5340 (3)	0.0512 (11)	
C9	0.3038 (5)	1.2154 (8)	0.4628 (3)	0.0603 (12)	
H9A	0.3530	1.3570	0.4567	0.072*	
C10	0.2213 (6)	1.1146 (7)	0.4033 (3)	0.0576 (12)	
H10A	0.2143	1.1874	0.3571	0.069*	
C11	0.0660 (5)	0.7838 (8)	0.3448 (3)	0.0629 (13)	
H11A	0.0206	0.6399	0.3506	0.075*	
C12	−0.0153 (7)	0.7397 (11)	0.2176 (3)	0.0856 (16)	
H12A	−0.0539	0.5940	0.2368	0.103*	
C13	0.1227 (7)	0.6994 (13)	0.1637 (3)	0.0904 (17)	
C14	0.2407 (10)	0.5324 (18)	0.1776 (4)	0.144 (3)	
H14A	0.2306	0.4371	0.2186	0.173*	
C15	0.3808 (14)	0.504 (2)	0.1285 (6)	0.181 (6)	
H15A	0.4645	0.3940	0.1373	0.217*	
C16	0.3856 (10)	0.641 (3)	0.0708 (6)	0.172 (6)	
H16A	0.4749	0.6217	0.0383	0.206*	
C17	0.2684 (12)	0.813 (2)	0.0543 (4)	0.159 (4)	
H17A	0.2807	0.9044	0.0125	0.191*	
C18	0.1342 (8)	0.8459 (16)	0.1003 (4)	0.116 (2)	
C19	−0.0029 (12)	1.0224 (17)	0.0818 (5)	0.147 (3)	
H19A	0.0535	1.1620	0.0685	0.176*	
H19B	−0.0722	0.9726	0.0382	0.176*	
C20	−0.1181 (12)	1.0682 (18)	0.1435 (5)	0.142 (3)	
H20A	−0.0595	1.1677	0.1797	0.170*	
H20B	−0.2203	1.1459	0.1235	0.170*	
C21	−0.1706 (8)	0.8645 (17)	0.1819 (4)	0.125 (3)	
H21A	−0.2314	0.7657	0.1462	0.150*	
H21B	−0.2493	0.9042	0.2205	0.150*	

### (9) (S)-(–)-2-{[(1,2,3,4-Tetrahydronaphthalen-1-yl)imino]methyl}naphthalene Atomic displacement parameters (Å^2^)

**Table:**

	*U*^11^	*U*^22^	*U*^33^	*U*^12^	*U*^13^	*U*^23^
N1	0.075 (3)	0.081 (3)	0.073 (3)	−0.011 (3)	0.006 (2)	−0.009 (2)
C1	0.036 (2)	0.048 (3)	0.073 (3)	0.003 (2)	0.0109 (19)	0.000 (2)
C2	0.035 (2)	0.037 (3)	0.083 (3)	−0.0009 (19)	0.011 (2)	0.000 (2)
C3	0.0297 (19)	0.044 (3)	0.078 (3)	0.006 (2)	0.0121 (19)	0.000 (2)
C4	0.053 (2)	0.055 (3)	0.080 (3)	0.002 (2)	0.012 (2)	0.007 (2)
C5	0.061 (3)	0.086 (4)	0.077 (3)	0.011 (3)	0.006 (2)	0.005 (3)
C6	0.049 (3)	0.088 (4)	0.084 (3)	0.003 (3)	0.000 (2)	−0.018 (3)
C7	0.046 (2)	0.056 (3)	0.096 (4)	0.000 (3)	0.008 (2)	−0.016 (3)
C8	0.034 (2)	0.037 (3)	0.083 (3)	0.0043 (19)	0.012 (2)	−0.007 (2)
C9	0.044 (2)	0.041 (3)	0.097 (4)	0.000 (2)	0.017 (2)	−0.007 (3)
C10	0.049 (2)	0.047 (3)	0.077 (3)	0.004 (2)	0.013 (2)	0.006 (2)
C11	0.045 (2)	0.059 (3)	0.086 (3)	−0.004 (2)	0.012 (2)	−0.009 (3)
C12	0.085 (4)	0.096 (4)	0.076 (3)	−0.018 (3)	0.005 (3)	−0.011 (3)
C13	0.070 (3)	0.123 (5)	0.079 (4)	−0.018 (4)	0.001 (3)	−0.021 (4)
C14	0.094 (5)	0.217 (10)	0.119 (6)	0.019 (7)	−0.014 (5)	−0.054 (6)
C15	0.122 (8)	0.273 (16)	0.143 (8)	0.033 (9)	−0.043 (8)	−0.053 (10)
C16	0.072 (5)	0.298 (17)	0.145 (8)	0.019 (7)	0.000 (6)	−0.093 (10)
C17	0.120 (6)	0.242 (13)	0.117 (6)	−0.059 (8)	0.031 (5)	−0.036 (7)
C18	0.090 (4)	0.170 (7)	0.089 (4)	−0.026 (5)	0.020 (4)	−0.044 (5)
C19	0.157 (7)	0.144 (7)	0.143 (7)	0.001 (7)	0.042 (6)	0.017 (6)
C20	0.146 (7)	0.152 (8)	0.128 (6)	0.013 (7)	0.004 (6)	−0.005 (6)
C21	0.085 (4)	0.160 (8)	0.130 (5)	0.001 (5)	0.003 (4)	−0.029 (6)

### (9) (S)-(–)-2-{[(1,2,3,4-Tetrahydronaphthalen-1-yl)imino]methyl}naphthalene Geometric parameters (Å, º)

**Table:**

N1—C11	1.265 (5)	C12—C13	1.491 (7)
N1—C12	1.457 (6)	C12—C21	1.527 (9)
C1—C2	1.360 (6)	C12—H12A	0.9800
C1—C10	1.422 (6)	C13—C14	1.363 (9)
C1—C11	1.469 (6)	C13—C18	1.430 (9)
C2—C3	1.413 (5)	C14—C15	1.436 (12)
C2—H2A	0.9300	C14—H14A	0.9300
C3—C4	1.407 (6)	C15—C16	1.312 (14)
C3—C8	1.424 (6)	C15—H15A	0.9300
C4—C5	1.360 (6)	C16—C17	1.386 (15)
C4—H4A	0.9300	C16—H16A	0.9300
C5—C6	1.413 (8)	C17—C18	1.369 (9)
C5—H5A	0.9300	C17—H17A	0.9300
C6—C7	1.359 (7)	C18—C19	1.517 (10)
C6—H6A	0.9300	C19—C20	1.475 (10)
C7—C8	1.415 (6)	C19—H19A	0.9700
C7—H7A	0.9300	C19—H19B	0.9700
C8—C9	1.412 (6)	C20—C21	1.454 (11)
C9—C10	1.352 (6)	C20—H20A	0.9700
C9—H9A	0.9300	C20—H20B	0.9700
C10—H10A	0.9300	C21—H21A	0.9700
C11—H11A	0.9300	C21—H21B	0.9700
			
C11—N1—C12	118.2 (5)	C13—C12—H12A	108.9
C2—C1—C10	119.2 (4)	C21—C12—H12A	108.9
C2—C1—C11	120.9 (4)	C14—C13—C18	121.2 (7)
C10—C1—C11	119.9 (4)	C14—C13—C12	119.7 (7)
C1—C2—C3	121.9 (4)	C18—C13—C12	119.0 (7)
C1—C2—H2A	119.0	C13—C14—C15	119.6 (9)
C3—C2—H2A	119.0	C13—C14—H14A	120.2
C4—C3—C2	123.0 (4)	C15—C14—H14A	120.2
C4—C3—C8	118.7 (4)	C16—C15—C14	117.2 (11)
C2—C3—C8	118.3 (4)	C16—C15—H15A	121.4
C5—C4—C3	121.4 (5)	C14—C15—H15A	121.4
C5—C4—H4A	119.3	C15—C16—C17	125.2 (11)
C3—C4—H4A	119.3	C15—C16—H16A	117.4
C4—C5—C6	119.8 (5)	C17—C16—H16A	117.4
C4—C5—H5A	120.1	C18—C17—C16	119.2 (10)
C6—C5—H5A	120.1	C18—C17—H17A	120.4
C7—C6—C5	120.6 (5)	C16—C17—H17A	120.4
C7—C6—H6A	119.7	C17—C18—C13	117.6 (9)
C5—C6—H6A	119.7	C17—C18—C19	120.8 (9)
C6—C7—C8	120.7 (5)	C13—C18—C19	121.4 (6)
C6—C7—H7A	119.6	C20—C19—C18	114.0 (7)
C8—C7—H7A	119.6	C20—C19—H19A	108.7
C9—C8—C7	122.6 (5)	C18—C19—H19A	108.7
C9—C8—C3	118.6 (4)	C20—C19—H19B	108.7
C7—C8—C3	118.8 (4)	C18—C19—H19B	108.7
C10—C9—C8	121.4 (5)	H19A—C19—H19B	107.6
C10—C9—H9A	119.3	C21—C20—C19	113.0 (8)
C8—C9—H9A	119.3	C21—C20—H20A	109.0
C9—C10—C1	120.5 (4)	C19—C20—H20A	109.0
C9—C10—H10A	119.8	C21—C20—H20B	109.0
C1—C10—H10A	119.8	C19—C20—H20B	109.0
N1—C11—C1	122.8 (5)	H20A—C20—H20B	107.8
N1—C11—H11A	118.6	C20—C21—C12	111.4 (6)
C1—C11—H11A	118.6	C20—C21—H21A	109.3
N1—C12—C13	108.5 (4)	C12—C21—H21A	109.3
N1—C12—C21	109.0 (5)	C20—C21—H21B	109.3
C13—C12—C21	112.6 (5)	C12—C21—H21B	109.3
N1—C12—H12A	108.9	H21A—C21—H21B	108.0
			
C10—C1—C2—C3	−2.3 (6)	C11—N1—C12—C13	−115.5 (6)
C11—C1—C2—C3	175.3 (3)	C11—N1—C12—C21	121.6 (6)
C1—C2—C3—C4	−177.9 (4)	N1—C12—C13—C14	80.6 (7)
C1—C2—C3—C8	1.1 (5)	C21—C12—C13—C14	−158.7 (6)
C2—C3—C4—C5	177.1 (4)	N1—C12—C13—C18	−95.9 (6)
C8—C3—C4—C5	−2.0 (6)	C21—C12—C13—C18	24.9 (8)
C3—C4—C5—C6	2.0 (6)	C18—C13—C14—C15	0.9 (10)
C4—C5—C6—C7	−0.8 (7)	C12—C13—C14—C15	−175.5 (7)
C5—C6—C7—C8	−0.4 (7)	C13—C14—C15—C16	−1.4 (13)
C6—C7—C8—C9	−178.5 (4)	C14—C15—C16—C17	1.3 (16)
C6—C7—C8—C3	0.3 (6)	C15—C16—C17—C18	−0.6 (15)
C4—C3—C8—C9	179.7 (4)	C16—C17—C18—C13	0.0 (11)
C2—C3—C8—C9	0.6 (5)	C16—C17—C18—C19	−176.3 (7)
C4—C3—C8—C7	0.8 (5)	C14—C13—C18—C17	−0.2 (9)
C2—C3—C8—C7	−178.3 (4)	C12—C13—C18—C17	176.2 (6)
C7—C8—C9—C10	177.7 (4)	C14—C13—C18—C19	176.1 (6)
C3—C8—C9—C10	−1.1 (6)	C12—C13—C18—C19	−7.5 (8)
C8—C9—C10—C1	−0.1 (6)	C17—C18—C19—C20	−168.6 (7)
C2—C1—C10—C9	1.8 (6)	C13—C18—C19—C20	15.2 (10)
C11—C1—C10—C9	−175.9 (4)	C18—C19—C20—C21	−42.0 (10)
C12—N1—C11—C1	176.6 (4)	C19—C20—C21—C12	61.1 (9)
C2—C1—C11—N1	−180.0 (4)	N1—C12—C21—C20	69.0 (7)
C10—C1—C11—N1	−2.3 (6)	C13—C12—C21—C20	−51.5 (8)

### (10) (+)-2-({[(1S,2S,3S,5R)-2,6,6-Trimethylbicyclo[3.1.1]hept-3-yl]imino}methyl}naphthalene Crystal data

**Table:**

C_21_H_25_N	*D*_x_ = 1.142 Mg m^−^^3^
*M**_r_* = 291.42	Melting point: 373 K
Orthorhombic, *P*2_1_2_1_2_1_	Cu *K*α radiation, λ = 1.54184 Å
*a* = 6.32427 (18) Å	Cell parameters from 4989 reflections
*b* = 14.4559 (3) Å	θ = 3.9–73.9°
*c* = 18.5421 (5) Å	µ = 0.49 mm^−^^1^
*V* = 1695.17 (8) Å^3^	*T* = 150 K
*Z* = 4	Prism, colorless
*F*(000) = 632	0.40 × 0.15 × 0.12 mm

### (10) (+)-2-({[(1S,2S,3S,5R)-2,6,6-Trimethylbicyclo[3.1.1]hept-3-yl]imino}methyl}naphthalene Data collection

**Table:**

Agilent Xcalibur Atlas Gemini diffractometer	3417 independent reflections
Radiation source: Enhance (Cu) X-ray Source	2913 reflections with *I* > 2σ(*I*)
Graphite monochromator	*R*_int_ = 0.056
Detector resolution: 10.5564 pixels mm^-1^	θ_max_ = 74.1°, θ_min_ = 3.9°
ω scans	*h* = −7→7
Absorption correction: multi-scan (CrysAlis PRO; Agilent, 2013)	*k* = −18→17
*T*_min_ = 0.976, *T*_max_ = 1.000	*l* = −23→22
19648 measured reflections	

### (10) (+)-2-({[(1S,2S,3S,5R)-2,6,6-Trimethylbicyclo[3.1.1]hept-3-yl]imino}methyl}naphthalene Refinement

**Table:**

Refinement on *F*^2^	Primary atom site location: structure-invariant direct methods
Least-squares matrix: full	Secondary atom site location: difference Fourier map
*R*[*F*^2^ > 2σ(*F*^2^)] = 0.041	Hydrogen site location: inferred from neighbouring sites
*wR*(*F*^2^) = 0.103	H-atom parameters constrained
*S* = 1.03	*w* = 1/[σ^2^(*F*_o_^2^) + (0.0482*P*)^2^ + 0.2349*P*] where *P* = (*F*_o_^2^ + 2*F*_c_^2^)/3
3417 reflections	(Δ/σ)_max_ < 0.001
202 parameters	Δρ_max_ = 0.19 e Å^−^^3^
0 restraints	Δρ_min_ = −0.17 e Å^−^^3^
0 constraints	

### (10) (+)-2-({[(1S,2S,3S,5R)-2,6,6-Trimethylbicyclo[3.1.1]hept-3-yl]imino}methyl}naphthalene Fractional atomic coordinates and isotropic or equivalent isotropic displacement parameters (Å^2^)

**Table:**

	*x*	*y*	*z*	*U*_iso_*/*U*_eq_	
N1	0.1655 (3)	0.02849 (13)	0.12338 (10)	0.0287 (4)	
C1	0.1959 (4)	0.19008 (16)	0.09368 (12)	0.0275 (5)	
C2	0.0953 (4)	0.26587 (16)	0.06545 (12)	0.0278 (5)	
H2A	−0.0418	0.2590	0.0452	0.033*	
C3	0.1928 (4)	0.35464 (16)	0.06593 (12)	0.0283 (5)	
C4	0.0890 (5)	0.43469 (17)	0.03906 (13)	0.0347 (6)	
H4A	−0.0478	0.4294	0.0184	0.042*	
C5	0.1852 (5)	0.51971 (18)	0.04284 (14)	0.0408 (7)	
H5A	0.1139	0.5730	0.0253	0.049*	
C6	0.3883 (5)	0.52811 (18)	0.07245 (14)	0.0419 (7)	
H6A	0.4527	0.5874	0.0752	0.050*	
C7	0.4950 (5)	0.45229 (18)	0.09736 (13)	0.0370 (6)	
H7A	0.6337	0.4592	0.1163	0.044*	
C8	0.3999 (4)	0.36339 (16)	0.09509 (12)	0.0293 (5)	
C9	0.5017 (4)	0.28340 (17)	0.12220 (13)	0.0318 (5)	
H9A	0.6411	0.2884	0.1409	0.038*	
C10	0.4039 (4)	0.19941 (17)	0.12208 (12)	0.0303 (5)	
H10A	0.4749	0.1468	0.1410	0.036*	
C11	0.0855 (4)	0.10034 (16)	0.09522 (12)	0.0276 (5)	
H11A	−0.0512	0.0961	0.0743	0.033*	
C12	0.0344 (4)	−0.05514 (15)	0.12429 (12)	0.0272 (5)	
H12A	−0.0933	−0.0432	0.0939	0.033*	
C13	−0.0418 (4)	−0.07285 (16)	0.20294 (12)	0.0319 (5)	
H13A	0.0611	−0.0407	0.2354	0.038*	
C14	−0.0318 (5)	−0.17642 (17)	0.22220 (13)	0.0349 (6)	
H14A	−0.0942	−0.1927	0.2701	0.042*	
C15	−0.0922 (4)	−0.24379 (16)	0.15923 (13)	0.0314 (5)	
C16	0.1384 (4)	−0.22704 (16)	0.13233 (14)	0.0312 (5)	
H16A	0.2087	−0.2818	0.1098	0.037*	
C17	0.1577 (4)	−0.13693 (16)	0.09012 (13)	0.0323 (5)	
H17A	0.1047	−0.1471	0.0405	0.039*	
H17B	0.3090	−0.1199	0.0867	0.039*	
C18	0.2002 (5)	−0.20788 (18)	0.21145 (15)	0.0394 (6)	
H18A	0.3060	−0.1580	0.2176	0.047*	
H18B	0.2396	−0.2638	0.2392	0.047*	
C19	−0.2562 (5)	−0.0283 (2)	0.21626 (17)	0.0468 (7)	
H19A	−0.2508	0.0370	0.2022	0.070*	
H19B	−0.2918	−0.0330	0.2676	0.070*	
H19C	−0.3640	−0.0602	0.1876	0.070*	
C20	−0.1237 (5)	−0.34257 (18)	0.18812 (16)	0.0450 (7)	
H20A	−0.0073	−0.3583	0.2208	0.068*	
H20B	−0.1256	−0.3862	0.1477	0.068*	
H20C	−0.2581	−0.3462	0.2142	0.068*	
C21	−0.2736 (4)	−0.22375 (19)	0.10794 (16)	0.0408 (7)	
H21A	−0.2636	−0.1597	0.0909	0.061*	
H21B	−0.4083	−0.2327	0.1331	0.061*	
H21C	−0.2659	−0.2659	0.0666	0.061*	

### (10) (+)-2-({[(1S,2S,3S,5R)-2,6,6-Trimethylbicyclo[3.1.1]hept-3-yl]imino}methyl}naphthalene Atomic displacement parameters (Å^2^)

**Table:**

	*U*^11^	*U*^22^	*U*^33^	*U*^12^	*U*^13^	*U*^23^
N1	0.0296 (11)	0.0260 (9)	0.0304 (9)	−0.0025 (8)	0.0004 (9)	0.0014 (8)
C1	0.0312 (13)	0.0279 (11)	0.0235 (10)	−0.0026 (10)	0.0044 (9)	−0.0008 (9)
C2	0.0284 (12)	0.0291 (11)	0.0260 (10)	−0.0010 (10)	0.0007 (9)	−0.0015 (8)
C3	0.0348 (14)	0.0273 (11)	0.0229 (10)	−0.0009 (10)	0.0071 (10)	−0.0025 (9)
C4	0.0411 (15)	0.0308 (12)	0.0322 (12)	0.0025 (12)	0.0068 (11)	0.0012 (9)
C5	0.0575 (19)	0.0277 (12)	0.0371 (13)	0.0035 (13)	0.0123 (13)	0.0020 (11)
C6	0.060 (2)	0.0280 (12)	0.0378 (13)	−0.0116 (13)	0.0161 (13)	−0.0031 (10)
C7	0.0407 (15)	0.0393 (14)	0.0309 (11)	−0.0142 (12)	0.0090 (11)	−0.0048 (10)
C8	0.0336 (13)	0.0311 (12)	0.0230 (9)	−0.0051 (10)	0.0067 (10)	−0.0025 (9)
C9	0.0286 (12)	0.0386 (13)	0.0282 (11)	−0.0035 (11)	−0.0001 (10)	−0.0008 (10)
C10	0.0311 (13)	0.0315 (11)	0.0285 (10)	−0.0002 (10)	−0.0004 (11)	0.0031 (9)
C11	0.0296 (12)	0.0293 (11)	0.0239 (9)	−0.0019 (10)	0.0009 (9)	−0.0026 (9)
C12	0.0280 (12)	0.0255 (10)	0.0281 (10)	−0.0003 (9)	−0.0011 (10)	0.0005 (9)
C13	0.0397 (14)	0.0274 (11)	0.0285 (11)	−0.0049 (11)	0.0032 (10)	−0.0029 (9)
C14	0.0483 (16)	0.0284 (11)	0.0279 (11)	−0.0068 (11)	0.0007 (11)	0.0033 (9)
C15	0.0333 (14)	0.0250 (11)	0.0359 (12)	−0.0027 (10)	0.0016 (11)	−0.0017 (9)
C16	0.0299 (13)	0.0251 (11)	0.0388 (12)	0.0015 (10)	−0.0027 (11)	−0.0015 (9)
C17	0.0326 (14)	0.0300 (12)	0.0342 (12)	0.0008 (10)	0.0057 (10)	−0.0019 (10)
C18	0.0443 (16)	0.0305 (12)	0.0433 (14)	−0.0023 (11)	−0.0163 (13)	0.0074 (11)
C19	0.0514 (19)	0.0365 (14)	0.0526 (16)	−0.0018 (13)	0.0220 (14)	−0.0080 (13)
C20	0.0529 (19)	0.0296 (13)	0.0526 (15)	−0.0079 (13)	0.0056 (14)	0.0004 (11)
C21	0.0316 (14)	0.0349 (14)	0.0560 (17)	−0.0052 (12)	−0.0066 (12)	−0.0088 (12)

### (10) (+)-2-({[(1S,2S,3S,5R)-2,6,6-Trimethylbicyclo[3.1.1]hept-3-yl]imino}methyl}naphthalene Geometric parameters (Å, º)

**Table:**

N1—C11	1.268 (3)	C13—C19	1.521 (4)
N1—C12	1.466 (3)	C13—C14	1.541 (3)
C1—C2	1.371 (3)	C13—H13A	1.0000
C1—C10	1.424 (3)	C14—C18	1.549 (4)
C1—C11	1.473 (3)	C14—C15	1.568 (3)
C2—C3	1.424 (3)	C14—H14A	1.0000
C2—H2A	0.9500	C15—C21	1.518 (4)
C3—C4	1.421 (4)	C15—C20	1.538 (3)
C3—C8	1.423 (4)	C15—C16	1.560 (4)
C4—C5	1.373 (4)	C16—C17	1.525 (3)
C4—H4A	0.9500	C16—C18	1.543 (4)
C5—C6	1.402 (5)	C16—H16A	1.0000
C5—H5A	0.9500	C17—H17A	0.9900
C6—C7	1.368 (4)	C17—H17B	0.9900
C6—H6A	0.9500	C18—H18A	0.9900
C7—C8	1.419 (3)	C18—H18B	0.9900
C7—H7A	0.9500	C19—H19A	0.9800
C8—C9	1.416 (4)	C19—H19B	0.9800
C9—C10	1.363 (3)	C19—H19C	0.9800
C9—H9A	0.9500	C20—H20A	0.9800
C10—H10A	0.9500	C20—H20B	0.9800
C11—H11A	0.9500	C20—H20C	0.9800
C12—C17	1.552 (3)	C21—H21A	0.9800
C12—C13	1.557 (3)	C21—H21B	0.9800
C12—H12A	1.0000	C21—H21C	0.9800
			
C11—N1—C12	117.0 (2)	C13—C14—C15	114.9 (2)
C2—C1—C10	119.6 (2)	C18—C14—C15	87.28 (19)
C2—C1—C11	119.4 (2)	C13—C14—H14A	114.8
C10—C1—C11	120.9 (2)	C18—C14—H14A	114.8
C1—C2—C3	121.1 (2)	C15—C14—H14A	114.8
C1—C2—H2A	119.4	C21—C15—C20	107.3 (2)
C3—C2—H2A	119.4	C21—C15—C16	118.4 (2)
C4—C3—C8	119.1 (2)	C20—C15—C16	112.1 (2)
C4—C3—C2	122.1 (2)	C21—C15—C14	122.2 (2)
C8—C3—C2	118.8 (2)	C20—C15—C14	110.4 (2)
C5—C4—C3	120.4 (3)	C16—C15—C14	85.06 (19)
C5—C4—H4A	119.8	C17—C16—C18	108.3 (2)
C3—C4—H4A	119.8	C17—C16—C15	111.8 (2)
C4—C5—C6	120.2 (3)	C18—C16—C15	87.76 (19)
C4—C5—H5A	119.9	C17—C16—H16A	115.3
C6—C5—H5A	119.9	C18—C16—H16A	115.3
C7—C6—C5	121.0 (3)	C15—C16—H16A	115.3
C7—C6—H6A	119.5	C16—C17—C12	113.7 (2)
C5—C6—H6A	119.5	C16—C17—H17A	108.8
C6—C7—C8	120.4 (3)	C12—C17—H17A	108.8
C6—C7—H7A	119.8	C16—C17—H17B	108.8
C8—C7—H7A	119.8	C12—C17—H17B	108.8
C9—C8—C7	122.4 (2)	H17A—C17—H17B	107.7
C9—C8—C3	118.7 (2)	C16—C18—C14	86.27 (19)
C7—C8—C3	118.8 (2)	C16—C18—H18A	114.3
C10—C9—C8	121.4 (2)	C14—C18—H18A	114.3
C10—C9—H9A	119.3	C16—C18—H18B	114.3
C8—C9—H9A	119.3	C14—C18—H18B	114.3
C9—C10—C1	120.3 (2)	H18A—C18—H18B	111.4
C9—C10—H10A	119.8	C13—C19—H19A	109.5
C1—C10—H10A	119.8	C13—C19—H19B	109.5
N1—C11—C1	122.7 (2)	H19A—C19—H19B	109.5
N1—C11—H11A	118.6	C13—C19—H19C	109.5
C1—C11—H11A	118.6	H19A—C19—H19C	109.5
N1—C12—C17	109.8 (2)	H19B—C19—H19C	109.5
N1—C12—C13	108.75 (18)	C15—C20—H20A	109.5
C17—C12—C13	114.37 (19)	C15—C20—H20B	109.5
N1—C12—H12A	107.9	H20A—C20—H20B	109.5
C17—C12—H12A	107.9	C15—C20—H20C	109.5
C13—C12—H12A	107.9	H20A—C20—H20C	109.5
C19—C13—C14	114.2 (2)	H20B—C20—H20C	109.5
C19—C13—C12	111.0 (2)	C15—C21—H21A	109.5
C14—C13—C12	111.37 (19)	C15—C21—H21B	109.5
C19—C13—H13A	106.6	H21A—C21—H21B	109.5
C14—C13—H13A	106.6	C15—C21—H21C	109.5
C12—C13—H13A	106.6	H21A—C21—H21C	109.5
C13—C14—C18	107.1 (2)	H21B—C21—H21C	109.5
			
C10—C1—C2—C3	−1.9 (3)	N1—C12—C13—C14	−138.8 (2)
C11—C1—C2—C3	177.1 (2)	C17—C12—C13—C14	−15.7 (3)
C1—C2—C3—C4	−178.0 (2)	C19—C13—C14—C18	−175.4 (2)
C1—C2—C3—C8	1.3 (3)	C12—C13—C14—C18	57.9 (3)
C8—C3—C4—C5	−1.6 (3)	C19—C13—C14—C15	89.6 (3)
C2—C3—C4—C5	177.6 (2)	C12—C13—C14—C15	−37.1 (3)
C3—C4—C5—C6	0.8 (4)	C13—C14—C15—C21	−40.3 (4)
C4—C5—C6—C7	0.6 (4)	C18—C14—C15—C21	−147.9 (2)
C5—C6—C7—C8	−1.3 (4)	C13—C14—C15—C20	−167.8 (2)
C6—C7—C8—C9	−178.1 (2)	C18—C14—C15—C20	84.6 (3)
C6—C7—C8—C3	0.5 (3)	C13—C14—C15—C16	80.4 (2)
C4—C3—C8—C9	179.6 (2)	C18—C14—C15—C16	−27.21 (17)
C2—C3—C8—C9	0.3 (3)	C21—C15—C16—C17	42.6 (3)
C4—C3—C8—C7	0.9 (3)	C20—C15—C16—C17	168.4 (2)
C2—C3—C8—C7	−178.3 (2)	C14—C15—C16—C17	−81.6 (2)
C7—C8—C9—C10	177.3 (2)	C21—C15—C16—C18	151.4 (2)
C3—C8—C9—C10	−1.3 (3)	C20—C15—C16—C18	−82.7 (2)
C8—C9—C10—C1	0.7 (3)	C14—C15—C16—C18	27.31 (17)
C2—C1—C10—C9	0.9 (3)	C18—C16—C17—C12	−53.0 (3)
C11—C1—C10—C9	−178.1 (2)	C15—C16—C17—C12	42.1 (3)
C12—N1—C11—C1	177.13 (19)	N1—C12—C17—C16	136.0 (2)
C2—C1—C11—N1	−176.8 (2)	C13—C12—C17—C16	13.4 (3)
C10—C1—C11—N1	2.2 (3)	C17—C16—C18—C14	84.6 (2)
C11—N1—C12—C17	126.8 (2)	C15—C16—C18—C14	−27.62 (17)
C11—N1—C12—C13	−107.3 (2)	C13—C14—C18—C16	−87.8 (2)
N1—C12—C13—C19	92.7 (2)	C15—C14—C18—C16	27.48 (17)
C17—C12—C13—C19	−144.1 (2)		
