# Supplementary material for: Crystal structures of ten enanti­opure Schiff bases bearing a naphthyl group
Source: Acta Crystallogr E Crystallogr Commun. 2016 Mar 31;72(Pt 4):583–9. doi: 10.1107/S2056989016004692 (PMC4910349; doi:10.1107/S2056989016004692)
Supplement: Supplementary file 22 [file e-72-00583-sup22.pdf]

Crystal structures of ten enantiopure Schiff bases bearing a naphthyl group

Guadalupe Hernández-Téllez,[a]* Gloria E. Moreno,[a] Sylvain Bernès,[b] Angel Mendoza,[c] Oscar Portillo,[a] Pankaj Sharma[d] and René Gutiérrez[a]

[a] Laboratorio de Síntesis de Complejos, Facultad de Ciencias Químicas, Universidad Autónoma de Puebla, A.P. 1067, 72001 Puebla, Pue., Mexico

[b] Instituto de Física, Benemérita Universidad Autónoma de Puebla, Av. San Claudio y 18 Sur, 72570 Puebla, Pue., Mexico

[c] Centro de Química, Instituto de Ciencias, Benemérita Universidad Autónoma de Puebla, 72570 Puebla, Pue., Mexico

[d] Instituto de Química, Universidad Nacional Autónoma de México, Circuito Exterior s/n, Ciudad Universitaria, Delegación Coyoacán, 04510 México D.F., Mexico

SUPPORTING INFORMATION

General Methods

^{1}H NMR and ^{13}C NMR spectra were recorded on a Bruker-500MHz apparatus, using $CDCl_3$ as solvent and TMS as internal reference. IR spectra were performed on a Perkin Elmer Spectrum One FT-IR spectrometer Universal ATR. The electronic impact (EI) ionization mass spectra were acquired on a Jeol JMS-SX 102A mass spectrometer operated in the positive ion mode. The acquisition conditions were ion source temperature 230°C, ionization energy 70 eV, emission current 0.14 μA and ionization current 100 μA. Melting points were measured using a Mel-Temp II apparatus and are uncorrected. The optical rotation was obtained on a Perkin Elmer 241 polarimeter. Elemental analyses were obtained from a Euro EA elemental analyzer. Reagents were obtained from commercial suppliers and used as received.

General synthesis of compounds **1-10**

Under solvent-free conditions, 2-naphthaldehyde (0.25 g, 1.6 mmol) was mixed in equimolar amounts with the chiral amine, *i.e.* with (*S*)-(−)-1-phenylethylamine (0.19 g, 1.6 mmol), (*S*)-(−)-1-(4-methylphenyl)ethylamine (0.21 g, 1.6 mmol), (*R*)-(+)-1-(4-methoxylphenyl)ethylamine (0.24 g, 1.6 mmol), (*R*)-(+)-1-(4-fluorophenyl)ethylamine (0.22 g, 1.6 mmol), (*S*)-(−)-1-(4-chlorophenyl)ethylamine (0.25 g, 1.6 mmol), (*S*)-(−)-1-(4-bromophenyl)ethylamine (0.32 g, 1.6 mmol), (*S*)-(−)-1-(1-naphthyl)ethylamine (0.27 g, 1.6 mmol), (*S*)-(+)-1-cyclohexylethylamine (0.20 g, 1.6 mmol), (*S*)-(+)-1,2,3,4-tetrahydro-1-naphthylamine (0.23 g, 1.6 mmol), and (1*S*,2*S*,3*S*,5*R*)-(+)-(2,6,6-trimethylbicyclo[3.1.1]hept-3-yl)amine (0.24 g, 1.6 mmol), respectively, at room temperature. The reaction was monitored by thin-layer chromatography and ^{1}H NMR. After the reaction was completed (~ 3-4 min.), the resultant white solids were allowed to dry in air (~ 2-3 min.) to give **1-10** with excellent yields (>99%), based on a mass of crude product obtained.

Imine **1**: **(*S*)-(+)-2-(((1-Phenylethyl)imino)methyl)naphthalene.** Mp 116 °C. FT-IR: ν=1629 cm^{-1} (C=N). ^{1}H NMR (500 MHz, $CDCl_3$/TMS): δ=8.51 (s, 1H; *H*C=N), 8.05-8.08 (m, 2H; Ar-*H*), 7.83-7.89 (m, 3H; Ar-*H*), 7.46-7.52 (m, 4H; Ar-*H*), 7.34-7.37 (m, 2H; Ar-*H*), 7.23-7.27 (m, 1H; Ar-*H*), 4.60 (q, 1H; C*H*), 1.63 (d, 3H; $CHCH_3$,); ^{13}C NMR (500 MHz, $CDCl_3$/TMS): δ=159.62 (H*C*=N), 145.23, 134.72, 134.13, 133.12, 129.98, 128.62, 128.50, 128.42, 127.91, 127.09, 126.91, 126.73, 126.43, 124.15 (*C*-Ar), 69.87 ($CHCH_3$), 24.96 ($CHCH_3$) ppm. MS-EI m/z=259 (M^{+}). $[\alpha]_D^{20}$= +60.7 ($CHCl_3$). Anal. Calcd. for $C_{19}H_{17}N$: C, 87.99; H, 6.61; N, 5.40. Found: C, 87.43; H, 6.33; N, 5.22.

Imine **2**: **(*S*)-(+)-2-((((4-Methylphenyl)ethyl)imino)methyl)naphthalene.** Mp 124 °C. FT-IR: ν=1629 cm^{-1} (C=N). ^{1}H NMR (500 MHz, $CDCl_3$/TMS): δ=8.51 (s, 1H; *H*C=N), 8.05-8.07 (m, 2H; Ar-*H*), 7.84-7.89 (m, 3H; Ar-*H*), 7.48-7.52 (m, 2H; Ar-*H*), 7.35 (d, 2H; Ar-*H*), 7.17 (d, 2H; Ar-*H*), 4.58 (q, 1H; C*H*), 2.34 (s, 3H; CH_3), 1.62 (d, 3H; $CHCH_3$); ^{13}C NMR (500 MHz, $CDCl_3$/TMS): δ=159.43 (H*C*=N), 142.20, 136.47, 134.68, 134.17,

133.11, 129.89, 129.16, 128.59, 128.36, 127.88, 127.03, 126.62, 126.38, 124.17 (*C*-Ar), 69.59 (*C*HCH$_3$), 24.83 (CH*C*H$_3$), 21.11 (Ar*C*H$_3$) ppm. MS-EI *m*/*z*=273 (M^+). $[\alpha]_D^{20}$= +40.2. Anal. Calcd. for $C_{20}H_{19}N$: C, 87.87; H, 7.01; N, 5.12. Found: C, 87.59; H, 6.98; N, 5.03.

Imine **3**: **(*R*)-(–)-2-((((4-Methoxylphenyl)ethyl)imino)methyl)naphthalene.** Mp 111 °C. FT-IR: ν=1633 cm^{-1} (C=N). ^{1}H NMR (500 MHz, $CDCl_3$/TMS): δ=8.50 (s, 1H; *H*C=N), 8.04-8.06 (m, 2H; Ar-*H*), 7.83-7.89 (m, 3H; Ar-*H*), 7.48-7.53 (m, 2H; Ar-*H*), 7.38 (d, 2H; Ar-*H*), 6.90 (d, 2H; Ar-*H*), 4.57 (q, 1H; C*H*CH$_3$), 3.80 (s, 3H; OC*H*$_3$), 1.61 (d, 3H; C*H*$_3$); ^{13}C NMR (500 MHz, $CDCl_3$/TMS): δ=159.34 (H*C*=N), 158.52, 137.31, 134.68, 134.15, 133.11, 129.89, 128.59, 128.37, 127.88, 127.75, 127.04, 126.40, 124.15, 113.85 (*C*-Ar), 69.20 (*C*HCH$_3$), 55.32 (*OC*H$_3$), 24.79 (CH*C*H$_3$) ppm. MS-EI *m*/*z* = 289 (M^+). $[\alpha]_D^{20}$= -22.7. Anal. Calcd. for $C_{20}H_{19}NO$: C, 83.01; H, 6.62; N, 4.84. Found: C, 82.88; H, 6.57; N, 4.80.

Imine **4**: **(*R*)-(–)-2-((((4-Fluorophenyl)ethyl)imino)methyl)naphthalene.** Mp 122 °C. FT-IR: ν=1631 cm^{-1} (C=N). ^{1}H NMR (500 MHz, $CDCl_3$/TMS): δ=8.50 (s,1H; *H*C=N), 8.05-8.07 (m, 2H; Ar-*H*), 7.83-7.89 (m, 3H; Ar-*H*), 7.48-7.53 (m, 2H; Ar-*H*), 7.41-7.45 (m, 2H; Ar-*H*), 7.01-7.06 (m, 2H; Ar-*H*), 4.58 (q, 1H; C*H*CH$_3$), 1.60 (d, 3H; C*H*$_3$); ^{13}C NMR (500 MHz, $CDCl_3$/TMS): δ=159.69 (H*C*=N), 161.81 (d, $J_{F\text{-}C}$=244.89 Hz, *C*-Ar), 140.95 (d, $J_{F\text{-}C}$ =3.77 Hz, *C*-Ar), 134.75, 133.99, 133.10, 130.05, 128.62, 128.46, 128.17 (d, $J_{F\text{-}C}$=7.54 Hz, *C*-Ar), 127.91, 127.16, 126.48, 124.05, 115.21 (d, $J_{F\text{-}C}$=21.3 Hz, *C*-Ar), 69.13 (*C*HCH$_3$), 25.10 (CH*C*H$_3$) ppm. MS-EI *m*/*z* = 277 (M^+). $[\alpha]_D^{20}$= -45.8. Anal. Calcd. for $C_{19}H_{16}NF$: C, 82.28; H, 5.81; N, 5.05. Found: C, 82.15; H, 5.77; N, 4.94.

Imine **5**: **(*S*)-(+)-2-((((4-Chlorophenyl)ethyl)imino)methyl)naphthalene.** Mp 127 °C. FT-IR: ν=1627 cm^{-1} (C=N). ^{1}H NMR (500 MHz, $CDCl_3$/TMS): δ=8.51 (s,1H; *H*C=N), 8.05-8.07 (m, 2H; Ar-*H*), 7.83-7.90 (m, 3H; Ar-*H*), 7.48-7.54 (m, 2H; Ar-*H*), 7.39-7.42 (m, 2H; Ar-*H*), 7.31-7.33 (m, 2H; Ar-*H*), 4.57 (q, 1H; C*H*CH$_3$), 1.59 (d, 3H; C*H*$_3$); ^{13}C NMR (500 MHz, $CDCl_3$/TMS): δ=159.87 (H*C*=N), 143.76, 134.76, 133.93, 133.09, 132.50, 130.09, 128.62, 128.57, 128.47, 128.07, 127.91, 127.18, 126.49, 124.01 (*C*-Ar), 69.15

(CHCH$_3$), 25.04 (CHCH$_3$) ppm. MS-EI *m*/*z* = 293 (M^+). $[\alpha]_D^{20}$= +60.9. Anal. Calcd. for $C_{19}H_{16}NCl$: C, 77.68; H, 5.49; N, 4.77. Found: C, 77.51; H, 5.38; N, 4.62.

Imine **6**: **(*S*)-(+)-2-((((4-Bromophenyl)ethyl)imino)methyl)naphthalene.** Mp 142 °C. FT-IR: ν=1626 cm^{-1} (C=N). ^{1}H NMR (500 MHz, $CDCl_3$/TMS): δ=8.51 (s, 1H; *H*C=N), 8.05-8.07 (m, 2H; Ar-*H*), 7.84-7.90 (m, 3H; Ar-*H*), 7.46-7.54 (m, 4H; Ar-*H*), 7.34-7.36 (m, 2H; Ar-*H*), 4.55 (q, 1H; C*H*CH$_3$), 1.59 (d, 3H; CH_3); ^{13}C NMR (500 MHz, $CDCl_3$/TMS): δ=159.90 (H*C*=N), 144.30, 134.76, 133.91, 133.09, 131.52, 130.09, 128.62, 128.46, 127.91, 127.18, 126.49, 124.00, 120.61 (*C*-Ar), 69.20 (*C*HCH$_3$), 25.01 (CH*C*H$_3$) ppm. MS-EI *m*/*z* = 337 (M^+). $[\alpha]_D^{20}$= +27.6. Anal. Calcd. for $C_{19}H_{16}NBr$: C, 67.47; H, 4.77; N, 4.14. Found: C, 67.38; H, 4.65; N, 3.99.

Imine **7**: **(*S*)-(+)-2-((((1-Naphthyl)ethyl)imino)methyl)naphthalene.** Mp 131°C. FT-IR: ν=1635 cm^{-1} (C=N). ^{1}H NMR (500 MHz, $CDCl_3$/TMS): δ=8.57 (1H; *H*C=N), 8.28 (d, 1H; Ar-*H*), 8.13 (d, 1H; Ar-*H*), 8.04 (s, 1H; Ar-*H*), 7.84-7.89 (m, 5H; Ar-*H*), 7.77 (d, 1H; Ar-*H*), 7.47-7.56 (m, 5H; Ar-*H*), 5.43 (q, 1H; C*H*), 1.78 (d, 3H; CHCH_3); ^{13}C NMR (500 MHz, $CDCl_3$/TMS): δ=159.79 (H*C*=N), 141.17, 134.72, 134.21, 134.02, 133.11, 130.71, 130.07, 128.98, 128.61, 128.43, 127.89, 127.40, 127.09, 126.42, 125.87, 125.75, 125.37, 124.11, 124.08, 123.66 (*C*-Ar), 65.63 (*C*HCH$_3$), 24.61 (CH*C*H$_3$) ppm. MS-EI *m*/*z* = 309 (M^+). $[\alpha]_D^{20}$= +267.5. Anal. Calcd. for $C_{23}H_{19}N$: C, 89.28; H, 6.19; N, 4.53. Found: C, 88.96; H, 6.08; N, 4.49.

Imine **8**: **(*S*)-(+)-2-((((1-Cyclohexyl)ethyl)imino)methyl)naphthalene.** Mp 83 °C. FT-IR: ν=1634 cm^{-1} (C=N). ^{1}H NMR (500 MHz, $CDCl_3$/TMS): δ=8.36 (s, 1H; *H*C=N), 8.03 (s, 1H; Ar-*H*), 7.98-8.00 (m, 1H; Ar-*H*), 7.83-7.89 (m, 3H; Ar-*H*), 7-47-7.52 (m, 2H; Ar-*H*), 3.04-3.09 (m, 1H; C*H*CH$_3$), 1.83-1.87 (m, 1H; aliph-*H*), 1.63-1.79 (m, 4H; aliph-*H*), 1.49-1.56 (m, 1H; aliph-*H*), 1.26 (d, 3H; CH_3), 1.09-1.33 (m, 3H; aliph-*H*), 0.88-1.03 (m, 2H; aliph-*H*); ^{13}C NMR (500 MHz, $CDCl_3$/TMS): δ=158.78 (H*C*=N), 134.56, 134.22, 133.18, 129.44, 128.55, 128.35, 127.86, 126.91, 126.37, 124.21 (*C*-Ar), 72.20 (*C*HCH$_3$), 43.79, 30.08, 29.89, 26.63, 26.45, 26.29, 20.03 (*C* aliph) ppm. MS-EI *m*/*z* = 265 (M^+). $[\alpha]_D^{20}$=

+83.7. Anal. Calcd. for $C_{19}H_{23}N$: C, 85.99; H, 8.73; N, 5.28. Found: C, 85.85; H, 8.67; N, 5.05.

Imine **9**: **(*S*)-(–)-2-(((1,2,3,4-Tetrahydro-1-naphthyl)imino)methyl)naphthalene.** Mp 86 °C. FT-IR: ν=1630 cm^{-1} (C=N). ^{1}H NMR (500 MHz, $CDCl_3$/TMS): δ=8.55 (s, 1H; *H*C=N), 8.10 (s, 1H; Ar-*H*), 8.04-8.06 (m, 1H; Ar-*H*), 7.83-7.90 (m, 3H; Ar-*H*), 7.48-7.53 (m, 2H; Ar-*H*), 7.06-7.19 (m, 4H; Ar-*H*), 4.59 (t, 1H; aliph-*H*), 2.82-2.98 (m, 2H; aliph-*H*), 2.02-2.16 (m, 3H; aliph-*H*), 1.84-1.92 (m, 1H; aliph-*H*); ^{13}C NMR (500 MHz, $CDCl_3$/TMS): δ=160.70 (H*C*=N), 137.27, 137.19, 134.73, 134.03, 133.14, 129.98, 129.23, 128.67, 128.62, 128.43, 127.90, 127.11, 126.96, 126.46, 125.86, 124.27 (*C*-Ar), 68.73, 31.67, 29.58, 20.27 (*C*-aliph) ppm. MS-EI *m*/*z* = 285 (M^+). $[\alpha]_D^{20}$= -74.4. Anal. Calcd. for $C_{21}H_{19}N$: C, 88.38; H, 6.71; N, 4.91. Found: C, 87.97; H, 6.59; N, 4.88.

Imine **10**: **(+)-2-((((1*S*,2*S*,3*S*,5*R*)-2,6,6-Trimethylbicyclo[3.1.1]hept-3-yl)imino)methyl)naphthalene.** Mp 100 °C. FT-IR: ν=1634 cm^{-1} (C=N). ^{1}H NMR (500 MHz, $CDCl_3$/TMS): δ=8.33 (s, 1H; *H*C=N), 8.02-8.04 (m, 2H; Ar-*H*), 7.73-7.90 (m, 3H; Ar-*H*), 7.48-7.53 (m, 2H; Ar-*H*), 3.54-3.58 (m, 1H; aliph-*H*), 2.41-2.45 (m, 1H; aliph-*H*), 2.29-2.35 (m, 1H; aliph-*H*), 2.15-2.21 (m, 1H; aliph-*H*), 1.97-2.03 (m, 2H; aliph-*H*), 1.88-1.91 (m, 1H; aliph-*H*), 1.32 (d, 1H; aliph-*H*), 1.27 (s, 3H; CH_3), 1.11 (s, 3H; CH_3), 1.03 (d, 3H; CH_3); ^{13}C NMR (500 MHz, $CDCl_3$/TMS): δ=157.93 (H*C*=N), 134.53, 134.30, 133.17, 129.44, 128.54, 128.34, 127.87, 126.87, 126.35, 124.27 (*C*-Ar), 70.51, 47.61, 43.54, 41.76, 38.96, 36.01, 33.96, 28.08, 23.64, 19.93 (*C* aliph) ppm. MS-EI *m*/*z* = 291 (M^+); $[\alpha]_D^{20}$= +33.0. Anal. Calcd. for $C_{21}H_{25}N$: C, 86.55; H, 8.65; N, 4.81. Found: C, 85.99; H, 8.51; N, 4.74.
